# Supplementary material for: Tourmaline: A containerized workflow for rapid and iterable amplicon sequence analysis using QIIME 2 and Snakemake
Source: Gigascience. 2022 Jul 28;11:giac066. doi: 10.1093/gigascience/giac066 (PMC9334028; doi:10.1093/gigascience/giac066)
Supplement: giac066_GIGA-D-21-00281_Original_Submission [file giac066_giga-d-21-00281_original_submission.pdf]

GigaScience

Tourmaline: a workflow for rapid and reproducible amplicon sequence analysis using QIIME 2 and Snakemake

--Manuscript Draft--

**Manuscript Number:**	GIGA-D-21-00281
**Full Title:**	Tourmaline: a workflow for rapid and reproducible amplicon sequence analysis using QIIME 2 and Snakemake
**Article Type:**	Technical Note
**Funding Information:**	National Oceanic and Atmospheric Administration (NA06OAR4320264 06111039) — Dr. Luke R. Thompson<br>National Oceanic and Atmospheric Administration (NA17OAR4320152) — Not applicable<br>National Oceanic and Atmospheric Administration — Mr. Grant Sanderson, Dr. Kelly D. Goodwin
**Abstract:**	Background: Amplicon sequencing (metabarcoding) is a common method to survey diversity of environmental communities whereby a single genetic locus is amplified and sequenced from the DNA of whole or partial organisms, organismal traces (e.g., skin, mucus, feces), or microbes in an environmental sample. Several software packages exist for analyzing amplicon data, among which QIIME 2 has emerged as a popular option because of its broad functionality, plugin architecture, provenance tracking, and interactive visualizations. However, each new analysis requires the user to keep track of input and output file names, parameters, and commands; this lack of automation and standardization is inefficient and creates barriers to meta-analysis and sharing of results. Findings: We developed Tourmaline, a Python-based workflow for QIIME 2 built using the Snakemake workflow management system. Starting from a configuration file that defines parameters and input files—a reference database, a sample metadata file, and a manifest or archive of FASTQ sequences—it runs either the DADA2 or Deblur denoising algorithm, assigns taxonomy to the resulting representative sequences, performs analyses of taxonomic, alpha, and beta diversity, and generates an HTML report summarizing and linking to the output files. Features include automatic determination of trimming parameters using quality scores, representative sequence filtering (taxonomy, length, abundance, prevalence, or identifier), support for multiple taxonomic classification and sequence alignment methods, outlier detection, and automated initialization of a new analysis using previous settings. The workflow runs natively on Linux and macOS or via a Docker container. We ran Tourmaline on a 16S rRNA amplicon dataset from Lake Erie surface water, showing its utility for parameter optimization and the ability to easily evaluate results through the HTML report, QIIME 2 viewer, and R- and Python-based Jupyter notebooks. Conclusions: Reproducible workflows like Tourmaline enable rapid analysis of environmental and biomedical amplicon data, decreasing the time from data generation to actionable results. Tourmaline is available for download at github.com/aomlomics/tourmaline.
**Corresponding Author:**	Luke R. Thompson, Ph.D.<br>NOAA Atlantic Oceanographic and Meteorological Laboratory<br>Miami, Florida UNITED STATES
**Corresponding Author Secondary Information:**	
**Corresponding Author's Institution:**	NOAA Atlantic Oceanographic and Meteorological Laboratory
**Corresponding Author's Secondary Institution:**	
**First Author:**	Luke R. Thompson, Ph.D.
**First Author Secondary Information:**	
**Order of Authors:**	Luke R. Thompson, Ph.D.

Powered by Editorial Manager® and ProduXion Manager® from Aries Systems Corporation

	Sean R Anderson, Ph.D.
	Paul A. DenUyl
	Nastassia V. Patin, Ph.D.
	Grant Sanderson
	Kelly D. Goodwin, Ph.D.
**Order of Authors Secondary Information:**	
**Additional Information:**	
**Question**	**Response**
Are you submitting this manuscript to a special series or article collection?	No
**Experimental design and statistics**<br><br>Full details of the experimental design and statistical methods used should be given in the Methods section, as detailed in our Minimum Standards Reporting Checklist. Information essential to interpreting the data presented should be made available in the figure legends.<br><br>Have you included all the information requested in your manuscript?	Yes
**Resources**<br><br>A description of all resources used, including antibodies, cell lines, animals and software tools, with enough information to allow them to be uniquely identified, should be included in the Methods section. Authors are strongly encouraged to cite Research Resource Identifiers (RRIDs) for antibodies, model organisms and tools, where possible.<br><br>Have you included the information requested as detailed in our Minimum Standards Reporting Checklist?	Yes
**Availability of data and materials**<br><br>All datasets and code on which the conclusions of the paper rely must be	Yes

either included in your submission or deposited in publicly available repositories (where available and ethically appropriate), referencing such data using a unique identifier in the references and in the "Availability of Data and Materials" section of your manuscript.<br><br>Have you have met the above requirement as detailed in our Minimum Standards Reporting Checklist?	

Powered by Editorial Manager® and ProduXion Manager® from Aries Systems Corporation

Merged PDF
Click here to access/download;Manuscript;tourmaline_20210911.pdf

GigaScience, 2021, 1–14

doi: xx.xxxx/xxxx
Manuscript in Preparation
Technical Note

TECHNICAL NOTE

Tourmaline: a workflow for rapid and reproducible amplicon sequence analysis using QIIME 2 and Snakemake

Luke R. Thompson[1,2,*], Sean R. Anderson[1,2], Paul A. Den Uyl[3], Nastassia V. Patin[2,4,†], Grant Sanderson[5] and Kelly D. Goodwin[2,†]

[1]Northern Gulf Institute, Mississippi State University, Mississippi State, MS, USA and [2]Ocean Chemistry and Ecosystems Division, Atlantic Oceanographic and Meteorological Laboratory, National Oceanic and Atmospheric Administration, Miami, Florida, USA and [3]Cooperative Institute for Great Lakes Research, University of Michigan, Ann Arbor, MI, USA and [4]Cooperative Institute for Marine and Atmospheric Studies, Rosenstiel School of Marine and Atmospheric Science, University of Miami, Miami, FL, USA and [5]Marine Science Department, University of Hawaii, Hilo, HI, USA

[*]Correspondence: luke.thompson@noaa.gov
[†]Stationed at Southwest Fisheries Science Center, National Marine Fisheries Service, National Oceanic and Atmospheric Administration, La Jolla, CA, USA

Abstract

Background: Amplicon sequencing (metabarcoding) is a common method to survey diversity of environmental communities whereby a single genetic locus is amplified and sequenced from the DNA of whole or partial organisms, organismal traces (e.g., skin, mucus, feces), or microbes in an environmental sample. Several software packages exist for analyzing amplicon data, among which QIIME 2 has emerged as a popular option because of its broad functionality, plugin architecture, provenance tracking, and interactive visualizations. However, each new analysis requires the user to keep track of input and output file names, parameters, and commands; this lack of automation and standardization is inefficient and creates barriers to meta-analysis and sharing of results. **Findings:** We developed Tourmaline, a Python-based workflow for QIIME 2 built using the Snakemake workflow management system. Starting from a configuration file that defines parameters and input files—a reference database, a sample metadata file, and a manifest or archive of FASTQ sequences—it runs either the DADA2 or Deblur denoising algorithm, assigns taxonomy to the resulting representative sequences, performs analyses of taxonomic, alpha, and beta diversity, and generates an HTML report summarizing and linking to the output files. Features include automatic determination of trimming parameters using quality scores, representative sequence filtering (taxonomy, length, abundance, prevalence, or identifier), support for multiple taxonomic classification and sequence alignment methods, outlier detection, and automated initialization of a new analysis using previous settings. The workflow runs natively on Linux and macOS or via a Docker container. We ran Tourmaline on a 16S rRNA amplicon dataset from Lake Erie surface water, showing its utility for parameter optimization and the ability to easily evaluate results through the HTML report, QIIME 2 viewer, and R- and Python-based Jupyter notebooks. **Conclusions:** Reproducible workflows like Tourmaline enable rapid analysis of environmental and biomedical amplicon data, decreasing the time from data generation to actionable results. Tourmaline is available for download at github.com/aomlomics/tourmaline.

Key words: amplicon sequencing; metabarcoding; environmental DNA; eDNA; microbiome

Background

Earth's environments are teeming with environmental DNA (eDNA): free and cellular genetic material from whole microorganisms (HMP Consortium 2012; Thompson *et al.* 2017) or remnants of larger macroorganisms (Deiner *et al.* 2017; Compson *et al.* 2020). This eDNA can be collected, extracted, and sequenced to reveal the identities and functions of the organisms that produced it. Amplicon sequencing (metabarcoding), whereby a short genomic region is amplified and sequenced using polymerase chain reaction (PCR) from an environmental or experimental community's eDNA, is a popular method of measuring diversity in microbiomes and environmental samples (Zaiko *et al.* 2015; Deiner *et al.* 2017; Ruppert, Kline and Rahman 2019). PCR primers have been used to generate amplicons of the bacterial 16S rRNA gene in studies of human and animal microbiota (Turnbaugh *et al.* 2006; Ahn *et al.* 2013; Kartzinel *et al.* 2019) and environmental microbiota (Sunagawa *et al.* 2015; Thompson *et al.* 2017), the fungal internal transcribed spacer (ITS) of the rRNA gene (Abarenkov *et al.* 2010), the 18S rRNA gene of eukaryotic microorganisms and plankton (Vargas *et al.* 2015), the mitochondrial cytochrome oxidase I (COI) gene of invertebrate and vertebrate eDNA (Leray *et al.* 2013), and the mitochondrial 12S rRNA gene of fish (Miya *et al.* 2015), among others. Information gained from amplicon metabarcoding has far-reaching implications for human health (e.g., microbiome research), ecosystem function and conservation, and resource management.

Combining datasets from amplicon surveys and performing meta-analysis can reveal patterns impossible to observe from individual studies and also provide more power in statistical analyses. One example of the power of meta-analysis in amplicon surveys is the Earth Microbiome Project (Thompson *et al.* 2017), which used a single 16S rRNA amplicon method, metadata standard, and denoising algorithm to sequence and analyze over 25,000 microbial communities from around Earth. Standardization of methods is critical for comparability across studies. A lack of standardized methods is one of the main factors limiting cross-comparison of microbiome, eDNA, and other environmental surveys (Dickie *et al.* 2018; Harper *et al.* 2019). While standardizing detailed laboratory methods across many labs in multiple countries is a major challenge, standardizing analysis methods and metadata formats is much more feasible.

A popular approach for standardizing amplicon data analysis is to develop analysis pipelines or workflows (Prodan *et al.* 2020; Reiter *et al.* 2021) to run on local or networked computing resources or in the cloud. Examples include Anacapa (Curd *et al.* 2019), Banzai (https://github.com/jimmyodonnell/banzai), PEMA (Zafeiropoulos *et al.* 2020), Ampliseq (Straub *et al.* 2020), Cascabel (Asbun *et al.* 2019), and dadasnake (Weißbecker, Schnabel and Heintz-Buschart 2020). However, many pipelines are bespoke workflows with custom scripts for the user to navigate, and not all are well documented. A workflow that runs on top of a widely used amplicon software tool could take advantage of the functionality of the underlying tool and evolve with it, while remaining interoperable with any other analyses using this tool. If deployed on the cloud or in a container, it would also be portable enough to run on larger datasets.

QIIME 2 (Bolyen *et al.* 2019) is a popular software package that provides command-line, Python, and graphical user interfaces for amplicon sequence analysis from raw FASTQ sequences to observation tables, statistical analyses, and interactive visualizations. QIIME 2 supports DADA2 (Callahan *et al.* 2016) and Deblur (Amir *et al.* 2017) for denoising amplicon sequence data. Snakemake (Köster and Rahmann 2012) is a workflow management system that is popular in the bioinformatics community. Snakemake manages input and output files in a defined directory structure, with commands defined in a Snakefile as 'rules', and parameters and initial input files set by the user in a configuration file. Snakemake ensures that only the commands required for requested output files not yet generated are run, saving time and computation when re-running part of a workflow.

Here, we present Tourmaline (github.com/aomlomics/tourmaline), an amplicon analysis pipeline that uses Snakemake to wrap QIIME 2 commands and Python and Bash scripts to rapidly generate comprehensive analyses and interactive visualizations. After cloning the initial Tourmaline directory from GitHub and setting up the input files and parameters, only a few simple shell commands are required to execute the Tourmaline workflow. Outputs are stored in a standard directory structure that is the same for every Tourmaline run, facilitating data exploration and sharing, parameter optimization, and downstream analysis. Every Tourmaline run produces an HTML report containing a summary of metadata and outputs, with links to web-viewable QIIME 2 visualization files. QIIME 2 artifact files can be fed directly into Python- and R-based analysis packages. In addition to running natively on Mac and Linux platforms, Tourmaline can be run in any computing environment using Docker containers. In this paper, we describe the Tourmaline workflow and apply it to a subsampled 16S rRNA gene dataset from surface waters of Lake Erie. The tutorial includes guidance on evaluating output to refine parameters for the workflow and showcases the HTML report, interactive visualizations, and R- and Python-based analysis notebooks for biological insight into amplicon datasets.

Findings

The Tourmaline workflow

Overview. Tourmaline is a Snakemake-based bioinformatics workflow that operates in a defined directory structure (Fig. 1). Installation involves installing QIIME 2 and other dependencies or installing the Docker container. The starting directory structure is then cloned directly from GitHub and is built out through Snakemake commands, defined as 'rules' in the Snakefile. Tourmaline provides seven high-level 'pseudo-rules' for each of DADA2 paired-end, DADA2 single-end, and Deblur (single-end), running denoising and taxonomic and diversity analyses via QIIME 2 and other programs. For each type of processing, there are four steps: (1) the *denoise* rule imports FASTQ data and runs denoising, generating a feature table and representative sequences; (2) the *taxonomy* rule assigns taxonomy to representative sequences; (3) the *diversity* rule does representative sequence curation, core diversity analyses, and alpha and beta group significance; and (4) the *report* rule generates an HTML report of the metadata, inputs, outputs, and parameters. Steps 2–4 have two modes each, *unfiltered* and *filtered*, thus making seven pseudo-rules total. The difference between *unfiltered* and *filtered* modes is that in the *taxonomy* step of *filtered* mode, undesired taxonomic groups or individual sequences from the representative sequences and feature table are filtered (removed). The *diversity* and *report* rules are identical for *unfiltered* and *filtered* modes, except the outputs go

Compiled on: September 12, 2021.
Draft manuscript prepared by the author.

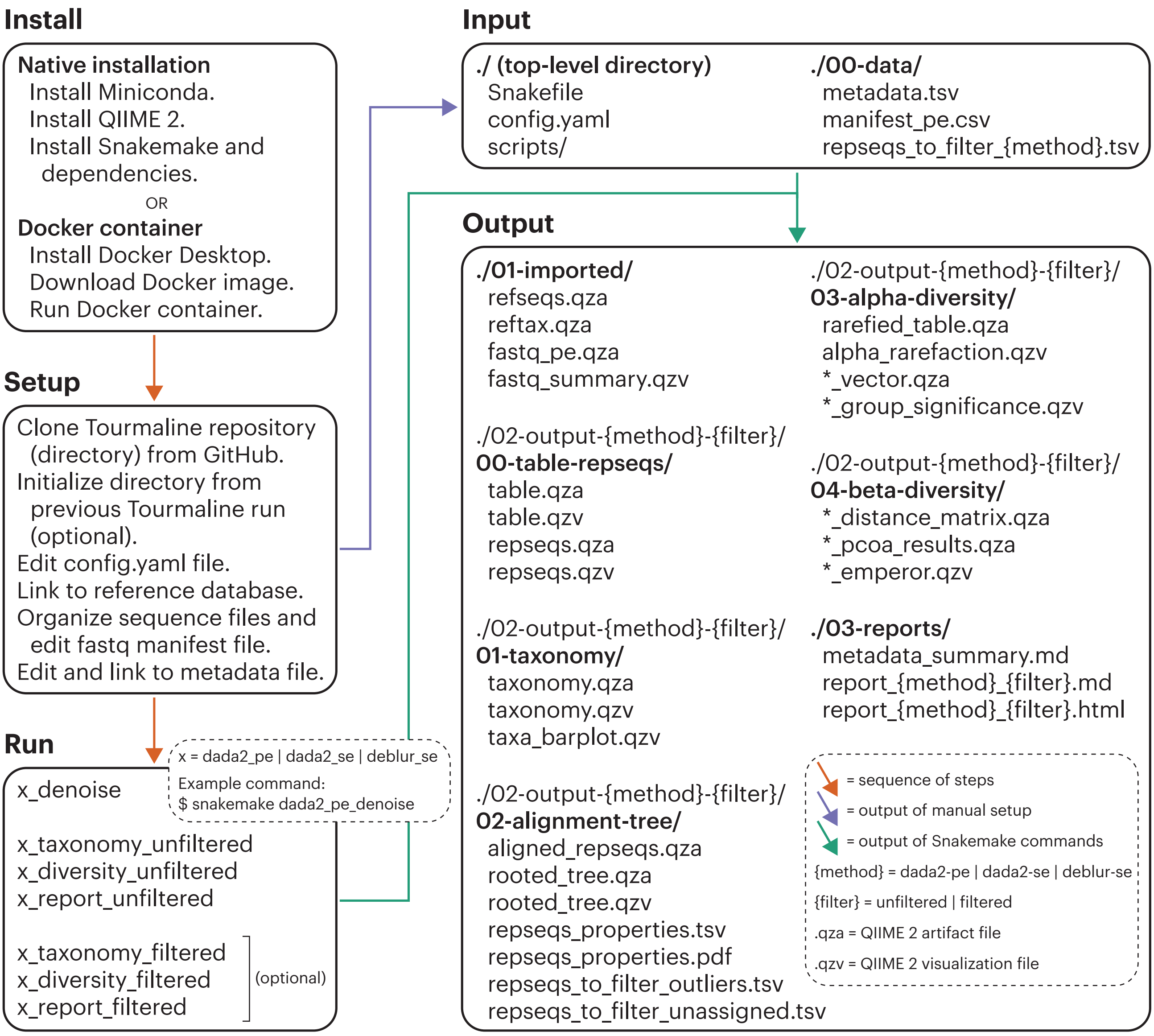

Figure 1. The Tourmaline workflow. Install natively (macOS, Linux) or using a Docker container. Setup by cloning the Tourmaline repository (directory) from GitHub, initializing the directory from a previous run (optional), editing the configuration file (*config.yaml*, Table S1), creating symbolic links to the reference database files, organizing the sequence files and/or editing the FASTQ manifest file, and editing and creating a symbolic link to the metadata file. Run by calling the Snakemake commands for *denoise*, *taxonomy*, *diversity*, and *report*—or running just the *report* command to generate all output if the parameters do not need to be changed between individual commands. It is recommended but not required to run in *unfiltered* mode before *filtered* mode. The primary input and output files are listed. Detailed instructions for each step are provided in the Tourmaline Wiki (github.com/aomlomics/tourmaline/wiki).

into separate subdirectories. In addition to the 21 pseudo-rules (3 denoising methods with 7 pseudo-rules each), there are 44 regular rules defined in *Snakefile* that perform the actual QIIME 2, Python, and shell commands of the workflow (Fig. S1).

Test dataset. Tourmaline comes with a test dataset of 16S rRNA gene (bacteria/archaea) amplicon data from surface waters of Western Lake Erie in summer 2018 (see Methods). The sequence data were subsampled to 1000 sequences per sample to allow the entire workflow to run in ~10 minutes. This test dataset is used in the tutorial and throughout the paper to demonstrate the capabilities of Tourmaline.

Documentation. Full instructions for using the Tourmaline workflow are described in the Tourmaline Wiki at github.com/aomlomics/tourmaline/wiki. Some experience with the command line, QIIME 2, and Snakemake is helpful to use Tourmaline; basic tutorials for each of these are provided at github.com/aomlomics/tutorials.

Installation. Before running the workflow (Fig. 1), the user should install QIIME 2 (version 2021.2) in a Conda environment, activate that environment, and then install the other dependencies of Tourmaline, including Snakemake, as described in the Wiki and README.md on GitHub. Alternatively, the user can create a Docker container using the Docker image from the repository *aomlomics/tourmaline* on DockerHub.

Cloning Tourmaline. After installing QIIME 2 and the other dependencies, or creating a Docker container from the Tourmaline Dockerfile, Tourmaline itself is installed simply by cloning the GitHub repository to the current directory with *git clone https://github.com/aomlomics/tourmaline*. This step will be repeated any time a new iteration of Tourmaline is needed. As described below, a helper script is provided to initialize a new Tourmaline directory with the files and settings of an existing Tourmaline directory, if so desired.

Snakefile. As a Snakemake workflow, Tourmaline has as its core two files: *Snakefile*, which provides all the commands (rules) that comprise the workflow; and *config.yaml*, which pro-

vides the input files and parameters for the workflow. *Snakefile* contains all of the commands used by Tourmaline, which invoke QIIME 2 commands or helper scripts (see below). The main analysis features and options supported by Tourmaline, as specified in *Snakefile*, are as follows:

- FASTQ sequence import using a manifest file, or use a pre-imported FASTQ .qza file.
- Denoising with DADA2 (Callahan *et al.* 2016) (paired-end and single-end) and Deblur (Amir *et al.* 2017) (single-end).
- Feature classification (taxonomic assignment) with options of naive Bayes (Bokulich *et al.* 2018), consensus BLAST (Camacho *et al.* 2008), and consensus VSEARCH (Rognes *et al.* 2016).
- Feature filtering by taxonomy, sequence length, feature ID, and/or abundance/prevalence.
- De novo multiple sequence alignment with MUSCLE (Edgar 2004), Clustal Omega (Sievers and Higgins 2014), or MAFFT (Katoh and Standley 2013) (with masking) and tree building with FastTree (Price, Dehal and Arkin 2009).
- Outlier detection with odseq (Jehl, Sievers and Higgins 2015).
- Interactive taxonomy barplot.
- Tree visualization using Empress (Cantrell *et al.* 2021).
- Alpha diversity, alpha rarefaction, and alpha group significance with four metrics: number of observed features, Faith's phylogenetic diversity, Shannon diversity, and Pielou's evenness.
- Beta diversity distances, principal coordinates, Emperor plots (Vázquez-Baeza *et al.* 2013), and beta group significance (one metadata column) with four metrics: unweighted and weighted UniFrac (Lozupone *et al.* 2010), Jaccard distance, and Bray–Curtis distance.

Config file. Next, the user should open the configuration file *config.yaml* in a text editor and carefully review all of the configuration settings. This includes paths to input files and parameters for QIIME 2 commands and other steps. If running the test data, the configurations can be left alone. If running user data, many of the parameters should be changed; see Table S1, Fig. 2, and the Wiki section *Setup* for guidance. For file paths, it is recommended to leave the default file paths as they are, remove the test files that come with the repository, and set symbolic links from the user's files to the default file paths. The helper script *initialize_dir_from_existing_tourmaline_dir.sh* can be run from inside a new Tourmaline directory to copy the files, symbolic links, and settings (*config.yaml*) from an existing Tourmaline directory.

Input files. The input files required by Tourmaline fall into three categories: (1) Reference database: a FASTA file of reference sequences (*refseqs.fna*) and a tab-delimited file of taxonomy (*reftax.tsv*) for those sequences, or their imported QIIME 2 artifact equivalents (*refseqs.qza*, *reftax.qza*); (2) Amplicon data: demultiplexed FASTQ sequence files and FASTQ manifest file(s) (*manifest_pe.csv*, *manifest_se.csv*) mapping sample names to the location of the sequence files, or their imported QIIME 2 equivalents (*fastq_pe.qza*, *fastq_se.qza*); and (3) Metadata: a tab-delimited sample metadata file (*metadata.tsv*) with sample names in the first column matching those in the FASTQ manifest file. The input files are stored in directory *00-data*, with two exceptions: the sequence files are recommended to be stored outside the Tourmaline directory because they tend to be large and shouldn't be copied for each new copy of Tourmaline (the test sequences can remain in *00-data* because they don't take up much disk space); and the imported QIIME 2 artifacts of the reference database files are stored in directory *01-imported* (Fig. 1). Any of these files can be located outside the Tourmaline directory, with symbolic links to them placed inside the Tourmaline directory, preferably preserving the default file names. Using symbolic links in this way prevents the user from having to copy large files to the Tourmaline directory.

Run the workflow. Once the configuration file has been edited and the input files (or symbolic links to them) placed in their appropriate directories, the workflow can be run using Snakemake commands. For the examples in this paper, we will use the DADA2 paired-end method; to use the DADA2 single-end method or Deblur single-end method, change *dada2_pe* in the commands below to *dada2_se* or *deblur_se*. Note that any of the commands below can be run with various options, including *--printshellcmds* to see the shell commands being executed and *--dryrun* to display which rules would be run but not execute them. If the user is confident that all parameters are correct, for example to quickly run the workflow on the test data, the entire workflow can be run with *snakemake dada2_pe_report_unfiltered* or *snakemake dada2_pe_report_filtered*. However, in most cases it will be prudent to run the shorter pseudo-rules (commands) individually and examine the output, as described below.

Tutorial

This section walks through a tutorial on Tourmaline using the Lake Erie test data that comes with the GitHub repository. For each command, the main parameters used and output files generated in those commands are listed (Fig. 2). Accompanying the output files is guidance for evaluating them to choose parameters for subsequent steps (Fig. 2), with screenshots of the output files shown (Fig. 3). A video version of the tutorial is available on YouTube (https://youtu.be/xKfOxrXBXYQ).

Denoise. The first command is *snakemake dada2_pe_denoise* (Fig. 2), which imports the FASTQ files and reference database (if not already present in directory *01-imported*), summarizes the FASTQ data, runs denoising using DADA2, and summarizes the output. In addition to QIIME 2 visualizations of the FASTQ summary (Fig. 3A), feature table (Fig. 3B), representative sequences (Fig. 3C), and phylogenetic tree (Fig. 3D), Tourmaline generates a scatter plot and table—*repseqs_properties.pdf* (Fig. 3E) and *repseqs_properties.tsv* (Fig. 3F)—of representative sequence properties, including sequence length, number of gaps in the multiple sequence alignment, outlier status, taxonomy, and total number of observations in the observation table. At this point, the user should examine *fastq_summary.qzv* (Fig. 3A) for quality scores and *reqseqs.qzv* (Fig. 3C) or *repseqs_properties.tsv* (Fig. 3F) for representative sequence lengths; additionally, the helper script *fastqc_per_base_sequence_quality_dropoff.py* can be run on the output of FastQC and MultiQC to estimate optimal truncation lengths (see 'Helper scripts' below). If it is determined that the DADA2 or Deblur truncation or trim parameters need to be modified, the user should delete the output files and rerun the *denoise* step. Based on the representative sequence lengths, filtering by sequence length can also be set, to be used later in *filtered* mode. The user should also examine *table.qzv* (Fig. 3B) and choose appropriate sampling (rarefaction) depths for the parameters 'alpha_max_depth' and 'core_sampling_depth', to be used in the *diversity* step.

Taxonomy. The second command is *snakemake dada2_pe_taxonomy_unfiltered* (Fig. 2), which assigns taxonomy to the representative sequences using a naive Bayes classifier or consensus BLAST or VSEARCH method and generates an interactive taxonomy table and an interactive barplot of sample taxonomic composition. The user should examine *taxonomy.qzv* (Fig. 3G) and *taxa_barplot.qzv* (Fig. 3E) to determine if any taxonomic groups should be filtered by

Parameters in config.yaml | **Output to evaluate**

snakemake dada2_pe_denoise →

use manifest file to import fastq.gz sequence files
manifest_pe: 00-data/manifest_pe.csv

use pre-imported reference database
refseqs_qza: 01-imported/refseqs.qza
reftax_qza: 01-imported/reftax.qza

choose dada2 parameters based on fastq error profiles
dada2pe_trunc_len_f: 240
dada2pe_trunc_len_r: 190

fastq_summary.qzv
• median Q-score <30 occurs at fwd. position 267 and rev. position 233 -> trimming at 240 and 190 is acceptable for this amplicon (<300 bp)
• 16 samples (fwd. & rev.) all have 1000 reads per sample (test dataset)

repseqs.qzv & repseqs_lengths.tsv
• of 301 repseqs, most are 253 bp and max is 255 bp except two that are much longer (416 bp, 417 bp) -> filter by length max 260 bp

table.qzv
• of 16 samples, lowest count per sample is 511 -> set core sampling depth (rarefaction) to 500 (check again after filtering)

snakemake dada2_pe_taxonomy_unfiltered →

choose taxonomic classification method (*)
classify_method: consensus-vsearch

taxonomy.qzv
• 10 repseqs are "Unassigned" and 2 repseqs are "d__Eukaryota" -> filter by keywords "unassigned,eukaryota"

taxa_barplot.qzv
• the contribution of Unassigned and Eukaryota groups is <10%; still want to filter them

snakemake dada2_pe_diversity_unfiltered →

choose MSA parameters (*)
alignment_method: muscle
alignment_muscle_maxiters: 2
alignment_muscle_diags: -diags

choose outlier detection parameters (*)
odseq_distance_metric: linear
odseq_bootstrap_replicates: 100
odseq_threshold: 0.025

choose sampling (rarefaction) depth
core_sampling_depth: 500
alpha_max_depth: 500

choose beta group significance parameters (*)
beta_group_column: region
beta_group_method: permanova
beta_group_pairwise: --p-pairwise

rooted_tree.qzv
• feature metadata coloring confirms we should filter Unassigned and Eukaryota

repseqs_properties.pdf
• confirms we should filter Unassigned and Eukaryota and sequences longer than 260 bp; don't need to filter all outliers

alpha_rarefaction.qzv
• observed features plateaus at ~450–500 sequences per sample

observed_features_group_significance.qzv
• difference between regions (Open Water vs. Western Boundary) is not significant by Kruskal–Wallis, but filter size is significant

unweighted_unifrac_emperor.qzv
• separation by region (axis 2) and filter size (axes 1 & 2)

beta_group_significance.qzv
• distance based on region is significant

snakemake dada2_pe_report_unfiltered →

choose theme for html report
report_theme: github

report_dada2-pe_unfiltered.html
• a summary of the results and metadata and links to output files are presented in this HTML report

snakemake dada2_pe_report_filtered (**) →

choose terms to filter from taxonomy
exclude_terms: unassigned,eukaryota

choose repseq length limits
repseq_min_length: 0
repseq_max_length: 260

table.qzv
• of 16 samples, lowest count per sample is 507 -> it was ok to leave sampling (rarefaction) depth at 500

rooted_tree.qzv
• feature metadata coloring confirms Unassigned and Eukaryota were removed, and tree topology is more homogeneous

repseqs_properties.pdf
• confirms long sequences and Unassigned and Eukaryota were removed, resulting in fewer gaps in the multiple sequence alignment

report_dada2-pe_filtered.html
• a summary of the results and metadata and links to output files are presented in this HTML report

(*) these steps can be defined before starting the workflow, as they do not depend on the output of previous steps

(**) all steps are being run at once by using the report command

Figure 2. Step-by-step tutorial on Tourmaline using the provided test data, which is subsampled 16S data from a 2018 survey of Lake Erie. Key parameters in *config.yaml* and primary output for each command (pseudo-rule) are listed. Indicated output should be evaluated to determine the appropriate parameters for the next command. Evaluation of the primary outputs and rationale for parameter choice is shown for the test Lake Erie 16S rRNA data that comes with the Tourmaline repository. See Fig. 3 for screenshots of the primary output files.

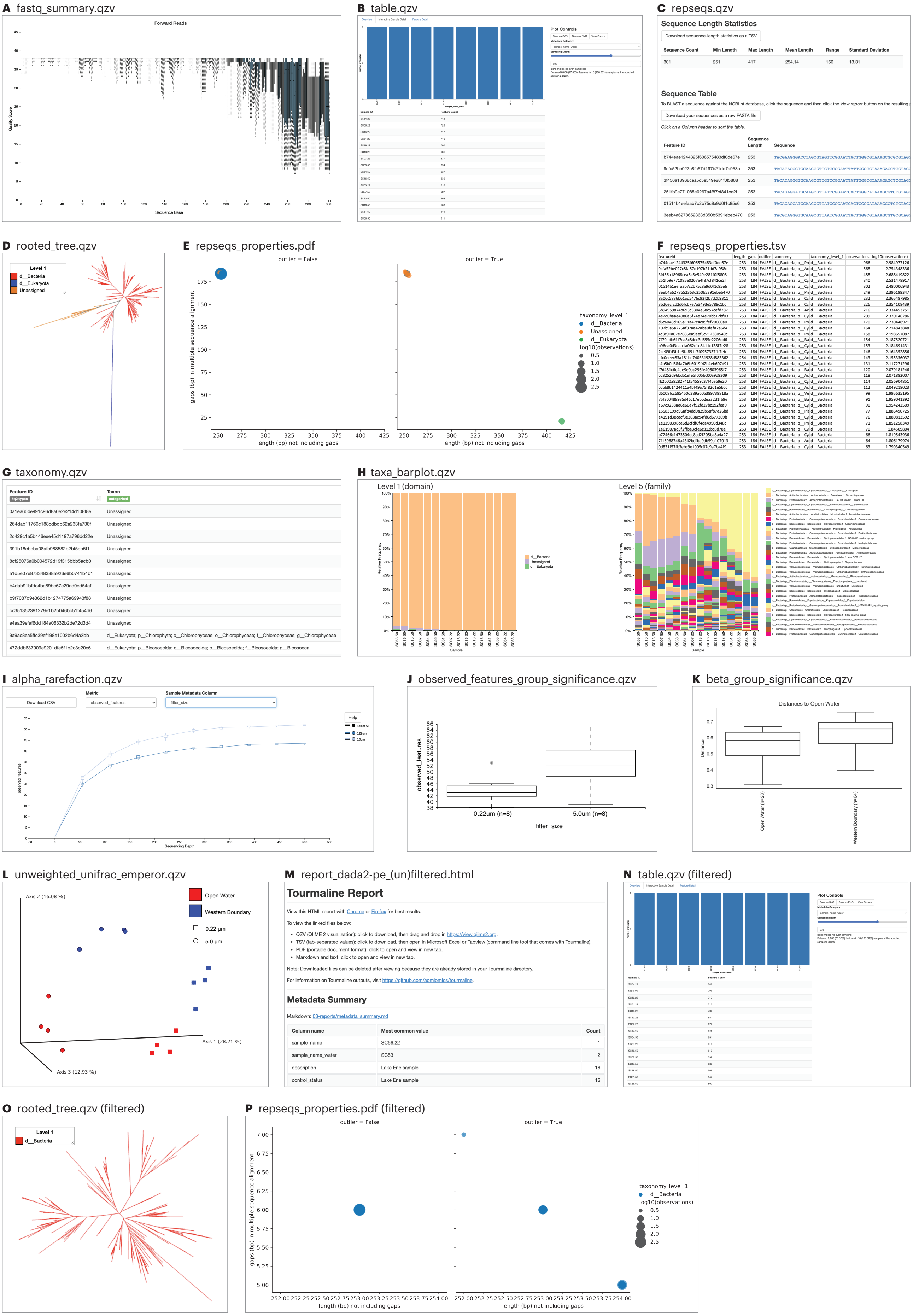

Figure 3. Screenshots of the primary output files after running Tourmaline on the test data (see Fig. 2 for commands, parameters, and guidance). The QIIME 2 visualizations (.qsv) and Tourmaline-specific tables (.tsv), plots (.pdf), and report (.html) support data evaluation, data discovery, and biological insight.

keyword, to be used later in *filtered* mode.

Diversity. The third command is *snakemake dada2_pe_diversity_unfiltered* (Fig. 2), which aligns representative sequences using one of three methods, computes outliers using odseq (Jehl, Sievers and Higgins 2015), and builds a phylogenetic tree. This step generates lists of representative sequences that have unassigned taxonomy and were computed to be outliers, summarizes and plots the representative sequence properties, performs alpha rarefaction, and runs alpha diversity and beta diversity analyses and group significance tests using a suite of metrics. The user should examine *rooted_tree.qzv* (Fig. 3D) and *repseqs_properties.pdf* (Fig. 3E) to check filtering parameters, if desired. Whether sampling depth was sufficient can be evaluated with *alpha_rarefaction.qzv* (Fig. 3I). Alpha and beta diversity patterns and statistically significant differences between groups can be evaluated with *observed_features_group_significance.qzv* (Fig. 3J; other alpha diversity metrics are also provided), *beta_group_significance.qzv* (Fig. 3K), and *unweighted_unifrac_emperor.qzv* (Fig. 3L; other beta diversity metrics are also provided).

Report. The fourth and final command is *snakemake dada2_pe_report_unfiltered* (Fig. 2), which creates a comprehensive HTML report of parameters, metadata, inputs, outputs, and visualizations in a single file. The file *report_dada2-pe_unfiltered.html* (Fig. 3M) can be viewed in a web browser.

Filtering. Filtering of representative sequences is provided by the *filtered* mode. After reviewing the *unfiltered* results—the taxonomy summary and taxa barplot, the representative sequence summary plot and table, or the list of unassigned and potential outlier representative sequences—the user may wish to filter (remove) certain representative sequences by taxonomic group or other properties. If so, the user should first edit *config.yaml* to ensure that any taxa desired for exclusion are listed in 'exclude_terms'; set values for 'repseq_min_length', 'repseq_max_length', 'repseq_min_abundance', and/or 'repseq_min_prevalence'; and/or choose feature IDs (rows) from *repseqs_to_filter_unassigned.tsv* and *repseqs_to_filter_outliers.tsv* to include in *repseqs_to_filter_dada2-pe.tsv* in *00-data*. Note that if the user wants to filter by criteria other than taxonomic group or feature ID, respectively, the value for 'exclude_terms' in *config.yaml* should be a nonsense word (not found in any taxon name) or the feature IDs in *repseqs_to_filter_dada2-pe.tsv* should be nonsense IDs (not real feature IDs). The user can then run the *filtered* mode of the workflow starting with *snakemake dada2_pe_taxonomy_filtered*, followed by *snakemake dada2_pe_diversity_filtered* and finally *snakemake dada2_pe_report_filtered*; alternatively, all three steps can be run at once by running *snakemake dada2_pe_report_filtered* (Fig. 2). Examining the *filtered* output, the user should check *table.qzv* (Fig. 3N) to ensure that the sampling depth after filtering did not exclude samples, and examine *rooted_tree.qzv* (Fig. 3O) and *repseqs_properties.pdf* (Fig. 3P) to check that the desired representative sequences were filtered. All of the outputs can be viewed by opening *report_dada2-pe_filtered.html* (Fig. 3M) in a web browser.

Helper scripts

Tourmaline comes with several helper scripts that are run automatically within the workflow or run directly by the user.

Initialize a new Tourmaline directory. From the main directory of a newly cloned tourmaline directory, run the script *initialize_dir_from_existing_tourmaline_dir.sh* to copy *config.yaml* and *Snakefile* from an existing tourmaline directory, remove the test files, then copy the data files and symlinks from the existing tourmaline directory. This is useful when re-running an analysis on the same dataset. Simply clone a new copy of Tourmaline, then run this script to copy everything from the old Tourmaline directory to the new one, then make your desired changes to the parameters.

Create a FASTQ manifest file. Two scripts help create the manifest file that points Tourmaline to the FASTQ sequence files. To create a FASTQ manifest file from a directory of FASTQ files, run *create_manifest_from_fastq_directory.py*. This script makes the following assumptions: the first characters after the sample names are *_S[0-9]{1,3}*; only Lane 1 data is present (*_L001*); R1 and R2 files are both present; and only one file (with suffix *_001*) is present for each of R1 and R2. Another option is to run *match_manifest_to_metadata.py*, which given a metadata file and a FASTQ manifest file, generates two new manifest files (paired-end and single-end) corresponding to the samples in the metadata file. This can be useful if you demultiplexed from 'Earth Microbiome Project (EMP) protocol' format for paired-end reads and unzipped the output to obtain the manifest file.

Determine optimal truncation length. After running FastQC and MultiQC separately for Read 1 and Read 2 (see instructions in the Wiki), run *fastqc_per_base_sequence_quality_dropoff.py* to determine the position where median per-base sequence quality drops below some fraction (default: 0.90) of its maximum value. This is useful for defining 3' truncation positions in DADA2 and Deblur ('dada2pe_trunc_len_f', 'dada2se_trunc_len', and 'deblur_trim_length').

Visualizations & outputs

HTML reports. Each Tourmaline run produces an HTML report (*03-reports/report_{method}_{filter}.html*) that is viewable in a web browser. To view .qzv files (QIIME 2 visualizations), click to download, then drag and drop in view.qiime2.org. Empress trees (e.g., *rooted_tree.qzv*) may take more than 10 minutes to load. To view .tsv files (tab-separated values), click to download, then open in Microsoft Excel or Tabview (command line tool that comes with Tourmaline). To view .pdf files (portable document format), click to open and view in a new tab. Downloaded files can be deleted after viewing because they are already stored in your Tourmaline directory.

QIIME 2 visualizations and artifacts. Tourmaline produces interactive QIIME 2 visualization (.qzv) files and non-interactive QIIME 2 artifact (.qza) files. Both file types can be viewed at view.qiime2.org, where file details and provenance are displayed, and especially where .qzv files can be visualized and interacted with. Both file types can also be opened within a Jupyter notebook (described below). Additionally, because both file types are ZIP archives, they can be unzipped to reveal the packaged data.

Jupyter notebooks. For users who wish to analyze their output further using Jupyter notebooks, we provide Python and R notebooks (Fig. S2) pre-loaded with popular data analysis and visualization tools for those platforms. These notebooks come ready to run with Tourmaline output, using relative paths to take advantage of Tourmaline's defined output file structure. The notebooks are shown with the tutorial dataset that comes with Tourmaline.

The Python Jupyter notebook (Fig. S2) uses the QIIME 2 Visualization and Artifact object classes, loading Visualization and Artifact objects from the .qzv and .qza Tourmaline output files. Before running the notebook, the denoising method, filtering mode, and alpha and beta diversity metrics to be used can be specified by changing variable assignments at the beginning of the notebook. The notebook renders Visualization objects for the feature table summary, representative sequences

summary, phylogenetic tree, taxonomy, taxa bar plot, alpha diversity group significance, and beta diversity principal coordinates (PCoA) Emperor plot. Artifact objects can be viewed as a Pandas (McKinney 2010) DataFrame or Series. The notebook generates Pandas DataFrames for the feature table, taxonomy, reference sequence properties, and metadata, and a Pandas Series for alpha diversity. Static plots are generated from some of these tables using Seaborn (Qalieh *et al.* 2017).

The R Jupyter notebook (Fig. S2) imports Tourmaline artifact (.qza) files using qiime2R (Bisanz 2018) and uses common R packages for analyzing and visualizing amplicon sequencing data, including phyloseq (Halfvarson *et al.* 2017), tidyverse (Wickham *et al.* 2019), and vegan (Oksanen *et al.* 2020). The notebook covers how to import QIIME 2 count and taxonomy artifact files from Tourmaline into an R environment, merge and manipulate the resulting data frames into a single phyloseq object, and estimate and plot diversity metrics and taxonomy bar plots of the 16S community using phyloseq and other packages. As with the Python notebook, a set of variables can be specified at the beginning of the R notebook to define specific denoising, filtering, and diversity metrics. After reading in the metadata file and merging to a phyloseq object, we define plotting parameters that can be easily modified by the user to customize the R visualizations.

Biological insights

The purpose of performing amplicon sequencing or metabarcoding is to reveal patterns of diversity in biological systems. Whether the system of study is microbial communities in an environmental or biomedical setting or trace environmental DNA in an aquatic or terrestrial system, the kinds of biological questions being asked are similar. Tourmaline supports biological insight in two important ways: (1) by supporting the most popular analysis tools and packages in use today, with capacity to expand as new tools are developed; (2) by providing multiple ways to view the output, giving everyone from experts to novices a platform to visualize and query the output.

Through its core QIIME 2 functionality and downstream support for R and Python data science packages, Tourmaline enables analysis of the core metrics of microbial and eDNA diversity: taxonomic composition, within-sample diversity (alpha diversity), and between-sample diversity (beta diversity). Examining our analysis of the tutorial dataset (Fig. 3), we can see how Tourmaline provides rapid insight into these Western Lake Erie microbial communities. The interactive barplot (Fig. 3H) provides rapid insights: the most abundant bacterial families in the 5.0-μm fraction are Sporichthyaceae and SAR11 Clade III; the most abundant bacterial family in the 0.22-μm fraction is Cyanobiaceae (the toxic cyanobacterial family Microcystaceae is less abundant), with the largest component assigned as chloroplasts, which can be filtered in a subsequent run; at the domain level, a small fraction of unassigned and Eukaryota-assigned sequences are observed, which can also be filtered. The alpha diversity results show that the 5.0-μm fraction has greater within-sample diversity (number of observed features) than the 0.22-μm fraction (Fig. 3J) and that this diversity appears to be saturated, with a relatively small sampling depth of ~350 sequences per sample sufficient to observe these values (Fig. 3I). However, because a large fraction of the 0.22-μm sequences were identified as chloroplast, filtering out those sequences in a future run would be warranted and provide more accurate diversity results. The beta diversity results show that 16S communities are distinguished both by location (Open Water vs. Western Boundary) and size fraction (0.22-μm vs. 5.0-μm) (Fig. 3K and Fig. 3L). Thus, from this simple tutorial dataset, we see the value of Tourmaline to analyze environmental amplicon data, in this case revealing the importance of pore size when filtering water samples for microbial sequencing and the presence of spatial variability (regardless of pore size) among microbial communities in Lake Erie.

The ability to view Tourmaline output files with multiple interfaces provides access to researchers with different backgrounds. For users experienced with the Unix command line, the diverse output file types, organized in a defined directory structure, can be queried and analyzed using a wide array of data science tools; anything that can be done with QIIME 2 output and other common sequence diversity output files types can be done with Tourmaline output. For data scientists most comfortable with Jupyter notebooks, the prebuilt Python and R notebooks come ready to work with Tourmaline output and enable rapid biological discovery from amplicon data. For casual users, the web-based report and QIIME 2 visualizations provide a user-friendly onramp to view and interact with the data. This last mode of interacting with the output opens up amplicon analysis to a wider range of users than is typically possible, from collaborators to students to anyone with limited data science expertise. This increased accessibility can accelerate the pace of discovery by increasing the diversity of researchers able to work with amplicon analysis results.

Conclusions

Tourmaline provides a comprehensive platform for amplicon sequence analysis that enables rapid and reproducible processing and inference of microbiome and eDNA metabarcoding data. It has multiple features that enhance usability and interoperability:

- **Portability.** Native support for Linux and macOS in addition to Docker containers, enabling it to run on desktop, cluster, and cloud computing platforms.
- **QIIME 2.** The core commands of Tourmaline, including the DADA2 and Deblur packages, are all commands of QIIME 2, one of the most popular amplicon sequence analysis software tools available. Users can print all of the QIIME 2 and other shell commands of a workflow before or while running the workflow.
- **Snakemake.** Managing the workflow with Snakemake provides several benefits:
  - **Configuration file** contains all parameters in one file, so the user can see what the workflow is doing and make changes for a subsequent run.
  - **Directory structure** is the same for every Tourmaline run, so the user always knows where outputs are.
  - **On-demand commands** mean that only the commands required for output files not yet generated are run, saving time and computation when re-running part of a workflow.
- **Parameter optimization.** The configuration file and standard directory structure make it simple to test and compare different parameter sets to optimize the workflow. Included code helps choose read truncation parameters and identify outliers in representative sequences (ASVs).
- **Visualizations and reports.** Every Tourmaline run produces an HTML report containing a summary of metadata and outputs, with links to web-viewable QIIME 2 visualization files.
- **Downstream analysis.** Analyze the output of single or multiple Tourmaline runs programmatically, with qiime2R in R or the QIIME 2 Artifact API in Python, using the provided R and Python notebooks or other code.

Through its streamlined workflow and broad functionality, Tourmaline enables rapid response and biological discovery in any system where amplicon sequencing is applied, from biomedical and environmental microbiology to eDNA for fisheries and protected or invasive species. The QIIME 2-based interactive visualizations it generates allow users to quickly compare differences between samples and groups of samples in their taxonomic composition, within-sample (alpha) diversity, and between-sample (beta) diversity, which are core metrics of microbial and eDNA diversity. Tourmaline's unique HTML report and pre-loaded Jupyter notebooks provide ready access to the output, supporting less-experienced researchers and data scientists alike, and the output files are ready to be loaded into a variety of downstream tools in the QIIME 2 and phyloseq ecosystems. By balancing usability, functionality, and reproducibility, Tourmaline is a powerful workflow that brings diversity analysis to more users.

Methods

Sample collection and DNA extraction

Water samples were collected using a long-range autonomous underwater vehicle (LRAUV, Monterey Bay Aquarium Research Institute) equipped with a third-generation environmental sample processor (3G-ESP, Monterey Bay Aquarium Research Institute) (Pargett *et al.* 2015). For each sample, water was filtered through stacked 5.0-μm (top) and 0.22-μM (bottom) Durapore filters (EMD Millipore) held in custom 3G-ESP 'archive' cartridges and preserved in-cartridge with RNAlater (Thermo Fisher). DNA extraction was performed using the Qiagen DNeasy Blood and Tissue kit.

Amplicon sequencing

Extracted DNA was amplified using a BiooScientific NEXTFlex 16S V4 Amplicon-Seq Kit 2.0 (NOVA-520999/Custom NOVA-4203-04] (BiooScientific, Austin, TX, USA). Target-specific regions of the forward and reverse primers in the 16S V4 Amplicon-Seq kit were custom ordered to follow the Earth Microbiome Project 16S Illumina Amplicon Protocol: forward primer 515F 5'-GTGYCAGCMGCCGCGGTAA-3' (Parada, Needham and Fuhrman 2016) and reverse primer 806R 5'-GGACTACNVGGGTWTCTAAT-3' (Apprill *et al.* 2015). 16S amplicons were pooled and sequenced at the University of Michigan Advanced Genomics Core (brcf.medicine.umich.edu/cores/dna-sequencing). Demultiplexed sequences were deposited in NCBI under BioProject ID PRJNA679730.

Supporting tables, figures, and videos

Supporting tables and figures are attached to the end of this manuscript.

Video 1. Tutorial covering installing and running the Tourmaline workflow, reviewing the report, and viewing QIIME 2 visualization files. https://youtu.be/xKfOxrXBXYQ

Availability of supporting source code

- Tourmaline is available by cloning the GitHub repository at https://github.com/aomlomics/tourmaline.
- Full installation and usage instructions are available from the Tourmaline Wiki at https://github.com/aomlomics/tourmaline/wiki.
- Docker image is available from DockerHub at https://hub.docker.com/r/aomlomics/tourmaline.
- Additional code and data from the full 2018 Lake Erie dataset is available at https://github.com/aomlomics/erie.

Availability of supporting data

- The test 16S dataset (1000 sequences per sample) is available directly from the GitHub repository at https://github.com/aomlomics/tourmaline.
- Reference database files for the 16S rRNA gene are available at https://docs.qiime2.org/2021.2/data-resources/#silva-16s-18s-rrna.
- Output for the tutorial using the included test data are available from Zenodo at https://doi.org/10.5281/zenodo.5044532.
- A snapshot of the GitHub repository will be available from Zenodo upon publication.

Abbreviations

ASV: amplicon sequence variant; COI: cytochrome oxidase I; DAG: directed acyclic graph; eDNA: environmental DNA; ITS: internal transcribed spacer; PCoA: principal coordinates analysis; PCR: polymerase chain reaction; QIIME: Quantitative Insights Into Microbial Ecology.

Competing interests

The authors declare that they have no competing interests.

Funding

This work was supported by awards NA06OAR4320264 06111039 to the Northern Gulf Institute (NGI) at Mississippi State University, NA17OAR4320152 (contribution number 1168) to the Cooperative Institute for Great Lakes Research (CIGLR) at the University of Michigan, and a National Oceanographic Partnership Program grant to the Cooperative Institute for Marine and Atmospheric Studies (CIMAS) at the University of Miami from NOAA's Office of Oceanic and Atmospheric Research (OAR), U.S. Department of Commerce. Support was also provided by the OAR 'Omics Program, Ocean Technology Development, and the National Oceanographic Partnership Program (NOPP). G. Sanderson contributed to this work as part of a NOAA Ernest F. Hollings Scholarship summer internship.

Author contributions

The Tourmaline workflow was designed and developed by L.R. Thompson. Code was tested by L.R. Thompson, N.V. Patin, and S.R. Anderson. The Docker image was built by N.V. Patin and L.R. Thompson. Data analysis and visualization of the case study were done by S.R. Anderson. Analysis notebooks were developed by L.R. Thompson, S.R. Anderson, and G. Sanderson. Samples were collected by P.A. Den Uyl and K.D. Goodwin. DNA was extracted and prepared for sequencing by P.A. Den Uyl. The manuscript was written by L.R. Thompson, S.R. Anderson, P.A. Den Uyl, and K.D. Goodwin.

Acknowledgements

We thank Mehrbod Estaki and Jean Lim for testing the Tourmaline workflow and feedback on the Tourmaline GitHub repository. We thank Jean Lim for helpful comments on the manuscript. We also thank Reagan Errera, Subba Rao Chaganti, Jim Birch, Greg Doucette, for project planning and sample and data collection for the Lake Erie 3G-ESP project. We thank Gregory Dick, Colleen Yancey, and McKenzie Powers for discussions on *Microcystis* diversity and genomics.

References

Abarenkov K, Nilsson RH, Larsson K *et al.* The UNITE database for molecular identification of fungi – recent updates and future perspectives. *New Phytologist* 2010;**186**:281–5.

Ahn J, Sinha R, Pei Z *et al.* Human Gut Microbiome and Risk for Colorectal Cancer. *JNCI: Journal of the National Cancer Institute* 2013;**105**:1907–11.

Amir A, McDonald D, Navas-Molina JA *et al.* Deblur rapidly resolves single-nucleotide community sequence patterns. *mSystems* 2017;**2**, DOI: 10.1128/msystems.00191-16.

Apprill A, McNally S, Parsons R *et al.* Minor revision to V4 region SSU rRNA 806R gene primer greatly increases detection of SAR11 bacterioplankton. *Aquatic Microbial Ecology* 2015;**75**:129–37.

Asbun AA, Besseling MA, Balzano S *et al.* Cascabel: a flexible, scalable and easy-to-use amplicon sequence data analysis pipeline. *bioRxiv* 2019:809384.

Bisanz JE. qiime2R: Importing QIIME2 artifacts and associated data into R sessions. 2018.

Bokulich NA, Kaehler BD, Rideout JR *et al.* Optimizing taxonomic classification of marker-gene amplicon sequences with QIIME 2's q2-feature-classifier plugin. *Microbiome* 2018;**6**:90.

Bolyen E, Rideout JR, Dillon MR *et al.* Reproducible, interactive, scalable and extensible microbiome data science using QIIME 2. *Nature Biotechnology* 2019;**37**:852–7.

Callahan BJ, McMurdie PJ, Rosen MJ *et al.* DADA2: High-resolution sample inference from Illumina amplicon data. *Nature Methods* 2016;**13**:581–3.

Camacho C, Coulouris G, Avagyan V *et al.* BLAST+: architecture and applications. *BMC Bioinformatics* 2008;**10**:421–1.

Cantrell K, Fedarko MW, Rahman G *et al.* EMPress Enables Tree-Guided, Interactive, and Exploratory Analyses of Multi-omic Data Sets. *mSystems* 2021;**6**, DOI: 10.1128/msystems.01216-20.

Compson ZG, McClenaghan B, Singer GAC *et al.* Metabarcoding From Microbes to Mammals: Comprehensive Bioassessment on a Global Scale. *Frontiers in Ecology and Evolution* 2020;**8**:581835.

Curd EE, Gold Z, Kandlikar GS *et al.* Anacapa Toolkit: an environmental DNAtoolkit for processing multilocus metabarcode datasets. *Methods in Ecology and Evolution* 2019:2041-210X.13214.

Deiner K, Bik HM, Mächler E *et al.* Environmental DNA metabarcoding: Transforming how we survey animal and plant communities. *Molecular ecology* 2017;**26**:5872–95.

Dickie IA, Boyer S, Buckley HL *et al.* Towards robust and repeatable sampling methods in eDNA-based studies. *Molecular Ecology Resources* 2018;**18**:940–52.

Edgar RC. MUSCLE: multiple sequence alignment with high accuracy and high throughput. *Nucleic Acids Research* 2004;**32**:1792–7.

Halfvarson J, Brislawn CJ, Lamendella R *et al.* Dynamics of the human gut microbiome in inflammatory bowel disease. *Nature Microbiology* 2017;**2**:17004.

Harper LR, Buxton AS, Rees HC *et al.* Prospects and challenges of environmental DNA (eDNA) monitoring in freshwater ponds. *Hydrobiologia* 2019;**826**:25–41.

Human Microbiome Project Consortium. Structure, function and diversity of the healthy human microbiome. *Nature* 2012;**486**:207–14.

Jehl P, Sievers F, Higgins DG. OD-seq: outlier detection in multiple sequence alignments. *BMC Bioinformatics* 2015;**16**:269.

Kartzinel TR, Hsing JC, Musili PM *et al.* Covariation of diet and gut microbiome in African megafauna. *Proceedings of the National Academy of Sciences* 2019;**116**:23588–93.

Katoh K, Standley DM. MAFFT Multiple Sequence Alignment Software Version 7: Improvements in Performance and Usability. *Molecular Biology and Evolution* 2013;**30**:772–80.

Köster J, Rahmann S. Snakemake—a scalable bioinformatics workflow engine. *Bioinformatics (Oxford, England)* 2012;**28**:2520–2.

Leray M, Yang JY, Meyer CP *et al.* A new versatile primer set targeting a short fragment of the mitochondrial COI region for metabarcoding metazoan diversity: application for characterizing coral reef fish gut contents. *Frontiers in Zoology* 2013;**10**:34.

Lozupone C, Lladser ME, Knights D *et al.* UniFrac: an effective distance metric for microbial community comparison. *The ISME Journal* 2010;**5**:169–72.

McKinney W. Data structures for statistical computing in Python. In. *Proceedings of the 9th Python in Science Conference*. Vol 445. 2010, 51–6.

Miya M, Sato Y, Fukunaga T *et al.* MiFish, a set of universal PCR primers for metabarcoding environmental DNA from fishes: detection of more than 230 subtropical marine species. *Royal Society Open Science* 2015;**2**:150088.

Oksanen J, Blanchet FG, Friendly M *et al.* Package "vegan": Community Ecology Package. 2020.

Parada AE, Needham DM, Fuhrman JA. Every base matters: assessing small subunit rRNA primers for marine microbiomes with mock communities, time series and global field samples. *Environmental Microbiology* 2016;**18**:1403–14.

Pargett DM, Birch JM, Preston CM *et al.* Development of a mobile ecogenomic sensor. *OCEANS 2015 - MTS/IEEE Washington* 2015:1–6.

Price MN, Dehal PS, Arkin AP. FastTree: Computing Large Minimum Evolution Trees with Profiles instead of a Distance Matrix. *Molecular Biology and Evolution* 2009;**26**:1641–50.

Prodan A, Tremaroli V, Brolin H *et al.* Comparing bioinformatic pipelines for microbial 16S rRNA amplicon sequencing. *PLOS ONE* 2020;**15**:e0227434.

Qalieh MW, Botvinnik O, O'Kane D *et al.* mwaskom/seaborn: v0.8.1 (September 2017). 2017, DOI: 10.5281/zenodo.883859.

Reiter T, Brooks† PT, Irber† L *et al.* Streamlining data-intensive biology with workflow systems. *GigaScience* 2021;**10**, DOI: 10.1093/gigascience/giaa140.

Rognes T, Flouri T, Nichols B *et al.* VSEARCH: a versatile open source tool for metagenomics. *PeerJ* 2016;**4**:e2584.

Ruppert KM, Kline RJ, Rahman MS. Past, present, and future perspectives of environmental DNA (eDNA) metabarcoding: A systematic review in methods, monitoring, and applications of global eDNA. *Global Ecology and Conservation* 2019;**17**:e00547.

Sievers F, Higgins DG. Clustal Omega, Accurate Alignment of Very Large Numbers of Sequences. In: Russell DJ (ed.). *Multiple Sequence Alignment Methods*. Vol 1079. 2014, 105–16.

Straub D, Blackwell N, Langarica-Fuentes A *et al.* Interpretations of Environmental Microbial Community Studies Are Biased by the Selected 16S rRNA (Gene) Amplicon Sequencing Pipeline. *Frontiers in Microbiology* 2020;**11**:550420.

Sunagawa S, Coelho LP, Chaffron S *et al.* Structure and function of the global ocean microbiome. *Science* 2015;**348**:1261359–9.

Thompson LR, Sanders JG, McDonald D *et al.* A communal catalogue reveals Earth's multiscale microbial diversity. *Nature* 2017;**551**:457–63.

Turnbaugh PJ, Ley RE, Mahowald MA *et al.* An obesity-associated gut microbiome with increased capacity for energy harvest. *Nature* 2006;**444**:1027–31.

Vargas C de, Audic S, Henry N *et al.* Eukaryotic plankton diversity in the sunlit ocean. *Science* 2015;**348**:1261605.

Vázquez-Baeza Y, Pirrung M, Gonzalez A *et al.* EMPeror: a tool for visualizing high-throughput microbial community data. *GigaScience* 2013;**2**:16.

Weißbecker C, Schnabel B, Heintz-Buschart A. Dadasnake, a Snakemake implementation of DADA2 to process amplicon sequencing data for microbial ecology. *GigaScience* 2020;**9**:giaa135.

Wickham H, Averick M, Bryan J *et al.* Welcome to the Tidyverse. *Journal of Open Source Software* 2019;**4**:1686.

Zafeiropoulos H, Viet HQ, Vasileiadou K *et al.* PEMA: a flexible Pipeline for Environmental DNA Metabarcoding Analysis of the 16S/18S ribosomal RNA, ITS, and COI marker genes. *GigaScience* 2020;**9**, DOI: 10.1093/gigascience/giaa022.

Zaiko A, Martinez JL, Schmidt-Petersen J *et al.* Metabarcoding approach for the ballast water surveillance – An advantageous solution or an awkward challenge? *Marine Pollution Bulletin* 2015;**92**:25–34.

Table S1. Parameters in the configuration file, *config.yaml*, that the user may edit as necessary. Additional parameters not shown may also be edited. The default configuration file is provided in the top level of the GitHub repository. The file format of *config.yaml*, YAML (yet another markup language), is a simple markup language that is used by Snakemake to specify parameters for a workflow.

Parameter	Description	Recommendation	Help
dada2pe_trunc_len_f<br>dada2pe_trunc_len_r<br>dada2se_trunc_len	Truncate bases (integer) from the 3' (right) ends of reads in DADA2.	Choose values that maximize length but remove low-quality ends. Note that DADA2 paired-end mode requires a minimum overlap of 12 bp to merge Read 1 and Read 2. See the section below "Sequence quality control and choice of truncation length" for instructions on using the included script fastqc_per_base_sequence_quality_dropoff.py.	dada2 denoise-paired; dada2 denoise-single
dada2pe_trim_left_f<br>dada2pe_trim_left_f<br>dada2se_trim_left	Trim bases (integer) from the 5' (left) ends of reads in DADA2.	Depending on your amplicon sequencing method, and if trimming was not done prior to running Tourmaline, you may have primer sequences, indexes, and/or adapters on the 5' ends of your reads. If so, set this parameter to remove those bases. If not, set this parameter to zero. Note that 5' trimming (this parameter) is done after 3' truncation (above parameter).	dada2 denoise-paired; dada2 denoise-single
deblur_trim_length	Truncate bases (integer) from the 3' (right) ends of reads in Deblur.	Choose values that maximize length but remove low-quality ends. See the section below "Sequence quality control and choice of truncation length" for instructions on using the included script fastqc_per_base_sequence_quality_dropoff.py.	deblur denoise-other
dada2pe_pooling_method<br>dada2se_pooling_method	DADA2 pooling method.	Choose pseudo for pseudo-pooling or independent for no pooling.	dada2 denoise-paired; dada2 denoise-single
dada2pe_chimera_method<br>dada2se_chimera_method	DADA2 chimera method.	Choose pooled if pseudo-pooling otherwise consensus or none.	dada2 denoise-paired; dada2 denoise-single
alignment_method	Multiple sequence alignment method.	Choose muscle or clustalo for best accuracy or mafft for faster results.	muscle; clustalo; mafft
classify_method	Taxonomic classification method.	Choose naive-bayes for best accuracy or consensus-blast for faster results.	feature-classifier
exclude_terms	Filter terms (taxa) from taxonomy.	Specify terms (comma-separated, no spaces) to find in taxonomy and filter out (case-insensitive), or provide a nonsense term to skip this step when filtering.	taxa filter-seqs
repseq_min_length<br>repseq_max_length	Set minimum and maximum sequence lengths to filter representative sequences by.	Limits are inclusive, i.e., sequences will be retained if greater than or equal to minimum, less than or equal to maximum. Leave defaults (0, 10000) to do no filtering.	taxa filter-seqs
repseq_min_abundance<br>repseq_min_prevalence	set minimum abundance and prevalence limits to filter representative sequences by.	Limit is inclusive, i.e., sequences will be retained if greater than or equal to minimum. Leave default (0) to do no filtering.	taxa filter-seqs
odseq_distance_metric	Distance metric for odseq.	Choose metric from: linear, affine.	odseq
odseq_bootstrap_replicates	Number (integer) of bootstrap replicates for odseq.	Choose more replicates for more robust detection of outliers, fewer replicates for faster processing.	odseq
odseq_threshold	Threshold (float) for bootstrap probability distribution for odseq.	Probability to be at the right of the bootstrap scores distribution when computing outliers. Tune this parameter depending on the diversity and occurrence of outliers in the MSA.	odseq
core_sampling_depth	Rarefaction depth (integer) for core diversity metrics.	Choose a value that balances sequencing depth (more is better) with number of samples retained (more is better).	diversity core-metrics-phylogenetic
alpha_max_depth	Rarefaction depth (integer) for alpha rarefaction.	Choose a value that balances sequencing depth (more is better) with number of samples retained (more is better).	diversity alpha-rarefaction
beta_group_column	Column (text) in your metadata to test beta-diversity group significance.	Choose a category that may differentiate your samples. This analysis can be rerun with different columns by renaming the output file and changing the value in config.yaml before running again.	diversity beta-group-significance
report_theme	HTML report theme.	Choose from: github, gothic, newsprint, night, pixyll, whitey.	Typora theme gallery

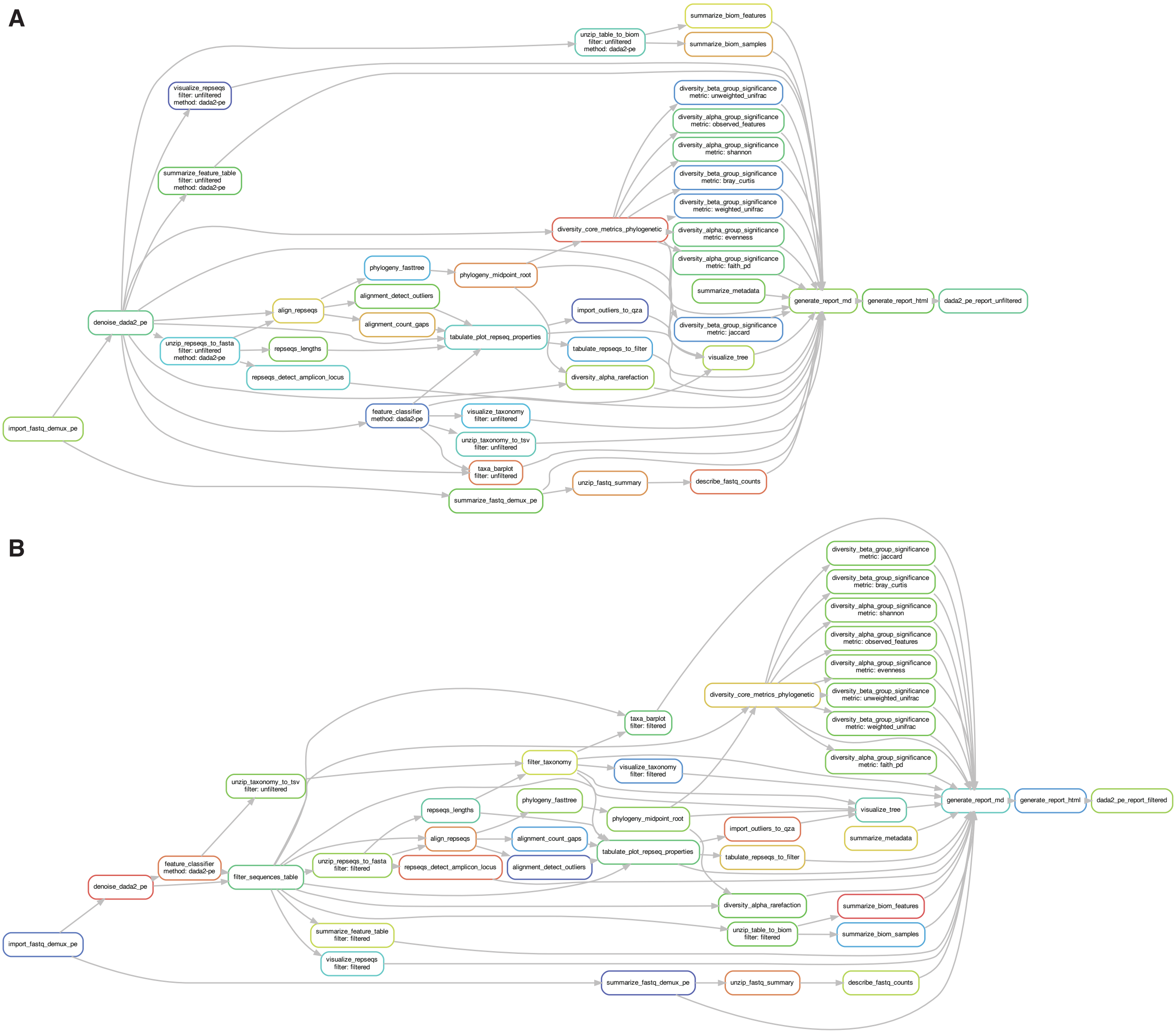

Figure S1. Directed acyclic graphs (DAGs) of the Tourmaline workflow for the DADA2 paired-end method from start to report in (**A**) unfiltered mode and (**B**) filtered mode. This figure was generated from the test data that comes with the repository by running the commands (**A**) *snakemake dada2_pe_report_unfiltered --dag | dot -Tpdf -Grankdir=LR -Gnodesep=0.1 -Granksep=0.1 dag_pe_report_unfiltered.pdf* and (**B**) *snakemake dada2_pe_report_filtered --dag | dot -Tpdf -Grankdir=LR -Gnodesep=0.1 -Granksep=0.1 dag_pe_report_filtered.pdf*. For a simpler graph, substitute *--rulegraph* for *--dag* in the above commands.

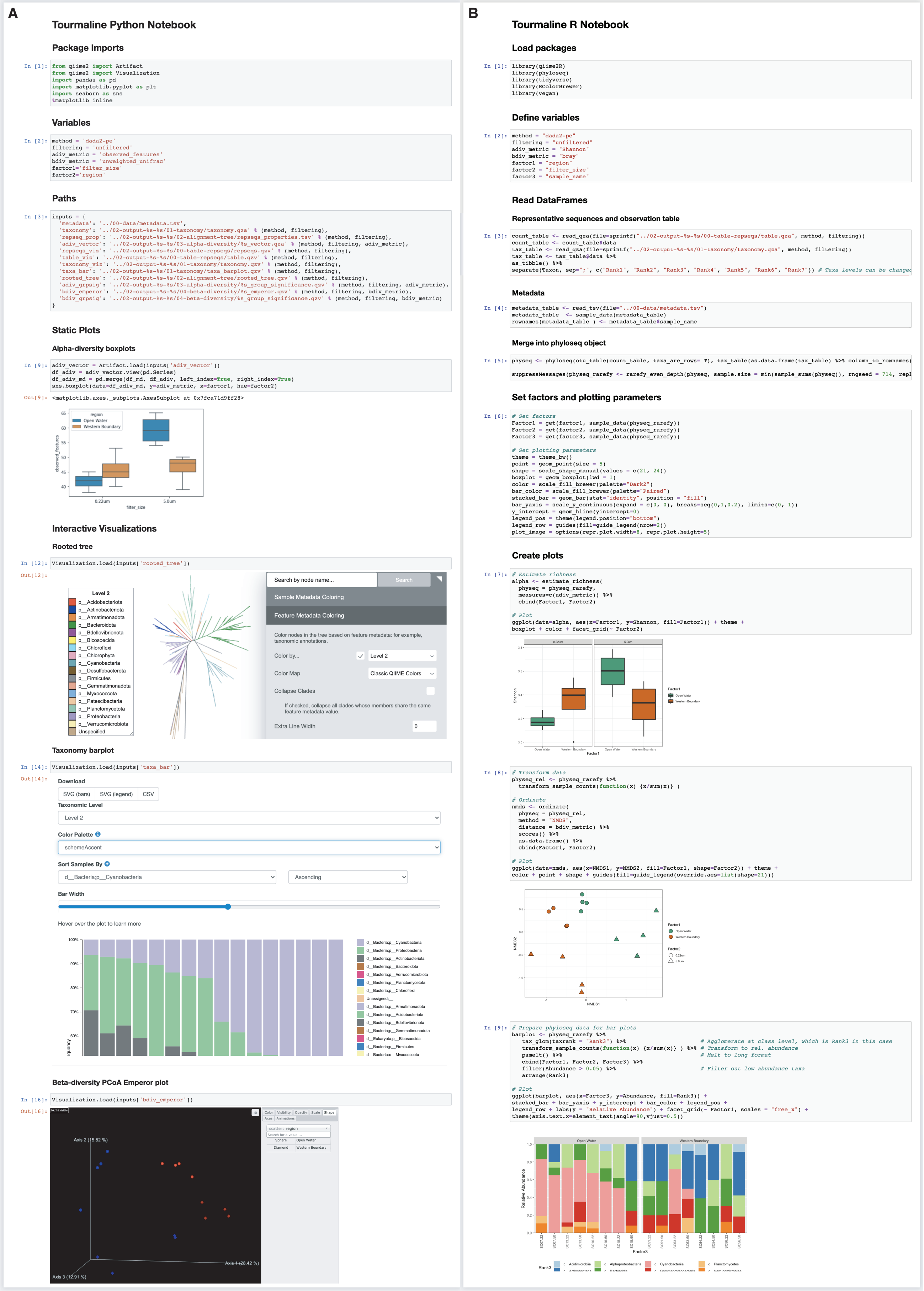

Figure S2. Screenshots of Tourmaline's included Python and R Jupyter notebooks running the provided test data. Both notebooks are designed to run out-of-the-box with the Tourmaline output from any dataset. (**A**) The Tourmaline Python notebook loads and displays sample metadata, feature metadata (representative sequences properties and taxonomy), static plots generated by Seaborn, and interactive QIIME 2 visualizations. (**B**) The Tourmaline R notebook demonstrates how to load .qza files (counts and taxonomy) into R, merge files with metadata into a single phyloseq object, and generate high-quality visualizations of community diversity and taxonomy using phyloseq and suite of tidyverse packages (e.g., ggplot2).
