# Supplementary material for: Tourmaline: A containerized workflow for rapid and iterable amplicon sequence analysis using QIIME 2 and Snakemake
Source: Gigascience. 2022 Jul 28;11:giac066. doi: 10.1093/gigascience/giac066 (PMC9334028; doi:10.1093/gigascience/giac066)
Supplement: giac066_GIGA-D-21-00281_Revision_1 [file giac066_giga-d-21-00281_revision_1.pdf]

GigaScience

Tourmaline: a containerized workflow for rapid and iterable amplicon sequence analysis using QIIME 2 and Snakemake

--Manuscript Draft--

**Manuscript Number:**	GIGA-D-21-00281R1
**Full Title:**	Tourmaline: a containerized workflow for rapid and iterable amplicon sequence analysis using QIIME 2 and Snakemake
**Article Type:**	Technical Note
**Funding Information:**	National Oceanic and Atmospheric Administration (NA06OAR4320264 06111039) — Dr. Luke R. Thompson<br>National Oceanic and Atmospheric Administration (NA17OAR4320152) — Not applicable<br>National Oceanic and Atmospheric Administration — Mr. Grant Sanderson, Dr. Kelly D. Goodwin
**Abstract:**	Background: Amplicon sequencing (metabarcoding) is a common method to survey diversity of environmental communities whereby a single genetic locus is amplified and sequenced from the DNA of whole or partial organisms, organismal traces (e.g., skin, mucus, feces), or microbes in an environmental sample. Several software packages exist for analyzing amplicon data, among which QIIME 2 has emerged as a popular option because of its broad functionality, plugin architecture, provenance tracking, and interactive visualizations. However, each new analysis requires the user to keep track of input and output file names, parameters, and commands; this lack of automation and standardization is inefficient and creates barriers to meta-analysis and sharing of results. Findings: We developed Tourmaline, a Python-based workflow that implements QIIME 2 and is built using the Snakemake workflow management system. Starting from a configuration file that defines parameters and input files—a reference database, a sample metadata file, and a manifest or archive of FASTQ sequences—it uses QIIME 2 to run either the DADA2 or Deblur denoising algorithm, assigns taxonomy to the resulting representative sequences, performs analyses of taxonomic, alpha, and beta diversity, and generates an HTML report summarizing and linking to the output files. Features include support for multiple cores, automatic determination of trimming parameters using quality scores, representative sequence filtering (taxonomy, length, abundance, prevalence, or ID), support for multiple taxonomic classification and sequence alignment methods, outlier detection, and automated initialization of a new analysis using previous settings. The workflow runs natively on Linux and macOS or via a Docker container. We ran Tourmaline on a 16S rRNA amplicon dataset from Lake Erie surface water, showing its utility for parameter optimization and the ability to easily view interactive visualizations through the HTML report, QIIME 2 viewer, and R- and Python-based Jupyter notebooks. Conclusions: Automated workflows like Tourmaline enable rapid analysis of environmental and biomedical amplicon data, decreasing the time from data generation to actionable results. Tourmaline is available for download at github.com/aomlomics/tourmaline.
**Corresponding Author:**	Luke R. Thompson, Ph.D.<br>NOAA Atlantic Oceanographic and Meteorological Laboratory<br>Miami, Florida UNITED STATES
**Corresponding Author Secondary Information:**	
**Corresponding Author's Institution:**	NOAA Atlantic Oceanographic and Meteorological Laboratory
**Corresponding Author's Secondary Institution:**	
**First Author:**	Luke R. Thompson, Ph.D.
**First Author Secondary Information:**	

Powered by Editorial Manager® and ProduXion Manager® from Aries Systems Corporation

Order of Authors:	Luke R. Thompson, Ph.D.
	Sean R Anderson, Ph.D.
	Paul A. DenUyl
	Nastassia V. Patin, Ph.D.
	Shen Jean Lim
	Grant Sanderson
	Kelly D. Goodwin, Ph.D.
Order of Authors Secondary Information:	

Response to Reviewers:

Point-by-point Response

Reviewer #1

Thompson et al. present a workflow for amplicon sequencing analysis, wrapping commands from the commonly used QIIME2 package in the commonly used workflow manager Snakemake. The manuscript is clearly structured and contains figures of appropriate quality. Building and testing user-friendly workflows that facilitate the use of existing software is an important task for the research community. The chosen existing softwares (namely Snakemake as workflow manager and QIIME2's calls to DADA2 and deblur, as well as QIIME2's visualisations) are trusted and often used by the community. Next to manuscript, I have inspected the GitHub/wiki page of the proposed workflow and tested it on the provided test data as well as an independent data set. I have run into some issues, which I will put forward below, together with some comments on the content of and omissions from the manuscript.

We thank the reviewer for the comprehensive evaluation of our workflow and accompanying manuscript. Their comments have been very helpful in improving the tool and revising the manuscript, as we describe below in our response.

Manuscript:

1. Overall, the reads more like a manual or tutorial than a methods description. The point-by-point description of the outputs may be a bit lengthy.

> We thank the reviewer for this candid critique and have condensed the
> instructions and description of outputs and referred the reader to the
> GitHub Wiki for more detail.

2. The manuscript is missing information on runtimes and hardware requirements. This is in particular a pity because the workflow does not make use of parallelisation of the called tools. It might be pretty slow on large datasets?

> We did not highlight in the original manuscript that the workflow does
> indeed support parallelization of individual commands using the
> Snakefile. We have now added a section on benchmarking and
> parallelization, including runtimes for a medium-sized dataset with
> and without parallelization. The runtime for a typical dataset (96
> samples with ~120,000 reads per sample) was 5 hours with a single
> core but decreased to 2 hours with 8 cores.

3. There is also no justification for the choices of the analyses that are done and the defaults that were chosen.

> We thank the reviewer for pointing out the lack of justification for
> the choices made regarding commands and default parameters. We have

> added justification for these choices, noting that the commands chosen
> encompass analyses commonly used by microbiome and eDNA researchers,
> and that parameters were chosen to balance run performance and
> accuracy and to work with the test data.

4. In the introduction, other published amplicon sequencing workflows are cited and dismissed as not all well documented. Other than maybe not being so well documented, there are differences in scope between these workflows and the one described here. It would be very helpful for readers to be informed on how the described workflow is set apart from those workflows. And also from QIIME2 (all of the images in figure 3, for example, are QIIME2's visualisation work and not part of the workflow's report).
Finally, tagseq, which also wraps QIIME2 commands in snakemake, is not mentioned. From my point of view, the workflow still requires the user to be able to do quite a lot of data setup in the command line environment and requires knowledge of QIIME2, while resolving relatively little by wrapping the commands in Snakemake (also see my next point). Clearly, it would be helpful to discuss what existing problem the workflow overcomes that the others (and QIIME2) don't.

> We acknowledge that we were unfairly dismissive of other workflows in
> the original manuscript. We have now included a more comprehensive
> list of other workflows in the introduction that might be used instead
> of Tourmaline, including tagseq, CoMA, and ASAP 2. We have also
> highlighted throughout the manuscript what Tourmaline provides that
> makes it unique and potentially a good option for some users,
> specifically: 1- the additional summary plots and reports (which are
> now highlighted rather than the QIIME 2 visualizations), 2- the Docker
> container containing all the dependencies, 3- Snakemake features
> (single config file, reproducible directory structure, and on-demand
> commands), 4- Jupyter notebooks ready to load and analyze Tourmaline
> outputs, and 5- a well-documented wiki with step-by-step instructions.

5. In the same paragraph of the introduction, it is mentioned that the workflow might evolve with QIIME2. However, it makes use of only a small part of QIIME's options/commands - is there a plan to widen the scope? How will continuous support be done? Is there a plan to integrate the workflow into the QIIME software ecosystem?
Similarly, the workflow is not using Snakemake to its full potential, e.g. it does require several manual installation steps instead of making use of Snakemake's conda integration; it doesn't make use of Snakemake's reporting ability, which might be interesting together with QIIME2's data provenance. So, is there a plan to improve this? Also for developing it towards better usability on (cloud) cluster structures?

> In its current implementation, Tourmaline provides a core workflow
> with many of the most popular commands provided as rules (see answer
> above to your point #3). We continue to add more QIIME 2 commands
> since the original submission, such as DEICODE (Martino et al. 2019),
> which is a tool that performs Aitchison transformation of amplicon
> data and allows for users to visualize community composition of
> inherently sparse datasets, and is now part of the Tourmaline
> workflow. We expect to continue adding the most useful commands. We
> have shared Tourmaline in the QIIME 2 Forum and have received positive
> feedback from the QIIME 2 user community, and we hope that, with the
> publication of this manuscript, this community will contribute to the
> workflow, possibly with new forks that implement more commands.
> Notably, we have received funding to deploy Tourmaline on the Amazon
> cloud. To better explain our vision for Tourmaline, we have closed the
> conclusion thusly: "Future improvements to the workflow will include
> support for new QIIME 2 releases and plugins, better integration with

> Snakemake, possibly including Conda integration and connecting
> Snakemake's reporting ability with QIIME 2's provenance tracking, and
> enhanced support for cloud computing environments. With its existing
> features that balance usability, functionality, iterability, and
> scalability, and with continued development with support from the
> research community, Tourmaline will be a valuable and longstanding
> tool for amplicon sequence analysis."

Testruns:

What worked:

- Overall, I could install the software on a linux machine by following the description on the GitHub webpage. The test run worked as expected. The data was accessible and I could visualise it using the QIIME2 online viewer. I could run the workflow on an unrelated dataset.

> We are glad that you could successfully install the software, run the
> test data, and visualize the outputs. It is also good to hear you were
> successful applying Tourmaline to an unrelated dataset.

What could be improved:

- The setup of the input was a bit annoying, because the names and paths to the inputs and outputs need to be set at various places. The fact that existing and non-existing inputs have to be defined in the config confused me at first. The error messages that ensued from not doing this right were uninformative (these cases could be caught by the Snakefile with or without the help of a scheme).

> We have simplified the input files by requiring that they be named (or
> have symbolic links) to match the defaults. Therefore the user does
> not need to set these parameters, and the workflow will automatically
> generate the inputs/outputs as required. We have added a rule that
> checks, at the beginning of the workflow, if the required input files
> (and which version of them) are provided.

- While the workflow is very well documented, the settings for the individual denoising / taxonomy steps are not. The links to the QIIME2 documentation don't point to the current version.

> We have added additional guidance in the config file, including help
> commands and links that describe the underlying commands being run,
> especially for the denoising, taxonomy, alignment, and filtering
> steps. It is normal behavior for the links to the QIIME 2
> documentation to point to an older version of the documentation,
> because we are using an older version of QIIME 2 (due to dependencies
> that do not support the latest versions of QIIME 2 and its use of
> Python 3.8).

What didn't work:

- Running a small dataset - the workflow expects to be able to do statistical test with groups and replicates. However, only a late step checks if the data set is suitable, so there is a failure after considerable running time, which is annoying. While this kind of analysis may be the most common application, it's not the only one. It would be good if those parts of the workflow that require certain dataset structures could be switched off.

> We thank the reviewer for pointing out this snag in the workflow.
> There is a parameter in config.yaml called "beta_group_column" and by
> default it is set to "region". This should work with the test data but

Powered by Editorial Manager® and ProduXion Manager® from Aries Systems Corporation

> will fail with another dataset unless it happens to contain a metadata
> column called "region". This parameter is not required for initial
> steps, only when the diversity rule is run, and if it fails and the
> rule has to be re-run, only the commands that need to be rerun will be
> run. We have added a note to config.yaml to notify the user that "this
> column must appear in the metadata file (eg, 00-data/metadata.tsv)".
> We have also added a rule that checks, at the beginning of the
> workflow, if this parameter is in the metadata file and will throw an
> error if not.

minor:

- As a very irregular user of QIIME2, I find the QIIME2-jargon difficult to understand (e.g. artefacts and artefact equivalents, manifesto, and the QIIME2 names of the DADA2 and deblur steps, emperor plot...). It would be better, if these were defined (and maybe not all discussed in detail).

> We have avoided using jargon wherever possible while still using the
> appropriate terminology. We have described the steps in more detail in
> the wiki. As this is a QIIME 2-based workflow, some knowledge of QIIME
> 2 jargon and data structures is unavoidable.

- Personally, I would like to have a primer removal step in the workflow. But that's a design decision that can be discussed.

> We agree that primer removal is important to consider for any amplicon
> workflow. We did not include primer removal or quality filtering
> because these can be an iterative process and will vary across
> projects and applications. It can be dangerous to have a single
> removal step, without user modification. We have included text in the wiki
> (https://github.com/aomlomics/tourmaline/wiki/3-Setup#remove-primers-if-necessary)
> with code and steps to remove primers in QIIME 2 using the cutadapt
> plugin. Users can control primer removal settings and cutadapt can be
> performed in the same QIIME 2 environment. Trimmed reads are then
> easily passed along to Tourmaline.

- "the fungal internal transcribed spacer (ITS) of the rRNA gene (Abarenkov et al. 2010)" - the internal transcribed spacer is not within an rRNA gene, but the different ITS regions are found between rRNA genes.

> We have clarified this distinction in the introduction.

Reviewer #2

Dear authors,

First of all, my apologies for the delay in the submission of the review.
I appreciated the opportunity to review your manuscript. Tourmaline aims at facilitating an easy-to-follow architecture for tracking input and output file names, parameters, and commands of QIIME2 runs to enhance meta-analyses. If I am not mistaken, this is the corner-stone of this study so my review is based on that.

Running Tourmaline is straightforward and its documentation is exceptional. The video tutorial and the GitHub wiki (https://github.com/aomlomics/tourmaline/wiki) allows non-experienced users to start working their analysis and the containerized version of the tool allows an easy-to-go installation in multiple operating systems without extra effort. The extra visual components provide insight in a nice way and the report returned can provide added value on the runs.

However, even if I do share the authors' interest on usability and
interoperability and tools could have a great impact in the community
indeed, Tourmaline currently lacks any substantial features to be
considered as a stand-alone software tool. In addition, there are
several issues that it is my belief that need to be addressed (see the
following list).

> We thank the reviewer for the thorough review. In our revisions we have
> highlighted the features of Tourmaline that set it apart from other
> amplicon workflows. We believe that it will be a useful tool for a
> certain group of users. Features that make Tourmaline unique include: 1-
> the additional summary plots and reports (which are now highlighted
> rather than the QIIME 2 visualizations), 2- the Docker container
> containing all the dependencies, 3- Snakemake features (single config
> file, reproducible directory structure, and on-demand commands), 4-
> Jupyter notebooks ready to load and analyze Tourmaline outputs, and 5- a
> well-documented wiki with step-by-step instructions.

Major issues

Major Issue #1: The authors claim that "this lack of automation and
standardization [in tracking input and output file names, parameters,
and commands on QIIME2] is inefficient and creates barriers to
meta-analysis and sharing of results. Therefore, what Tourmaline and
thus, the manuscript needs to demonstrate, is that meta-analyses are now
feasible to a greater extent, thanks to the Tourmaline wrapper.

> We thank the reviewer for providing the opportunity to better highlight
> the capacity of Tourmaline to facilitate meta-analysis. We have now
> provided a section on how this works in practice and have added a new
> Jupyter notebook that walks through merging two sets of Tourmaline
> outputs and performing some basic diversity analyses on the merged
> output.

Major Issue #2: Assuming that enhancing meta-analyses is the main
contribution of Tourmaline, it is fundamental to consider the minimum
information about a marker gene sequence (MIMARKS) standard of the
Genomic Standards Consortium (GSC). Rather than just mentioning MIMARKS,
Tourmaline needs to explore ways to exploit such standards, i.e. perhaps
by adding MIMARKS columns in the config file.

> We agree that including support for MIxS/MIMARKS is important as we move
> as a community toward more standardization. As suggested, we have added
> guidance to the config file about using MIxS/MIMARKS metadata fields and
> have made the metadata that comes with Tourmaline fully
> MIMARKS-compliant.

Major Issue #3: As QIIME2 has been developed on the basis of a plugin
architecture, it would be highly recommended that such an application
could be provided as a plugin too, joining the corresponding QIIME2
library (https://library.qiime2.org/plugins/).

> We have reached out to the QIIME 2 community via the QIIME 2 Forum, and
> have received positive feedback. Our hope is that this community will
> consider contributing to the workflow codebase, for example by
> submitting pull requests, especially after the manuscript is published.
> A workflow such as this one would, however, not be appropriate to be a
> plugin, as plugins function as QIIME 2 commands, whereas this workflow
> wraps many commands. We certainly plan to continue integrating new
> plugins/commands into Tourmaline as they are added to QIIME 2.

Major Issue #4: With respect to the structure of the manuscript, it is
my belief that there are sections that should be omitted. Tutorials and
"how to" are of extremely valuable but it would be better to be

provided either as supplementary material or through repositories, e.g. GitHub wiki, GitHub pages etc, rather than in the main manuscript. The wiki page on Tourmaline's GitHub repository is rather informative. An alternative might be merging the "Overview" along with the "Snakefile", "Config file", "Input files" and "Run the workflow" sections, to describe "The Tourmaline workflow" architecture in a less verbose way, highlighting the role of the "Snakefile" and the "config.yml" files and the architecture that binds them together. "Documentation", "Installation", "Cloning" subsections could/should be omitted too.

> We agree and thank the reviewer for this suggested restructuring. We
> have condensed the instructions and description of outputs and referred
> the reader to the GitHub Wiki for more detail.

Major Issue #5: The test dataset does not allow the validation of Tourmaline in meta-analyses. It is rather important to have a testbed dataset to demonstrate "how to run" but a use case of an actual meta-analysis is required to demonstrate how different analyses can be combined in the framework of Tourmaline and provide further insight than those of the initial ones.

> This is a great suggestion, and we have followed this advice. We have
> added a Jupyter notebook that performs merging of two (or more) sets of
> Tourmaline outputs and described how this works in the manuscript.

Major Issue #7: No license has been included in the "Availability of supporting source code" section. On Tourmaline GitHub repo a license (https://github.com/aomlomics/tourmaline#license) is mentioned, yet GigaScience asks for an appropriate Open Source Initiative compliant license (https://opensource.org/licenses/category). In addition, I tried to find if the QIIME2 license is mentioned in a Tourmaline Docker container and I could not; if I am not mistaken that is required based on the QIIME2 license (https://github.com/qiime2/qiime2/blob/master/LICENSE). My apologies again in case of any misapprehension.

> We have updated the license on GitHub to a 3-clause BSD license. As
> recommended by the QIIME 2 lead developer, we have added text to the
> DockerHub page for the Tourmaline Docker image stating, "This docker
> image is based on a QIIME 2 Docker image hosted at
> https://quay.io/repository/qiime2/core?tag=2021.2. The QIIME 2 license
> can be found at https://github.com/qiime2/qiime2/blob/master/LICENSE."
> We have added both of these pieces of information to the section
> "Availability of supporting source code".

Major Issue #8: Parameter optimization is indeed one of the greatest challenges in metabarcoding bioinformatics analyses. However, it is not clear to me how by keeping the exact same names in your output files, will you be able to compare the results of the different runs.

> This can be done by creating copies of the entire Tourmaline directory
> using the helper script. Then one or more parameters can be changed in
> the copies to see how these changes affect the results. We have made
> this clearer in the revised manuscript and added a section on parameter
> optimization.

Major Issue #9: I realise that the authors provide Figures 2 and 3 in a complementary way, presenting the visual component returned after each step. However, having a figure with 16 screenshots makes it hard for the reader to realize what is coming from QIIME2 and what from Tourmaline but most importantly does not highlight the added value that Tourmaline provides to such an analysis. It is my belief that Figure 2 could remain as it, while Figure 3 should focus on the output components that are not

provided by QIIME2 routines, but from Tourmaline functionalities. In case of a meta-analysis, this figure should highlight all the added value that using QIIME2 through the Tourmaline wrapper would provide.

> We agree these figures were too busy and have simplified them. We thank
> the reviewer for the suggestion to focus on the parts of the workflow
> that are specific to Tourmaline and have revised the figure accordingly.

Minor issues

Minor Issue #1: Please rephrase the Findings section in the abstract, so that it is clear that Tourmaline invokes QIIME2 routines to implement taxonomy assignment, perform analysis etc. It is required to state clearly what QIIME2 does and what are the extra features of Tourmaline throughout the manuscript.

> We have rephrased the abstract to make clearer the relationship between
> Tourmaline and QIIME 2, and have striven to make this distinction clear
> throughout the manuscript. Notably, Fig. 3 is now focused on outputs
> unique to the Tourmaline workflow.

Minor Issue #2: The conclusion you mention in the abstract is not in line with the scope of Tourmaline that was described earlier. Tourmaline does not accelerate the performance of QIIME2 routines. Its aim, as mentioned earlier, is to enhance meta-analysis and sharing of results.

> We believe that this wording is appropriate for a couple of reasons.
> First, Tourmaline does provide speed improvements by supporting parallel
> processing. More importantly, Tourmaline speeds up the process of
> running an analysis by streamlining setting up commands, inputs, and
> output---which is where we spend most of our time in bioinformatics.
> Once the user has mastered the workflow, analysis of a dataset is rapid
> because so many of the tedious tasks of managing the commands, inputs,
> and outputs are taken care of.

Minor Issue #3: terms such as "meta-analysis", "reproducibility", "metadata" could be added.

> We have added some of these terms as keywords and made sure to reference
> these concepts throughout the manuscript.

Minor Issue #4: In line Information gained... resource management it would be nice to add references for the value of the method in each of the various fields mentioned.

> We have added references to Halfvarson et al. (2017) and Thomson and
> Willerslev (2015) to the background section that describe how
> metabarcoding information informs microbiome research and ecosystem
> conservation/management.

Minor Issue #5: Usually, (shotgun) metagenome analyses are used to measure diversity in microbiomes, meaning the functional, genomic diversity; the term microbiome has been widely used as the collection of genomes from all microbial taxa present in a sample. It would be better to rephrase this like "popular method of measuring taxonomic/microbial diversity of host microbiome or in environmental samples"

> We have clarified this in the background section.

Minor Issue #6: As an overall comment, long sentences make the manuscript hard to read. In this case: "PCR primers have been used to generate amplicons of the bacterial 16S rRNA gene in studies of human and animal microbiota [..] among others." should be splitted.

Powered by Editorial Manager® and ProduXion Manager® from Aries Systems Corporation

	> We have modified this sentence (and other long sentences) to improve<br>> readability throughout the manuscript.<br><br>Minor Issue #7: "other environmental surveys" please explain.<br><br>> We have removed the term "other environmental surveys".<br><br>Minor Issue #8: It is not clear to me how the study of Prodan et al.<br>(2020) is related to the standardization of amplicon data analysis.<br><br>> We agree and have removed this reference.<br><br>Minor Issue #9: The authors highlight that the standard directory<br>structure enhances data exploration and parameter optimization. A use<br>case to demonstrate this main feature of Tourmaline would be of high<br>value.<br><br>> We thank the reviewer for this suggestion and have added a example use<br>> case in the section "Helper scripts & parameter optimization". We have<br>> also noted how the Jupyter notebooks take advantage of Tourmaline's<br>> defined directory structure.<br><br>Minor Issue #10: The purpose of performing amplicon sequencing or<br>metabarcoding is to reveal patterns of diversity in biological systems.<br>That is not the only case, please rephrase.<br><br>> We have amended this sentence to include other outcomes of amplicon<br>> sequencing.
**Additional Information:**	
**Question**	**Response**
Are you submitting this manuscript to a special series or article collection?	No
**Experimental design and statistics**<br><br>Full details of the experimental design and statistical methods used should be given in the Methods section, as detailed in our Minimum Standards Reporting Checklist. Information essential to interpreting the data presented should be made available in the figure legends.<br><br>Have you included all the information requested in your manuscript?	Yes
**Resources**<br><br>A description of all resources used, including antibodies, cell lines, animals and software tools, with enough information to allow them to be uniquely identified, should be included in the	Yes

Methods section. Authors are strongly encouraged to cite Research Resource Identifiers (RRIDs) for antibodies, model organisms and tools, where possible.<br><br>Have you included the information requested as detailed in our Minimum Standards Reporting Checklist?	
**Availability of data and materials**<br><br>All datasets and code on which the conclusions of the paper rely must be either included in your submission or deposited in publicly available repositories (where available and ethically appropriate), referencing such data using a unique identifier in the references and in the “Availability of Data and Materials” section of your manuscript.<br><br>Have you have met the above requirement as detailed in our Minimum Standards Reporting Checklist?	Yes

Click here to access/download;Manuscript;tourmaline_manuscript_2022022

GigaScience, 2022, 1–17

doi: xx.xxxx/xxxx
Manuscript in Preparation
Technical Note

TECHNICAL NOTE

Tourmaline: a containerized workflow for rapid and iterable amplicon sequence analysis using QIIME 2 and Snakemake

Luke R. Thompson[1,2,*], Sean R. Anderson[1,2], Paul A. Den Uyl[3], Nastassia V. Patin[2,4,†], Shen Jean Lim[2,4], Grant Sanderson[5] and Kelly D. Goodwin[2,†]

[1]Northern Gulf Institute, Mississippi State University, Mississippi State, MS, USA and [2]Ocean Chemistry and Ecosystems Division, Atlantic Oceanographic and Meteorological Laboratory, National Oceanic and Atmospheric Administration, Miami, Florida, USA and [3]Cooperative Institute for Great Lakes Research, University of Michigan, Ann Arbor, MI, USA and [4]Cooperative Institute for Marine and Atmospheric Studies, Rosenstiel School of Marine and Atmospheric Science, University of Miami, Miami, FL, USA and [5]Marine Science Department, University of Hawaii, Hilo, HI, USA

[*]Correspondence: luke.thompson@noaa.gov
[†]Stationed at Southwest Fisheries Science Center, National Marine Fisheries Service, National Oceanic and Atmospheric Administration, La Jolla, CA, USA

Abstract

Background: Amplicon sequencing (metabarcoding) is a common method to survey diversity of environmental communities whereby a single genetic locus is amplified and sequenced from the DNA of whole or partial organisms, organismal traces (e.g., skin, mucus, feces), or microbes in an environmental sample. Several software packages exist for analyzing amplicon data, among which QIIME 2 has emerged as a popular option because of its broad functionality, plugin architecture, provenance tracking, and interactive visualizations. However, each new analysis requires the user to keep track of input and output file names, parameters, and commands; this lack of automation and standardization is inefficient and creates barriers to meta-analysis and sharing of results. **Findings:** We developed Tourmaline, a Python-based workflow that implements QIIME 2 and is built using the Snakemake workflow management system. Starting from a configuration file that defines parameters and input files—a reference database, a sample metadata file, and a manifest or archive of FASTQ sequences—it uses QIIME 2 to run either the DADA2 or Deblur denoising algorithm, assigns taxonomy to the resulting representative sequences, performs analyses of taxonomic, alpha, and beta diversity, and generates an HTML report summarizing and linking to the output files. Features include support for multiple cores, automatic determination of trimming parameters using quality scores, representative sequence filtering (taxonomy, length, abundance, prevalence, or ID), support for multiple taxonomic classification and sequence alignment methods, outlier detection, and automated initialization of a new analysis using previous settings. The workflow runs natively on Linux and macOS or via a Docker container. We ran Tourmaline on a 16S rRNA amplicon dataset from Lake Erie surface water, showing its utility for parameter optimization and the ability to easily view interactive visualizations through the HTML report, QIIME 2 viewer, and R- and Python-based Jupyter notebooks. **Conclusions:** Automated workflows like Tourmaline enable rapid analysis of environmental and biomedical amplicon data, decreasing the time from data generation to actionable results. Tourmaline is available for download at github.com/aomlomics/tourmaline.

Key words: amplicon sequencing; metabarcoding; environmental DNA; eDNA; microbiome; meta-analysis

Background

Earth's environments are teeming with environmental DNA (eDNA): free and cellular genetic material from whole microorganisms (Consortium 2012; Thompson *et al.* 2017) or remnants of larger macroorganisms (Deiner *et al.* 2017; Compson *et al.* 2020). This eDNA can be collected, extracted, and sequenced to reveal the identities and functions of the organisms that produced it. Amplicon sequencing (metabarcoding), whereby a short genomic region is amplified and sequenced using polymerase chain reaction (PCR) from an environmental or experimental community's eDNA, is a popular method for measuring taxonomic diversity of microbiomes and environmental samples (Zaiko *et al.* 2015; Deiner *et al.* 2017; Ruppert, Kline and Rahman 2019). PCR primers have been used to generate amplicons of the bacterial 16S rRNA gene in studies of human and animal-associated microbiota (Turnbaugh *et al.* 2006; Ahn *et al.* 2013; Kartzinel *et al.* 2019), as well as environmental microbiota (Sunagawa *et al.* 2015; Thompson *et al.* 2017). Other regions that are commonly targeted include the fungal internal transcribed spacer (ITS) regions between rRNA genes (Abarenkov *et al.* 2010), the 18S rRNA gene of eukaryotes (Vargas *et al.* 2015), the mitochondrial cytochrome oxidase I (COI) gene of invertebrate and vertebrate eDNA (Leray *et al.* 2013), and the mitochondrial 12S rRNA gene of fish (Miya *et al.* 2015). Information gained from amplicon metabarcoding has far reaching implications for human health (e.g., microbiome research), ecosystem function and conservation, and resource management (Thomsen and Willerslev 2015; Halfvarson *et al.* 2017a).

Combining datasets from amplicon surveys and performing meta-analysis can reveal patterns impossible to observe from individual studies and also provide more power in statistical analyses. One example of the power of meta-analysis in amplicon surveys is the Earth Microbiome Project (Thompson *et al.* 2017), which used a single 16S rRNA amplicon method, metadata standard, and denoising algorithm to sequence and analyze over 25,000 microbial communities from around Earth. Standardization of methods is critical for comparability across studies. A lack of standardized methods is one of the main factors limiting cross-comparison of microbiome or eDNA datasets in meta-analyses (Dickie *et al.* 2018; Harper *et al.* 2019). While standardizing detailed laboratory methods across many labs in multiple countries is a major challenge, standardizing analysis methods and metadata formats is much more feasible.

A popular approach for standardizing amplicon data analysis is to develop analysis pipelines or workflows (Reiter *et al.* 2021) to run on local or networked computing resources or in the cloud. Amplicon workflows have become increasingly popular and include pipelines like Anacapa (Curd *et al.* 2019), Banzai (https://github.com/jimmyodonnell/banzai), PEMA (Zafeiropoulos *et al.* 2020), Ampliseq (Straub *et al.* 2020), Cascabel (Asbun *et al.* 2019), dadasnake (Weißbecker, Schnabel and Heintz-Buschart 2020), CoMA (Hupfauf *et al.* 2020), ASAP 2 (Tian and Imanian 2022), and tagseq (https://github.com/shu251/tagseq-qiime2-snakemake). However, stand-alone pipelines typically do not have access to a wide range of datasets, and many pipelines are bespoke workflows with a jumble of custom scripts for the user to navigate. A variation on this model would be a stand-alone workflow that runs on top of a widely used amplicon software tool, such that it could take advantage of the functionality of the underlying tool and evolve with it, while remaining interoperable with any other analyses using this tool. If deployed on the cloud or in a container, it would also be portable enough to run on larger datasets.

QIIME 2 (Bolyen *et al.* 2019) is a popular software package that provides command-line, Python, and graphical user interfaces for amplicon sequence analysis from raw FASTQ sequences to observation tables, statistical analyses, and interactive visualizations. QIIME 2 supports DADA2 (Callahan *et al.* 2016) and Deblur (Amir *et al.* 2017) plugins for denoising amplicon sequence data. Snakemake (Köster and Rahmann 2012) is a workflow management system that is popular in the bioinformatics community. Snakemake manages input and output files in a defined directory structure, with commands defined in a *Snakefile* as 'rules,' and parameters and initial input files set by the user in a configuration file. Snakemake ensures that only the commands required for requested output files not yet generated are run, saving time and computation when re-running part of a workflow.

Here, we present Tourmaline (github.com/aomlomics/tourmaline), an amplicon analysis pipeline that uses Snakemake to run QIIME 2 commands for core analysis and interactive visualization—plus workflow-specific commands that generate an HTML report of output and summary tables and figures of data and metadata—with rapid analysis aided by workflow iterability and scalability, support for multiple cores, a Docker container, and detailed documentation. After cloning the initial Tourmaline directory from GitHub and setting up the input files and parameters, only a few simple shell commands are required to execute the Tourmaline workflow. Outputs are stored in a standard directory structure that is the same for every Tourmaline run, facilitating data exploration and sharing, parameter optimization, and downstream analysis. Because of this standard directory structure, different runs that utilize different parameters (e.g., DADA2 truncation lengths) can be easily compared, facilitated by a helper script that makes a new copy of the Tourmine directory from an existing one. Every Tourmaline run produces an HTML report containing a summary of metadata and outputs, with links to web-viewable QIIME 2 visualization files. QIIME 2 artifact files can be fed directly into Python- and R-based analysis packages. In addition to running natively on Mac and Linux platforms, Tourmaline can be run in any computing environment using Docker containers. In this paper, we describe the Tourmaline workflow, and apply it to a downsampled 16S rRNA gene dataset from surface waters of Lake Erie. The tutorial includes guidance on evaluating output to refine parameters for the workflow and showcases the HTML report, interactive visualizations, and R- and Python-based analysis notebooks for biological insight into amplicon datasets.

Findings

Workflow

Overview. Tourmaline is a Snakemake-based bioinformatics workflow that operates in a defined directory structure (Fig. 1). Installation involves installing QIIME 2 and other dependencies or installing the Docker container. The starting directory structure is then cloned directly from GitHub and is built out through Snakemake commands, defined as 'rules' in *Snakefile*. Tourmaline provides seven high-level 'pseudo-rules' for each of DADA2 paired-end, DADA2 single-end, and Deblur (single-end), running denoising and taxonomic and diversity analy-

Compiled on: March 1, 2022.
Draft manuscript prepared by the author.

ses via QIIME 2 and other programs, encompassing commonly used analyses in microbiome and eDNA research. For each type of processing, there are four steps: (1) the *denoise* rule imports FASTQ data and runs denoising, generating a feature table and representative sequences; (2) the *taxonomy* rule assigns taxonomy to representative sequences; (3) the *diversity* rule does representative sequence curation, core diversity analyses, and alpha and beta group significance; and (4) the *report* rule generates an HTML report of the metadata, inputs, outputs, and parameters. Steps 2–4 have two modes each, *unfiltered* and *filtered*, thus making seven pseudo-rules total. The difference between the *unfiltered* and *filtered* commands is that in the *taxonomy_filtered* command, undesired taxonomic groups or individual sequences from the representative sequences and feature table are filtered (removed). The *diversity* and *report* rules are identical for *unfiltered* and *filtered* commands, except the outputs go into separate subdirectories. In addition to the 21 pseudo-rules (3 denoising methods with 7 pseudo-rules each), there are 47 regular rules defined in *Snakefile* that perform the actual QIIME 2, Python, and shell commands of the workflow (Fig. S1).

Test dataset. Tourmaline comes with a test dataset of 16S rRNA gene (bacteria/archaea) amplicon data from surface waters of Western Lake Erie in summer 2018 (see Methods). The sequence data were subsampled to 1000 sequences per sample to allow the entire workflow to run in ~10 minutes. This test dataset is used throughout the paper to demonstrate the capabilities of Tourmaline.

Documentation. Full instructions for using the Tourmaline workflow, including installation, cloning, and editing the config file, are described in the Tourmaline Wiki at github.com/aomlomics/tourmaline/wiki. Some experience with the command line, QIIME 2, and Snakemake is helpful to use Tourmaline; basic tutorials for each of these are provided at github.com/aomlomics/tutorials.

Installation. The workflow requires QIIME 2 (version 2021.2) plus several dependencies, which can be installed natively in a Conda environment (instructions on GitHub) or via a Docker container using the Docker image from DockerHub. Tourmaline is installed by cloning the GitHub repository to the current directory with *git clone https://github.com/aomlomics/tourmaline*. This step is repeated any time a new iteration of Tourmaline is needed, and new copies can be initialized using a helper script (described below).

Snakefile. As a Snakemake workflow, Tourmaline has as its core files (1) a *Snakefile* that provides all the commands (rules) that comprise the workflow and (2) a *config.yaml* file that provides the input files and parameters for the workflow. *Snakefile* contains all of the commands used by Tourmaline, which invoke QIIME 2 commands, helper scripts (see below), or generate output directly. The main analysis features and options supported by Tourmaline, as specified in *Snakefile*, are as follows:

- FASTQ sequence import using a manifest file, or use a pre-imported FASTQ .qza file.
- Denoising with DADA2 (Callahan *et al.* 2016) (paired-end and single-end) and Deblur (Amir *et al.* 2017) (single-end).
- Feature classification (taxonomic assignment) with options of naive Bayes (Bokulich *et al.* 2018), consensus BLAST (Camacho *et al.* 2008), and consensus VSEARCH (Rognes *et al.* 2016).
- Feature filtering by taxonomy, sequence length, feature ID, and abundance/prevalence.
- De novo multiple sequence alignment with MUSCLE (Edgar 2004), Clustal Omega (Sievers and Higgins 2014), or MAFFT (Katoh and Standley 2013) (with masking) and tree building with FastTree (Price, Dehal and Arkin 2009).
- Outlier detection with odseq (Jehl, Sievers and Higgins 2015).
- Interactive taxonomy barplot.
- Tree visualization using Empress (Cantrell *et al.* 2021).
- Alpha diversity, alpha rarefaction, and alpha group significance with four metrics: number of observed features, Faith's phylogenetic diversity, Shannon diversity, and Pielou's evenness.
- Beta diversity distances, principal coordinates, Emperor (Vázquez-Baeza *et al.* 2013) plots, and beta group significance (one metadata column) with four metrics: unweighted and weighted UniFrac (Lozupone *et al.* 2010), Jaccard distance, and Bray–Curtis distance.
- Robust Aitchison PCA and biplot ordination using DEICODE (Martino *et al.* 2019).

Config file. The configuration file *config.yaml* includes paths to input files and parameters for QIIME 2 commands and other steps. Default settings have been chosen to balance run performance and accuracy and to work with the test data. For user data, all parameters should be checked and possibly adjusted for appropriateness with the dataset; see Table S1, Fig. 1, and the Wiki section *Setup* for guidance.

Input files. Tourmaline requires three categories of input files: (1) Reference database: a FASTA file of reference sequences (*refseqs.fna*) and a tab-delimited file of taxonomy (*reftax.tsv*) for those sequences, or their imported QIIME 2 artifact equivalents (*refseqs.qza*, *reftax.qza*); (2) Amplicon data: demultiplexed FASTQ sequence files and FASTQ manifest file(s) (*manifest_pe.csv*, *manifest_se.csv*) mapping sample names to the location of the sequence files, or their imported QIIME 2 equivalents (*fastq_pe.qza*, *fastq_se.qza*); and (3) Metadata: a tab-delimited sample metadata file (*metadata.tsv*) with sample names in the first column matching those in the FASTQ manifest file. See the Wiki section *Setup* for guidance on input file paths and use of symbolic links to avoid storing multiple copies of large input files.

Run the workflow. The workflow is run using Snakemake commands. For example, if using DADA2 paired-end method without any filtering (see below), the commands would be (1) *snakemake dada2_pe_denoise*, (2) *snakemake dada2_pe_taxonomy_unfiltered*, (3) *snakemake dada2_pe_diversity_unfiltered*, and (4) *snakemake dada2_pe_report_unfiltered*. Alternatively, the entire workflow can be run at once with the last command, *snakemake dada2_pe_report_unfiltered*.

Outputs

The outputs of each step of Tourmaline are described following a test run with the Lake Erie test data that comes with the GitHub repository. For each command, the main parameters used and list of output files generated in those commands are provided (Fig. 2). Accompanying the list of output files is guidance for evaluating them to choose parameters for subsequent steps (Fig. 2), with screenshots of the Tourmaline-specific output files (Fig. 3) and both QIIME 2 and Tourmaline-specific output files (Fig. S3). A video version of the tutorial is also available on YouTube (https://youtu.be/1lFyeswXSX8).

Denoise. The first command is *snakemake dada2_pe_denoise* (Fig. 2), which imports the FASTQ files and reference database (if not already present in directory *01-imported*), summarizes the FASTQ data, runs denoising using DADA2, and summarizes the output. In addition to QIIME 2 visualizations of the feature table, representative sequences, and phylogenetic tree, Tourmaline generates a table and scatter plot (*repseqs_properties.tsv*, *repseqs_properties_describe.md*,

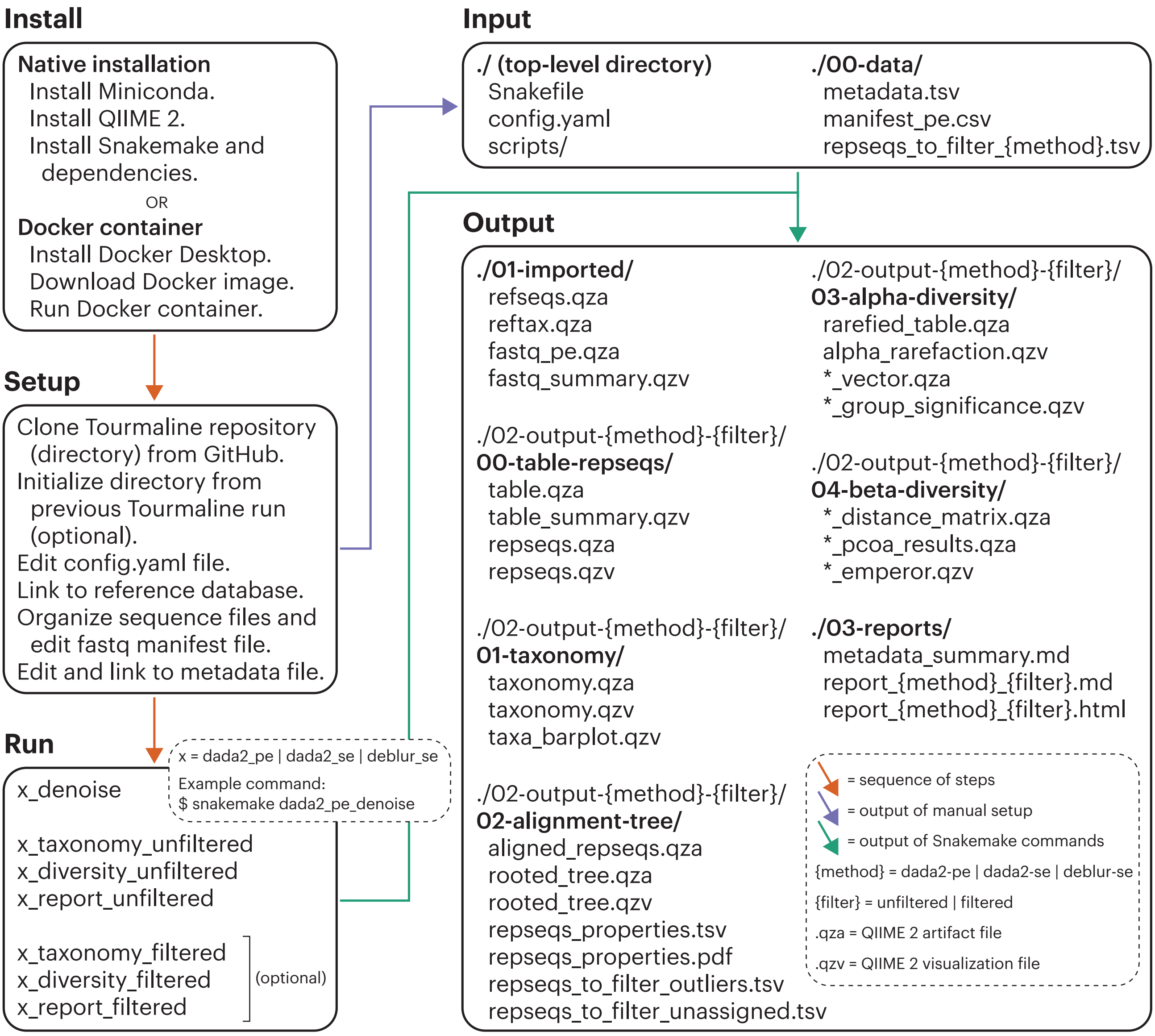

Figure 1. The Tourmaline workflow. Install natively (macOS, Linux) or using a Docker container. Setup by cloning the Tourmaline repository (directory) from GitHub, initializing the directory from a previous run (optional), editing the configuration file (*config.yaml*, Table S1), creating symbolic links to the reference database files, organizing the sequence files and/or editing the FASTQ manifest file, and editing and creating a symbolic link to the metadata file. Run by calling the Snakemake commands for *denoise*, *taxonomy*, *diversity*, and *report*—or running just the *report* command to generate all output if the parameters do not need to be changed between individual commands. It is recommended but not required to run the *unfiltered* commands before the *filtered* commands. The primary input and output files are listed. Detailed instructions for each step are provided in the Tourmaline Wiki (github.com/aomlomics/tourmaline/wiki).

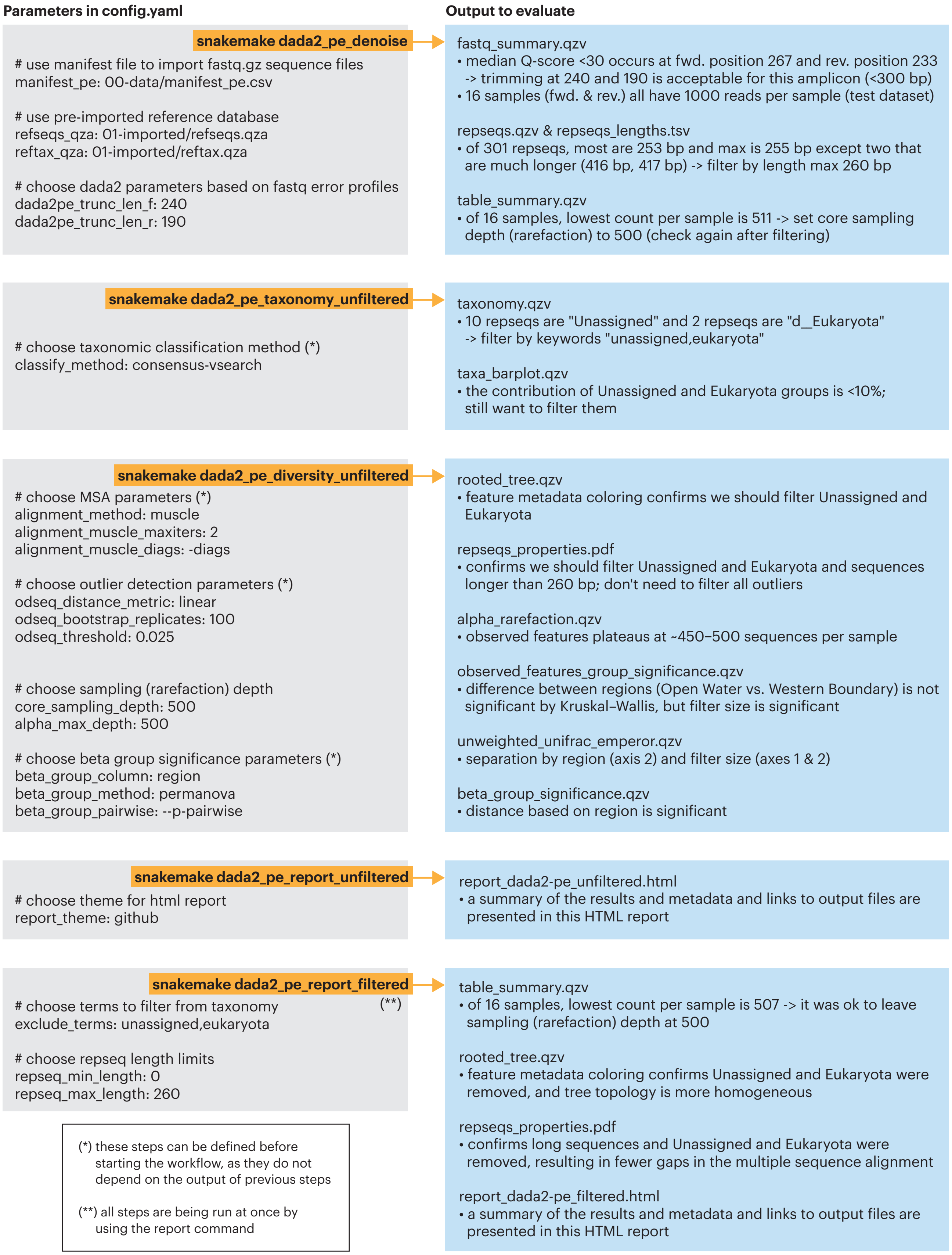

Figure 2. Step-by-step tutorial on Tourmaline using the provided test data, which is subsampled from the 16S rRNA amplicon data of a 2018 survey of Western Lake Erie. Key parameters in *config.yaml* and primary output for each command (pseudo-rule) are listed. Indicated output should be evaluated to determine the appropriate parameters for the next command. Evaluation of the primary outputs and rationale for parameter choice is shown for the test Lake Erie 16S rRNA data that comes with the Tourmaline repository. See Fig. S3 for screenshots of the primary output files.

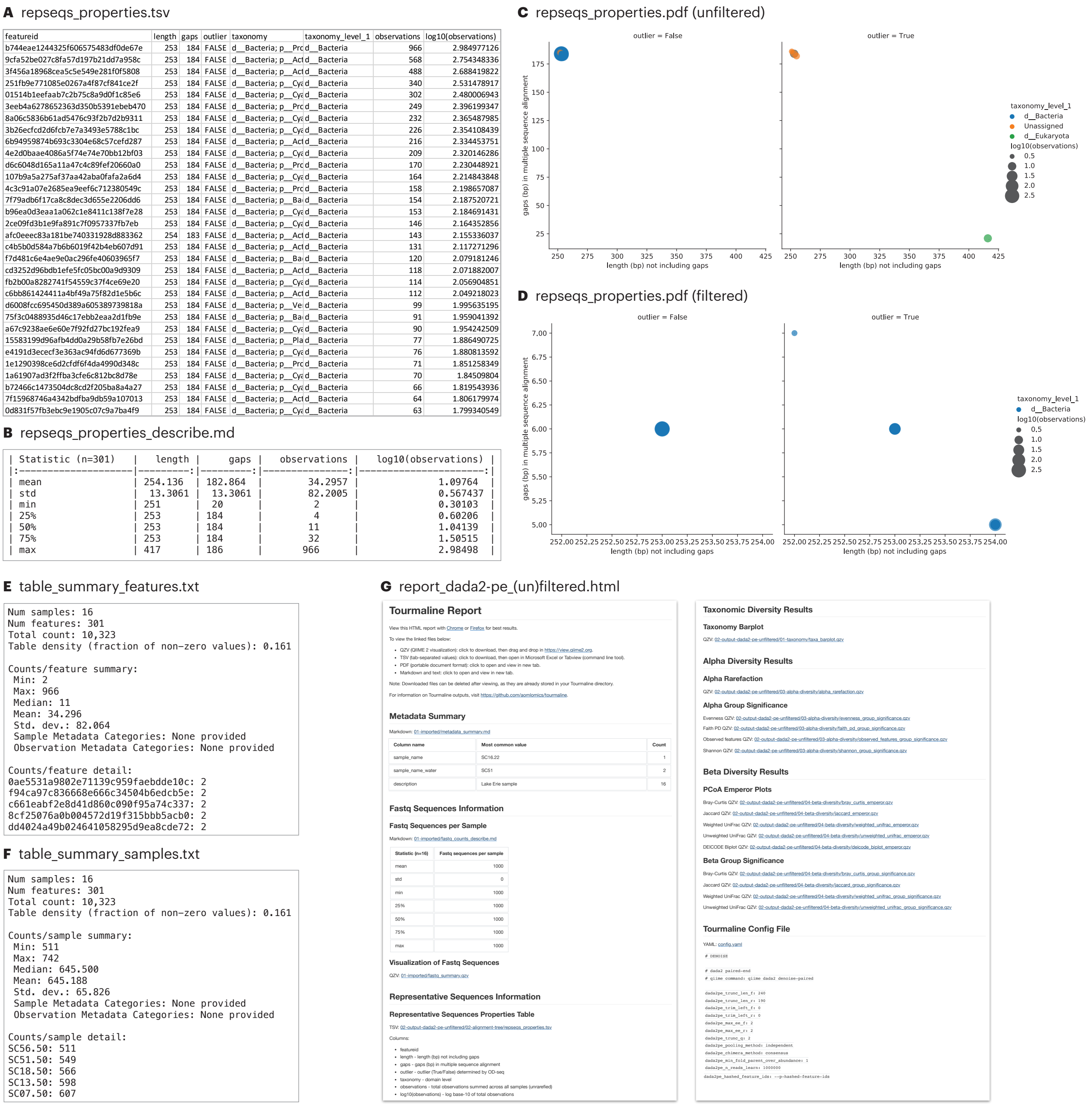

Figure 3. Example of the main outputs of the Tourmaline workflow beyond the QIIME 2 outputs. Contents in panels **A**, **E**, **F**, and **G** are truncated. Screenshots of additional output files are provided in Fig. S3. See Fig. 2 for commands, parameters, and guidance.

and *repseqs_properties.pdf*; Fig. 3A–D) of representative sequence properties, including sequence length, number of gaps in the multiple sequence alignment, outlier status, taxonomy, and total number of observations in the observation table. QC can be performed using *fastq_summary.qzv* (Fig. S3A) for quality scores and *reqseqs.qzv* (Fig. S3C) or *repseqs_lengths.tsv* for representative sequence lengths. The helper script *fastqc_per_base_sequence_quality_dropoff.py* can be run on the output of FastQC and MultiQC to estimate and set DADA2 or Deblur truncation lengths (see below) and then rerun the *denoise* step. Based on the representative sequence lengths, filtering by sequence length can also be set, to be used later in the *filtered* commands. Choice of appropriate sampling (rarefaction) depths for the parameters 'alpha_max_depth' and 'core_sampling_depth,' to be used in the *diversity* step, can be done by examining *table_summary_features.txt* (Fig. 3E), *table_summary_samples.txt* (Fig. 3F) and *table_summary.qzv* (Fig. S3B).

Taxonomy. The second command is *snakemake dada2_pe_taxonomy_unfiltered* (Fig. 2), which assigns taxonomy to the representative sequences using a naive Bayes classifier or consensus BLAST or VSEARCH method and generates an interactive taxonomy table and an interactive barplot of sample taxonomic composition. Choice of taxonomic groups to be filtered by keyword, to be used later with *filtered* commands, can be done by examining *taxonomy.qzv* (Fig. S3D) and *taxa_barplot.qzv* (Fig. S3E).

Diversity. The third command is *snakemake dada2_pe_diversity_unfiltered* (Fig. 2), which aligns representative sequences using one of three methods, computes outliers using odseq (Jehl, Sievers and Higgins 2015), and builds a phylogenetic tree. This step generates lists of representative sequences that have unassigned taxonomy and were computed to be outliers, summarizes and plots the representative sequence properties, performs alpha rarefaction, and runs alpha diversity and beta diversity analyses and group significance tests using a suite of metrics. Filtering parameters can be checked by examining *rooted_tree.qzv* (Fig. S3F) and *repseqs_properties.pdf* (Fig. S3G), if desired. Whether sampling depth was sufficient can be evaluated with *alpha_rarefaction.qzv* (Fig. S3I). Alpha and beta diversity patterns and statistically significant differences between groups can be evaluated with *observed_features_group_significance.qzv* (Fig. S3J; other alpha diversity metrics are also provided), *unweighted_unifrac_emperor.qzv* (Fig. S3H; other beta diversity metrics are also provided), and *beta_group_significance.qzv* (Fig. S3K).

Report. The fourth and final command is *snakemake dada2_pe_report_unfiltered* (Fig. 2), which creates a comprehensive HTML report of parameters, metadata, inputs, outputs, and visualizations in a single file. The file *report_dada2-pe_unfiltered.html* (Fig. 3G) can be viewed in a web browser, and the linked output files can be viewed in a browser or downloaded and opened with view.qiime2.org (.qzv files) or Microsoft Excel (.tsv files).

Filtering. After reviewing the *unfiltered* results—the taxonomy summary and taxa barplot, the representative sequence summary plot and table, and the list of unassigned and potential outlier representative sequences—the user may wish to filter (remove) certain representative sequences by taxonomic group or other properties. This is done by setting the filtering parameters in *config.yaml* and providing a list of any individual representative sequences to filter, then running the *filtered* commands of the workflow: *snakemake dada2_pe_taxonomy_filtered*, *snakemake dada2_pe_diversity_filtered*, and *snakemake dada2_pe_report_filtered* (Fig. 2). Among the *filtered* output, the user can check *table_summary.qzv* (Fig. S3L) to ensure that the sampling depth after filtering did not exclude samples, and examine *rooted_tree.qzv* (Fig. S3N) and *repseqs_properties.pdf* (Fig. S3O) to check that the desired representative sequences were filtered. All of the outputs can be viewed by opening *report_dada2-pe_filtered.html* (Fig. S3M) in a web browser.

Downstream analysis & meta-analysis

For users who wish to analyze their output further using Jupyter notebooks, we provide Python and R notebooks (github.com/aomlomics/tourmaline/tree/master/notebooks) pre-loaded with popular data analysis and visualization tools for those platforms. These notebooks come ready to run with Tourmaline output, using relative paths to take advantage of Tourmaline's defined output file structure. The notebooks are shown with the tutorial dataset that comes with Tourmaline. We also provide a Python notebook for meta-analysis, containing commands to merge outputs from multiple Tourmaline runs and then perform diversity analyses on the merged files.

Python Jupyter notebook. The Python Jupyter notebook (Fig. S2A) uses the QIIME 2 Visualization and Artifact object classes, loading Visualization and Artifact objects from the .qzv and .qza Tourmaline output files. Before running the notebook, the denoising method, filtering mode, and alpha and beta diversity metrics to be used can be specified by changing variable assignments at the beginning of the notebook. The notebook renders Visualization objects for the feature table summary, representative sequences summary, phylogenetic tree, taxonomy, taxa bar plot, alpha diversity group significance, and beta diversity principal coordinates analysis (PCoA) Emperor plot. Artifact objects can be viewed as a Pandas (McKinney 2010) DataFrame or Series. The notebook generates Pandas DataFrames for the feature table, taxonomy, reference sequence properties, and metadata, and a Pandas Series for alpha diversity. Static plots are generated from some of these tables using Seaborn (Qalieh *et al.* 2017).

R Jupyter notebook. The R Jupyter notebook (Fig. S2B) imports Tourmaline artifact (.qza) files using qiime2R (Bisanz 2018) and uses common R packages for analyzing and visualizing amplicon sequencing data, including phyloseq (Halfvarson *et al.* 2017b), tidyverse (Wickham *et al.* 2019), and vegan (Oksanen *et al.* 2020). The notebook covers how to import QIIME 2 count and taxonomy artifact files from Tourmaline into an R environment, merge and manipulate the resulting data frames into a single phyloseq object, and estimate and plot diversity metrics and taxonomy bar plots of the 16S community using phyloseq and other packages. As with the Python notebook, a set of variables can be specified at the beginning of the R notebook to define specific denoising, filtering, and diversity metrics. After reading in the metadata file and merging to a phyloseq object, we define plotting parameters that can be easily modified by the user to customize the R visualizations.

Meta-analysis notebook. The meta-analysis notebook (Fig. S2C) guides the user through running Tourmaline on two separate datasets, merging the outputs (feature tables, representative sequences, and taxonomies) and metadata, and performing some basic diversity analyses on the merged output. For simplicity, the two datasets are derived from the test data that comes with Tourmaline. The commands provided could be applied to any set of Tourmaline outputs that the user wishes to combine in a meta-analysis. The only requirement is that the sequenced region must be the same across the datasets for the results to make sense. This notebook is a simple example that demonstrates Tourmaline's capacity to facilitate merging of outputs and meta-analysis. Many additional analyses

are possible on the merged output, such as demonstrated in published microbiome meta-analyses (Thompson *et al.* 2017; Delgado-Baquerizo *et al.* 2018).

Helper scripts & parameter optimization

Tourmaline comes with several helper scripts that are run automatically with the workflow or run directly by the user. See the Wiki section *Setup* for more information.

Initialize a new Tourmaline directory. From the main directory of a newly cloned Tourmaline directory, the script *initialize_dir_from_existing_tourmaline_dir.sh* will copy *config.yaml* and *Snakefile* from an existing tourmaline directory, remove the test files, then copy the data files and symlinks from the existing Tourmaline directory. This is useful when performing a new analysis on the same dataset. The user can clone a new copy of Tourmaline, run this script to copy everything from the old copy to the new one, then make desired changes to the parameters.

Create a FASTQ manifest file. Two scripts help create the manifest file that points Tourmaline to the FASTQ sequence files. (1) *create_manifest_from_fastq_directory.py* creates a FASTQ manifest file from a directory of FASTQ files. (2) *match_manifest_to_metadata.py* takes an existing FASTQ manifest file and generates two new manifest files (paired-end and single-end) corresponding to the samples in the provided metadata file.

Determine optimal truncation length. If FastQC and MultiQC have been run for Read 1 and Read 2, *fastqc_per_base_sequence_quality_dropoff.py* will determine the position where median per-base sequence quality drops below some fraction (default: 0.90) of its maximum value. This is useful for defining 3' truncation positions in DADA2 and Deblur ('dada2pe_trunc_len_f,' 'dada2se_trunc_len,' and 'deblur_trim_length').

Parameter optimization. The helper scripts and Tourmaline's standard directory structure enable testing and comparison of different parameter sets to optimize a workflow. By making multiple copies of the directory and populating settings with *initialize_dir_from_existing_tourmaline_dir.sh* script, varying one or a small number of parameters, and running the workflow multiple times in parallel, outputs can be compared visually or programmatically to see the effects of parameter choices and choose a final set. To illustrate this, we analyzed the full dataset of the 2018 Lake Erie 16S rRNA study (BioProject PRJNA679730). Running *fastqc_per_base_sequence_quality_dropoff.py* had suggested that a forward truncation length of 240 bp and reverse truncation length of 190 bp would strike a balance between sequence length and quality, but we wanted to test a full range of truncation lengths. We tested the effects of varying the forward and reverse truncation lengths from 100 bp to 250 bp in 50-bp increments on the distribution of representative sequence length (Fig. S4A) and the number of reads assigned to Eukaryota (Fig. S4B), a group potentially amplified by these primers but with longer representative sequences. This analysis helped choose a set of truncation lengths that would capture a large diversity of target organisms.

Parallelization & benchmarks

A typical amplicon sequencing dataset is much larger than the test dataset that comes with the Tourmaline repository and will take considerably longer to process. To evaluate runtimes with a real-world dataset, we ran Tourmaline on the full dataset of the 2018 Lake Erie 16S rRNA study (BioProject PRJNA679730), which is the dataset from which the test dataset was subsampled. This dataset was sequenced with 2x300-bp Illumina MiSeq sequencing and consists of 96 samples having an average of 120,338 paired reads per sample, for a total of 11,552,448 paired reads. Processing was performed using the Tourmaline Docker container running on a 2017 iMac Pro with an 18-core 2.3-GHz Intel Xeon W processor and 64 GB RAM (32 GB RAM allocated for the Docker container). Speed improvements with parallelization were tested by running Snakemake with either 1 or 8 cores (parameter: *--cores*). Each main step in the workflow (*denoise*, *taxonomy*, *diversity*, and *report*; *unfiltered* commands) was run and timed separately. Times would be expected to be similar for *filtered* commands except that the *denoise* rule does not need to be rerun. The results (Table 1) show that a relatively large dataset of ~100 samples with ~100,000 sequences per sample can be processed with a single core in ~5 hours. Dramatic speed improvements are possible with multiple cores, with this same dataset being processed in ~2 hours when 8 cores were used.

Biological insights

The purpose of performing amplicon sequencing or metabarcoding is to reveal patterns of diversity, community structure, and biological (or environmental) drivers within diverse ecosystems. Whether the system of study is microbial communities in an environmental or biomedical setting or trace environmental DNA in an aquatic or terrestrial system, the kinds of biological questions being asked are similar. Tourmaline supports biological insight in two important ways: (1) by supporting the most popular analysis tools and packages in use today, with capacity to expand as new tools are developed; (2) by providing multiple ways to view the output, giving everyone from experts to novices a platform to visualize and query the output.

Through its core QIIME 2 functionality and downstream support for R and Python data science packages, Tourmaline enables analysis of the core metrics of microbial and eDNA diversity: taxonomic composition, within-sample diversity (alpha diversity), and between-sample diversity (beta diversity). Examining our analysis of the tutorial dataset (Fig. S3), we can see how Tourmaline facilitates insight into Western Lake Erie microbial communities. The interactive barplot (Fig. S3E) provides rapid insights: the most abundant bacterial families in the 5.0-μm fraction are Sporichthyaceae and SAR11 Clade III; the most abundant bacterial family in the 0.22-μm fraction is Cyanobiaceae (the toxic cyanobacterial family Microcystaceae is less abundant), with the largest component assigned as chloroplasts, which can be filtered in a subsequent run; at the domain level, a small fraction of unassigned and Eukaryota-assigned sequences are observed, which can also be filtered. The alpha diversity results show that the 5.0-μm fraction has greater within-sample diversity (number of observed features) than the 0.22-μm fraction (Fig. S3J) and that this diversity appears to be saturated, with a relatively small sampling depth of ~350 sequences per sample sufficient to observe these values (Fig. S3I). However, because a large fraction of the 0.22-μm sequences were identified as chloroplast, filtering out those sequences in a future run would be warranted and provide more accurate diversity results. The beta diversity results show that 16S communities are distinguished both by location (Open Water vs. Western Boundary) and size fraction (0.22-μm vs. 5.0-μm) (Fig. S3H). From this simple tutorial dataset, we demonstrate the use of Tourmaline to analyze environmental amplicon data, in this case revealing the importance of pore size when filtering water samples for microbial sequencing and the presence of spatial variability (regardless of pore size) among microbial communities in Lake Erie.

The ability to view Tourmaline output files with multiple

Table 1. Benchmarking and parallel processing results from running the full 2018 Lake Erie 16S rRNA dataset through Tourmaline with either 1 or 8 cores using a Tourmaline Docker container allocated with 32 GB RAM running on an 18-core iMac Pro (2017). The Snakemake command used the parameter *--cores 1* or *--cores 8*, and parameters in *config.yaml* specifying the number of threads for individual rules were set to 1 or 8, respectively. Times reported are the elapsed real time between invocation and termination and are reported as HH:MM:SS. Times do not include the initial step of importing FASTQ files into a QIIME 2 archive (*fastq_pe.qza*), which took ~2 minutes. Parameters shown in the last column are those most relevant to the runtimes. Unless otherwise noted, the parameters used were the defaults in *config.yaml*.

Rule	Time (-- cores 1)	Time (--cores 8)	Parameters & details
dada2_pe_denoise	02:05:43	00:38:10	method: dada2-pe<br><br>96 samples * 120,338 sequences per sample = 11,552,448 total sequences
dada2_pe_taxonomy_unfiltered	01:31:55	00:12:39	classify_method: consensus-vsearch<br><br>12,379 representative sequences
dada2_pe_diversity_unfiltered	01:18:09	01:13:49	alignment_method: muscle<br>alignment_muscle_maxiters: 2<br>alignment_muscle_diags: -diags<br>odseq_distance_metric: linear<br>odseq_bootstrap_replicates: 100<br>odseq_threshold: 0.025<br><br>12,379 representative sequences (lengths: min 240, max 418, avg 258)
dada2_pe_report_unfiltered	00:00:05	00:00:05	–
**Total**	04:55:52	02:04:43	–

interfaces provides access to researchers with different backgrounds. For users experienced with the Unix command line, the diverse output file types, organized in a defined directory structure, can be queried and analyzed using a wide array of data science tools; anything that can be done with QIIME 2 output and other common sequence diversity output files types can be done with Tourmaline output. For data scientists most comfortable with Jupyter notebooks, the prebuilt Python and R notebooks come ready to work with Tourmaline output and rapidly enable biological discovery from amplicon data. For casual users, the web-based report and QIIME 2 visualizations provide a user-friendly onramp to view and interact with the data. This last mode of interacting with the output opens up amplicon analysis to a wider range of users than is typically possible, from collaborators to students to anyone with limited data science expertise. This increased accessibility can accelerate the pace of discovery by increasing the diversity of researchers able to work with the data.

Conclusions

Tourmaline provides a comprehensive platform for amplicon sequence analysis that enables rapid and iterable processing and inference of microbiome and eDNA metabarcoding data. It has multiple features that enhance usability and interoperability:

- **Portability.** Native support for Linux and macOS in addition to Docker containers, enabling it to run on desktop, cluster, and cloud computing platforms.
- **QIIME 2.** The core commands of Tourmaline, including the DADA2 and Deblur packages, are all commands of QIIME 2, one of the most popular amplicon sequence analysis software tools available. Users can print all of the QIIME 2 and other shell commands of a workflow before or while running the workflow.
- **Snakemake.** Managing the workflow with Snakemake provides several benefits:
  - **Configuration file** contains all parameters in one file, so the user can see what the workflow is doing and make changes for a subsequent run.
  - **Directory structure** is the same for every Tourmaline run, so the user always knows where outputs are.
  - **On-demand commands** mean that only the commands required for output files not yet generated are run, saving time and computation when re-running part of a workflow.
- **Parameter optimization.** The configuration file and standard directory structure make it simple to test and compare different parameter sets to optimize a workflow.
- **Visualizations and reports.** Every Tourmaline run produces an HTML report containing a summary of metadata and outputs, with links to web-viewable QIIME 2 visualization files.
- **Downstream analysis.** Analyze the output of single or multiple Tourmaline runs programmatically, with qiime2R in R or the QIIME 2 Artifact API in Python, using the provided R and Python notebooks or other code.

Through its streamlined workflow and broad functionality, Tourmaline enables rapid response and biological discovery in any system where amplicon sequencing is applied, from biomedical and environmental microbiology to eDNA for fisheries and protected or invasive species. The QIIME 2-based interactive visualizations it generates allow users to quickly compare differences between samples and groups of samples in their taxonomic composition, within-sample diversity (al-

pha diversity), and between-sample diversity (beta diversity), which are core metrics of microbial and eDNA diversity. Tourmaline's unique HTML report and pre-loaded Jupyter notebooks provide ready access to the output, supporting less-experienced researchers and data scientists alike, and the output files are ready to be loaded into a variety of downstream tools in the QIIME 2 and phyloseq ecosystems. Future improvements to the workflow will include support for new QIIME 2 releases and plugins, better integration with Snakemake, possibly including Conda integration and connecting Snakemake's reporting ability with QIIME 2's provenance tracking, and enhanced support for cloud computing environments. With its existing features that balance usability, functionality, iterability, and scalability, and with continued development with support from the research community, Tourmaline will be a valuable and longstanding tool for amplicon sequence analysis.

Methods

Sample collection and DNA extraction

Water samples were collected using a long-range autonomous underwater vehicle (LRAUV, Monterey Bay Aquarium Research Institute) equipped with a third-generation environmental sample processor (3G-ESP, Monterey Bay Aquarium Research Institute) (Pargett *et al.* 2015). For each sample, water was filtered through stacked 5.0-μm (top) and 0.22-μM (bottom) Durapore filters (EMD Millipore) held in custom 3G-ESP 'archive' cartridges and preserved in-cartridge with RNAlater (Thermo Fisher). DNA extraction was performed using the Qiagen DNeasy Blood and Tissue kit.

Amplicon sequencing

Extracted DNA was amplified using a BiooScientific NEXTFlex 16S V4 Amplicon-Seq Kit 2.0 (NOVA-520999/Custom NOVA-4203-04] (BiooScientific, Austin, TX, USA). Target-specific regions of the forward and reverse primers in the 16S V4 Amplicon-Seq kit were custom ordered to follow the Earth Microbiome Project 16S Illumina Amplicon Protocol: forward primer 515F 5'-GTGYCAGCMGCCGCGGTAA-3' (Parada, Needham and Fuhrman 2016) and reverse primer 806R 5'-GGACTACNVGGGTWTCTAAT-3' (Apprill *et al.* 2015). 16S rRNA amplicons were pooled and sequenced on an Illumina MiSeq with 2 x 300-bp chemistry at the University of Michigan Advanced Genomics Core (brcf.medicine.umich.edu/cores/dna-sequencing). Demultiplexed sequences were deposited in NCBI under BioProject PRJNA679730.

Supporting tables, figures, and videos

Supporting tables and figures are attached to the end of this manuscript.

Video 1. Tutorial covering installing and running the Tourmaline workflow, reviewing the report, and viewing QIIME 2 visualization files. https://youtu.be/xKfOxrXBXYQ

Availability of supporting source code

- Tourmaline is available by cloning the GitHub repository at https://github.com/aomlomics/tourmaline. Tourmaline is released under a 3-clause BSD license.
- Full installation and usage instructions are available from the Tourmaline Wiki at https://github.com/aomlomics/tourmaline/wiki.
- A Docker image is available from DockerHub at https://hub.docker.com/r/aomlomics/tourmaline. This docker image is based on a QIIME 2 Docker image hosted at https://quay.io/repository/qiime2/core?tag=2021.2, and the QIIME 2 license can be found at https://github.com/qiime2/qiime2/blob/master/LICENSE.
- Additional code and data from the full 2018 Lake Erie dataset is available at https://github.com/aomlomics/erie.

Availability of supporting data

- The test 16S dataset (1000 sequences per sample) is available directly from the GitHub repository at https://github.com/aomlomics/tourmaline.
- Reference databases are available for 16S rRNA at https://docs.qiime2.org/2021.2/data-resources/#silva-16s-18s-rrna and for 18S–ITS rRNA at https://unite.ut.ee/repository.php.
- Output for the tutorial using the included test data are available from Zenodo at https://doi.org/10.5281/zenodo.5044532.
- A snapshot of the GitHub repository will be available from Zenodo upon publication.

Abbreviations

ASV: amplicon sequence variant; COI: cytochrome oxidase I; DAG: directed acyclic graph; eDNA: environmental DNA; ITS: internal transcribed spacer; NMDS: non-metric dimensional scaling; PCoA: principal coordinates analysis; PCR: polymerase chain reaction; QIIME: Quantitative Insights Into Microbial Ecology.

Competing interests

The authors declare that they have no competing interests.

Funding

This work was supported by awards NA06OAR4320264 06111039 to the Northern Gulf Institute (NGI) at Mississippi State University, NA17OAR4320152 (contribution number 1168) to the Cooperative Institute for Great Lakes Research (CIGLR) at the University of Michigan, and a National Oceanographic Partnership Program grant to the Cooperative Institute for Marine and Atmospheric Studies (CIMAS) at the University of Miami from NOAA's Office of Oceanic and Atmospheric Research (OAR), U.S. Department of Commerce. Support was also provided by the OAR 'Omics Program, Ocean Technology Development, and the National Oceanographic Partnership Program (NOPP). G. Sanderson contributed to this work as part of a NOAA Ernest F. Hollings Scholarship summer internship.

Author contributions

The Tourmaline workflow was designed and developed by L.R. Thompson. Code was tested by L.R. Thompson, N.V. Patin, S.R. Anderson, and S.J. Lim. The Docker image was built by N.V. Patin and L.R. Thompson. Data analysis and visualization of the case study were done by S.R. Anderson. Analysis notebooks were developed by L.R. Thompson, S.R. Anderson, and G. Sanderson. Samples were collected by P.A. Den Uyl and K.D. Goodwin. DNA was extracted and prepared for sequencing by P.A. Den Uyl. The manuscript was written by L.R. Thompson,

S.R. Anderson, P.A. Den Uyl, S.J. Lim, and K.D. Goodwin.

Acknowledgements

We thank Mehrbod Estaki and Jean Lim for testing of the Tourmaline workflow and feedback on the Tourmaline GitHub repository. We also thank Reagan Errera, Subba Rao Chaganti, Jim Birch, Greg Doucette, for project planning and sample and data collection for the Lake Erie 3G-ESP project. We thank Gregory Dick, Colleen Yancey, and McKenzie Powers for discussions on *Microcystis* diversity and genomics.

References

Abarenkov K, Nilsson RH, Larsson K *et al.* The UNITE database for molecular identification of fungi – recent updates and future perspectives. *New Phytologist* 2010;**186**:281–5.

Ahn J, Sinha R, Pei Z *et al.* Human Gut Microbiome and Risk for Colorectal Cancer. *JNCI: Journal of the National Cancer Institute* 2013;**105**:1907–11.

Amir A, McDonald D, Navas-Molina JA *et al.* Deblur rapidly resolves single-nucleotide community sequence patterns. *mSystems* 2017;**2**, DOI: 10.1128/msystems.00191-16.

Apprill A, McNally S, Parsons R *et al.* Minor revision to V4 region SSU rRNA 806R gene primer greatly increases detection of SAR11 bacterioplankton. *Aquatic Microbial Ecology* 2015;**75**:129–37.

Asbun AA, Besseling MA, Balzano S *et al.* Cascabel: a flexible, scalable and easy-to-use amplicon sequence data analysis pipeline. *bioRxiv* 2019:809384.

Bisanz JE. qiime2R: Importing QIIME2 artifacts and associated data into R sessions. 2018.

Bokulich NA, Kaehler BD, Rideout JR *et al.* Optimizing taxonomic classification of marker-gene amplicon sequences with QIIME 2's q2-feature-classifier plugin. *Microbiome* 2018;**6**:90.

Bolyen E, Rideout JR, Dillon MR *et al.* Reproducible, interactive, scalable and extensible microbiome data science using QIIME 2. *Nature Biotechnology* 2019;**37**:852–7.

Callahan BJ, McMurdie PJ, Rosen MJ *et al.* DADA2: High-resolution sample inference from Illumina amplicon data. *Nature Methods* 2016;**13**:581–3.

Camacho C, Coulouris G, Avagyan V *et al.* BLAST+: architecture and applications. *BMC Bioinformatics* 2008;**10**:421–1.

Cantrell K, Fedarko MW, Rahman G *et al.* EMPress Enables Tree-Guided, Interactive, and Exploratory Analyses of Multi-omic Data Sets. *mSystems* 2021;**6**, DOI: 10.1128/msystems.01216-20.

Compson ZG, McClenaghan B, Singer GAC *et al.* Metabarcoding From Microbes to Mammals: Comprehensive Bioassessment on a Global Scale. *Frontiers in Ecology and Evolution* 2020;**8**:581835.

Consortium THMP. Structure, function and diversity of the healthy human microbiome. *Nature* 2012;**486**:207–14.

Curd EE, Gold Z, Kandlikar GS *et al.* Anacapa Toolkit: an environmental DNAtoolkit for processing multilocus metabarcode datasets. *Methods in Ecology and Evolution* 2019:2041-210X.13214.

Deiner K, Bik HM, Mächler E *et al.* Environmental DNA metabarcoding: Transforming how we survey animal and plant communities. *Molecular ecology* 2017;**26**:5872–95.

Delgado-Baquerizo M, Oliverio AM, Brewer TE *et al.* A global atlas of the dominant bacteria found in soil. *Science* 2018;**359**:320–5.

Dickie IA, Boyer S, Buckley HL *et al.* Towards robust and repeatable sampling methods in eDNA-based studies. *Molecular Ecology Resources* 2018;**18**:940–52.

Edgar RC. MUSCLE: multiple sequence alignment with high accuracy and high throughput. *Nucleic Acids Research* 2004;**32**:1792–7.

Halfvarson J, Brislawn CJ, Lamendella R *et al.* Dynamics of the human gut microbiome in Inflammatory Bowel Disease. *Nature microbiology* 2017a;**2**:17004–4.

Halfvarson J, Brislawn CJ, Lamendella R *et al.* Dynamics of the human gut microbiome in inflammatory bowel disease. *Nature Microbiology* 2017b;**2**:17004.

Harper LR, Buxton AS, Rees HC *et al.* Prospects and challenges of environmental DNA (eDNA) monitoring in freshwater ponds. *Hydrobiologia* 2019;**826**:25–41.

Hupfauf S, Etemadi M, Juárez MF-D *et al.* CoMA – an intuitive and user-friendly pipeline for amplicon-sequencing data analysis. *PLoS ONE* 2020;**15**:e0243241.

Jehl P, Sievers F, Higgins DG. OD-seq: outlier detection in multiple sequence alignments. *BMC Bioinformatics* 2015;**16**:269.

Kartzinel TR, Hsing JC, Musili PM *et al.* Covariation of diet and gut microbiome in African megafauna. *Proceedings of the National Academy of Sciences* 2019;**116**:23588–93.

Katoh K, Standley DM. MAFFT Multiple Sequence Alignment Software Version 7: Improvements in Performance and Usability. *Molecular Biology and Evolution* 2013;**30**:772–80.

Köster J, Rahmann S. Snakemake—a scalable bioinformatics workflow engine. *Bioinformatics (Oxford, England)* 2012;**28**:2520–2.

Leray M, Yang JY, Meyer CP *et al.* A new versatile primer set targeting a short fragment of the mitochondrial COI region for metabarcoding metazoan diversity: application for characterizing coral reef fish gut contents. *Frontiers in Zoology* 2013;**10**:34.

Lozupone C, Lladser ME, Knights D *et al.* UniFrac: an effective distance metric for microbial community comparison. *The ISME Journal* 2010;**5**:169–72.

Martino C, Morton JT, Marotz CA *et al.* A Novel Sparse Compositional Technique Reveals Microbial Perturbations. *mSystems* 2019;**4**:e00016-19.

McKinney W. Data structures for statistical computing in Python. *Proceedings of the 9th Python in Science Conference*. Vol 445. 2010, 51–6.

Miya M, Sato Y, Fukunaga T *et al.* MiFish, a set of universal PCR primers for metabarcoding environmental DNA from fishes: detection of more than 230 subtropical marine species. *Royal Society Open Science* 2015;**2**:150088.

Oksanen J, Blanchet FG, Friendly M *et al.* Package 'vegan': Community Ecology Package. 2020.

Parada AE, Needham DM, Fuhrman JA. Every base matters: assessing small subunit rRNA primers for marine microbiomes with mock communities, time series and global field samples. *Environmental Microbiology* 2016;**18**:1403–14.

Pargett DM, Birch JM, Preston CM *et al.* Development of a mobile ecogenomic sensor. *OCEANS 2015 - MTS/IEEE Washington* 2015:1–6.

Price MN, Dehal PS, Arkin AP. FastTree: Computing Large Minimum Evolution Trees with Profiles instead of a Distance Matrix. *Molecular Biology and Evolution* 2009;**26**:1641–50.

Qalieh MW, Botvinnik O, O'Kane D *et al.* mwaskom/seaborn: v0.8.1 (September 2017). 2017, DOI: 10.5281/zenodo.883859.

Reiter T, Brooks† PT, Irber† L *et al.* Streamlining data-intensive biology with workflow systems. *GigaScience* 2021;**10**, DOI: 10.1093/gigascience/giaa140.

Rognes T, Flouri T, Nichols B *et al.* VSEARCH: a versatile open source tool for metagenomics. *PeerJ* 2016;**4**:e2584.

Ruppert KM, Kline RJ, Rahman MS. Past, present, and future perspectives of environmental DNA (eDNA) metabarcoding: A systematic review in methods, monitoring, and applications of global eDNA. *Global Ecology and Conservation* 2019;**17**:e00547.

Sievers F, Higgins DG. Multiple Sequence Alignment Methods. Russel DJ (ed.). *Methods in Molecular Biology* 2014;**1079**:105–16.

Straub D, Blackwell N, Langarica-Fuentes A *et al.* Interpretations of Environmental Microbial Community Studies Are Biased by the Selected 16S rRNA (Gene) Amplicon Sequencing Pipeline. *Frontiers in Microbiology* 2020;**11**:550420.

Sunagawa S, Coelho LP, Chaffron S *et al.* Structure and function of the global ocean microbiome. *Science* 2015;**348**:1261359–9.

Thompson LR, Sanders JG, McDonald D *et al.* A communal catalogue reveals Earth's multiscale microbial diversity. *Nature* 2017;**551**:457–63.

Thomsen PF, Willerslev E. Environmental DNA – An emerging tool in conservation for monitoring past and present biodiversity. *Biological Conservation* 2015;**183**:4–18.

Tian R, Imanian B. ASAP 2: a pipeline and web server to analyze marker gene amplicon sequencing data automatically and consistently. *BMC Bioinformatics* 2022;**23**:27.

Turnbaugh PJ, Ley RE, Mahowald MA *et al.* An obesity-associated gut microbiome with increased capacity for energy harvest. *Nature* 2006;**444**:1027–31.

Vargas C de, Audic S, Henry N *et al.* Eukaryotic plankton diversity in the sunlit ocean. *Science* 2015;**348**:1261605.

Vázquez-Baeza Y, Pirrung M, Gonzalez A *et al.* EMPeror: a tool for visualizing high-throughput microbial community data. *GigaScience* 2013;**2**:16.

Weißbecker C, Schnabel B, Heintz-Buschart A. Dadasnake, a Snakemake implementation of DADA2 to process amplicon sequencing data for microbial ecology. *GigaScience* 2020;**9**:giaa135.

Wickham H, Averick M, Bryan J *et al.* Welcome to the Tidyverse. *Journal of Open Source Software* 2019;**4**:1686.

Zafeiropoulos H, Viet HQ, Vasileiadou K *et al.* PEMA: a flexible Pipeline for Environmental DNA Metabarcoding Analysis of the 16S/18S ribosomal RNA, ITS, and COI marker genes. *GigaScience* 2020;**9**, DOI: 10.1093/gigascience/giaa022.

Zaiko A, Martinez JL, Schmidt-Petersen J *et al.* Metabarcoding approach for the ballast water surveillance – An advantageous solution or an awkward challenge? *Marine Pollution Bulletin* 2015;**92**:25–34.

Table S1. Parameters in the configuration file, *config.yaml*, that the user may edit as necessary. Additional parameters not shown may also be edited. The default configuration file is provided in the top level of the GitHub repository. The file format of *config.yaml*, YAML (yet another markup language), is a simple markup language that is used by Snakemake to specify parameters for a workflow.

Parameter	Description	Recommendation	Help
dada2pe_trunc_len_f<br>dada2pe_trunc_len_r<br>dada2se_trunc_len	Truncate bases (integer) from the 3' (right) ends of reads in DADA2.	Choose values that maximize length but remove low-quality ends. Note that DADA2 paired-end mode requires a minimum overlap of 12 bp to merge Read 1 and Read 2. See the section below "Sequence quality control and choice of truncation length" for instructions on using the included script fastqc_per_base_sequence_quality_dropoff.py.	dada2 denoise-paired; dada2 denoise-single
dada2pe_trim_left_f<br>dada2pe_trim_left_f<br>dada2se_trim_left	Trim bases (integer) from the 5' (left) ends of reads in DADA2.	Depending on your amplicon sequencing method, and if trimming was not done prior to running Tourmaline, you may have primer sequences, indexes, and/or adapters on the 5' ends of your reads. If so, set this parameter to remove those bases. If not, set this parameter to zero. Note that 5' trimming (this parameter) is done after 3' truncation (above parameter).	dada2 denoise-paired; dada2 denoise-single
deblur_trim_length	Truncate bases (integer) from the 3' (right) ends of reads in Deblur.	Choose values that maximize length but remove low-quality ends. See the section below "Sequence quality control and choice of truncation length" for instructions on using the included script fastqc_per_base_sequence_quality_dropoff.py.	deblur denoise-other
dada2pe_pooling_method<br>dada2se_pooling_method	DADA2 pooling method.	Choose pseudo for pseudo-pooling or independent for no pooling.	dada2 denoise-paired; dada2 denoise-single
dada2pe_chimera_method<br>dada2se_chimera_method	DADA2 chimera method.	Choose pooled if pseudo-pooling otherwise consensus or none.	dada2 denoise-paired; dada2 denoise-single
alignment_method	Multiple sequence alignment method.	Choose muscle or clustalo for best accuracy or mafft for faster results.	muscle; clustalo; mafft
classify_method	Taxonomic classification method.	Choose naive-bayes for best accuracy or consensus-blast for faster results.	feature-classifier
exclude_terms	Filter terms (taxa) from taxonomy.	Specify terms (comma-separated, no spaces) to find in taxonomy and filter out (case-insensitive), or provide a nonsense term to skip this step when filtering.	taxa filter-seqs
repseq_min_length<br>repseq_max_length	Set minimum and maximum sequence lengths to filter representative sequences by.	Limits are inclusive, i.e., sequences will be retained if greater than or equal to minimum, less than or equal to maximum. Leave defaults (0, 10000) to do no filtering.	taxa filter-seqs
repseq_min_abundance<br>repseq_min_prevalence	set minimum abundance and prevalence limits to filter representative sequences by.	Limit is inclusive, i.e., sequences will be retained if greater than or equal to minimum. Leave default (0) to do no filtering.	taxa filter-seqs
odseq_distance_metric	Distance metric for odseq.	Choose metric from: linear, affine.	odseq
odseq_bootstrap_replicates	Number (integer) of bootstrap replicates for odseq.	Choose more replicates for more robust detection of outliers, fewer replicates for faster processing.	odseq
odseq_threshold	Threshold (float) for bootstrap probability distribution for odseq.	Probability to be at the right of the bootstrap scores distribution when computing outliers. Tune this parameter depending on the diversity and occurrence of outliers in the MSA.	odseq
core_sampling_depth	Rarefaction depth (integer) for core diversity metrics.	Choose a value that balances sequencing depth (more is better) with number of samples retained (more is better).	diversity core-metrics-phylogenetic
alpha_max_depth	Rarefaction depth (integer) for alpha rarefaction.	Choose a value that balances sequencing depth (more is better) with number of samples retained (more is better).	diversity alpha-rarefaction
beta_group_column	Column (text) in your metadata to test beta-diversity group significance.	Choose a category that may differentiate your samples. This analysis can be rerun with different columns by renaming the output file and changing the value in config.yaml before running again.	diversity beta-group-significance
report_theme	HTML report theme.	Choose from: github, gothic, newsprint, night, pixyll, whitey.	Typora theme gallery

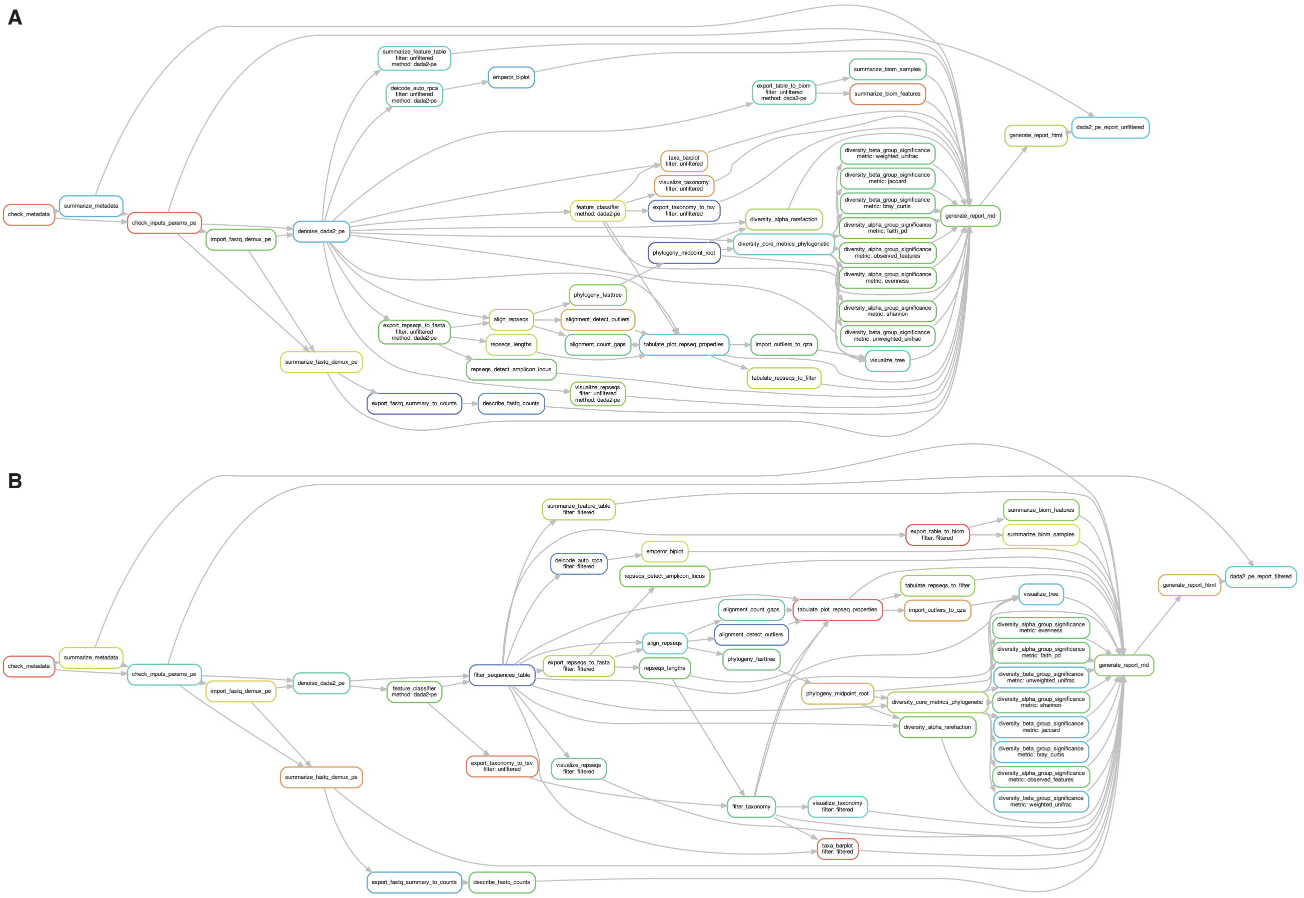

Figure S1. Directed acyclic graphs (DAGs) of the Tourmaline workflow for the DADA2 paired-end method from start to report with (a) *unfiltered* commands and (b) *filtered* commands. This figure was generated from the test data that comes with the repository by running the commands (a) *snakemake dada2_pe_report_unfiltered --dag | dot -Tpdf -Grankdir=LR -Gnodesep=0.1 -Granksep=0.1 > dag_pe_report_unfiltered.pdf* and (b) *snakemake dada2_pe_report_filtered --dag | dot -Tpdf -Grankdir=LR -Gnodesep=0.1 -Granksep=0.1 > dag_pe_report_filtered.pdf*. For a simpler graph, substitute *--rulegraph* for *--dag* in the above commands.

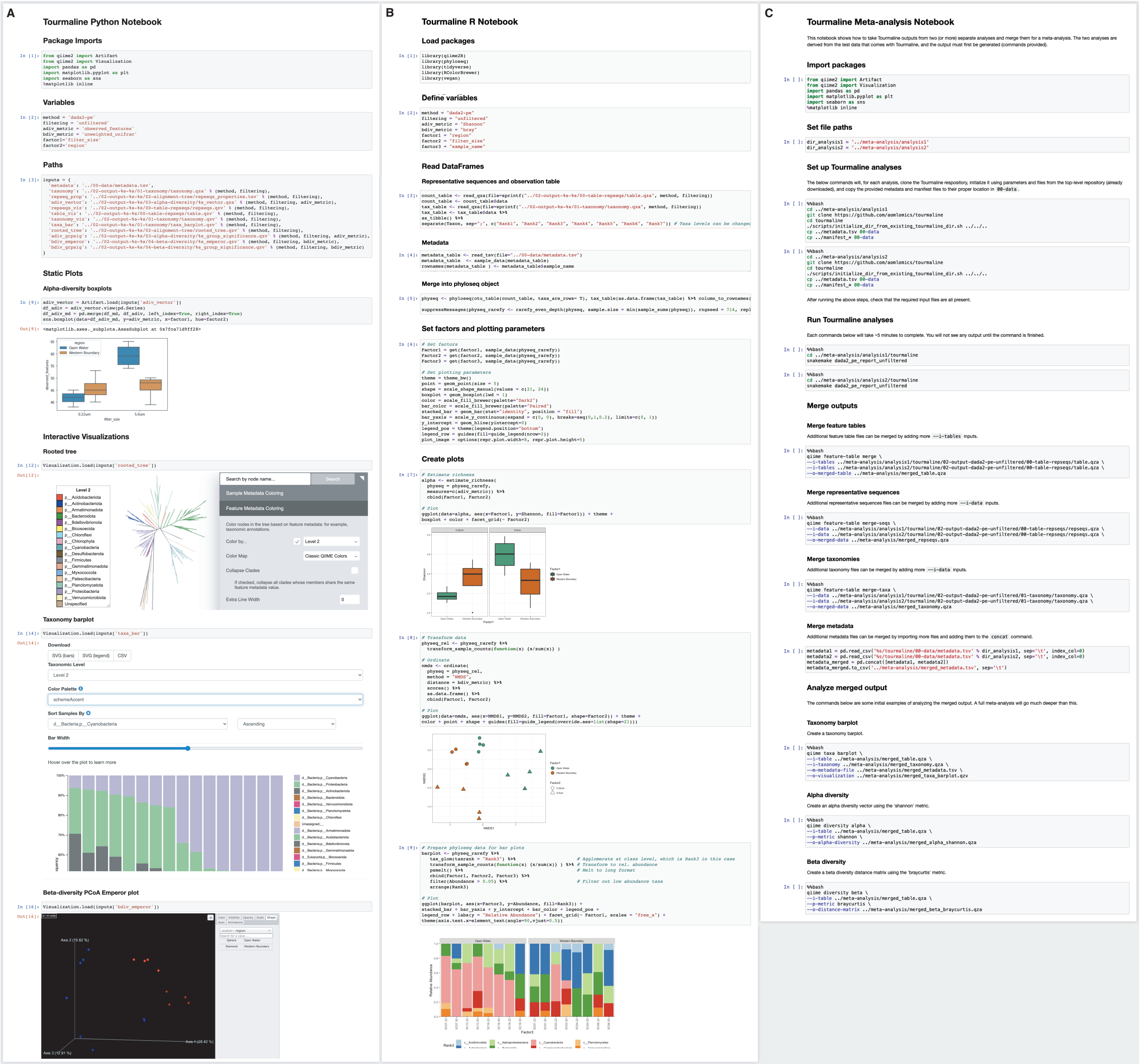

Figure S2. Screenshots of Tourmaline's included Python and R Jupyter notebooks running the provided test data. Both notebooks are designed to run out-of-the-box with the Tourmaline output from any dataset. (**A**) The Tourmaline Python notebook loads and displays sample metadata, feature metadata (representative sequences properties and taxonomy), static plots generated by Seaborn, and interactive QIIME 2 visualizations. (**B**) The Tourmaline R notebook demonstrates how to load .qza files (counts and taxonomy) into R, merge files with metadata into a single phyloseq object, and generate high-quality visualizations of community diversity and taxonomy using phyloseq and suite of tidyverse packages (e.g., ggplot2). (**C**) The Tourmaline meta-analysis notebook walks through the merging of two sets of Tourmaline outputs and performing some basic diversity analyses on the merged files. The number of processed datasets being merged in the meta-analysis can be increased by adding additional inputs to the commands.

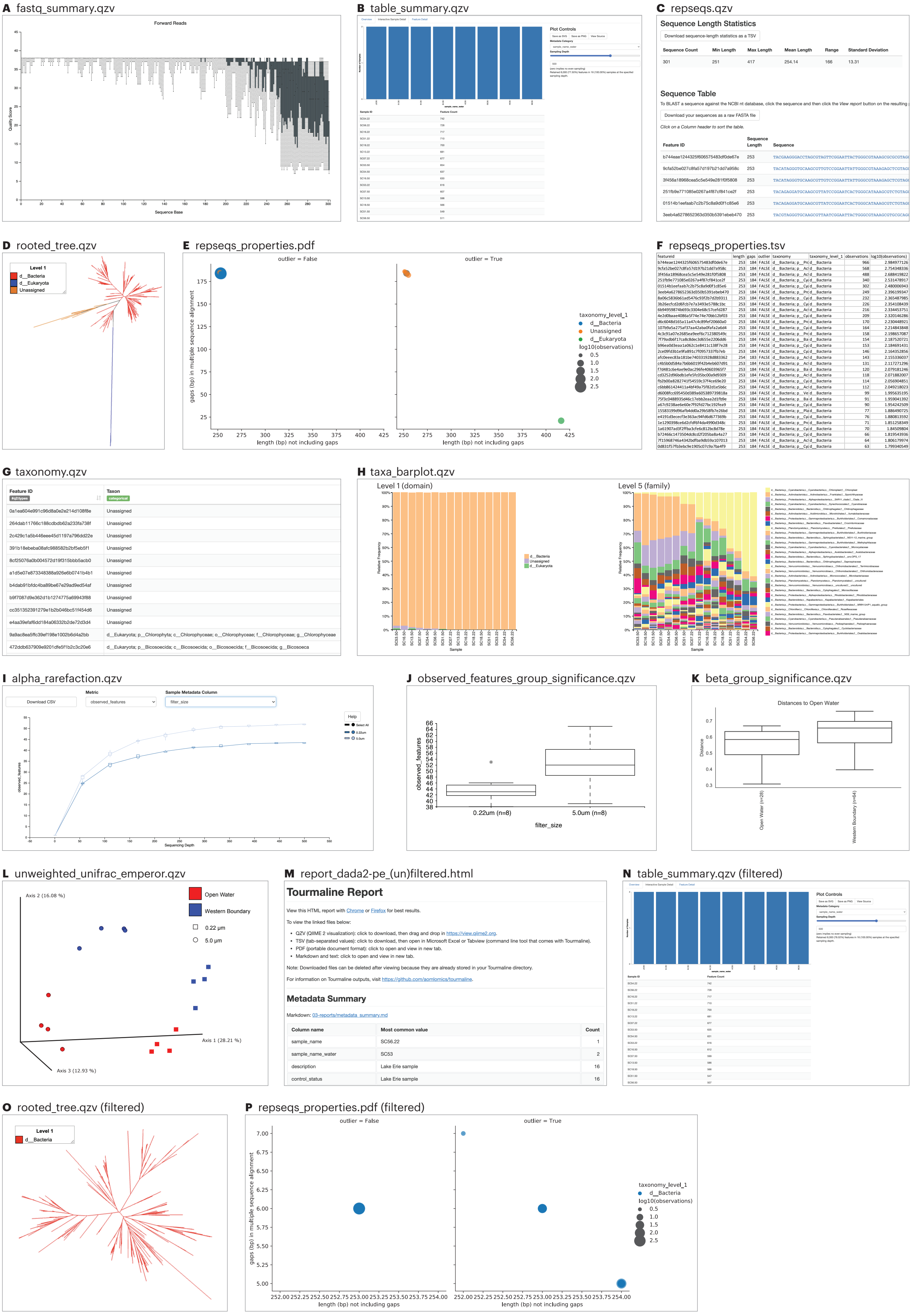

Figure S3. Screenshots of the primary output files after running Tourmaline on the test data (see Fig. 2 for commands, parameters, and guidance). The visualization files (.qzv, .pdf, .html) are useful both for data evaluation and discovery and for biological insight.

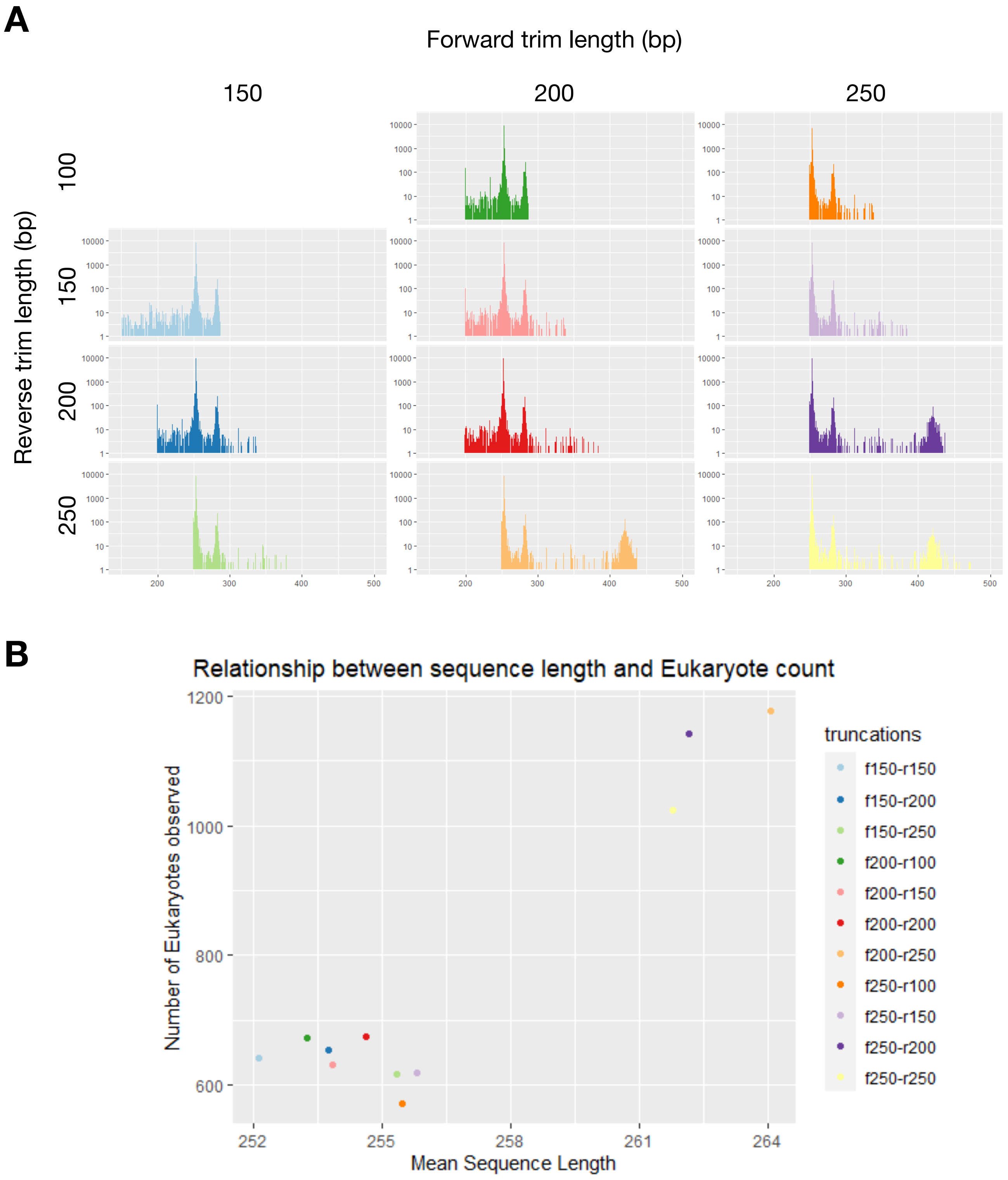

Figure S4. Effect of truncation length parameters on (A) the distribution of representative sequence length and (B) the number of reads assigned to Eukaryota in the full 2018 Lake Erie 16S rRNA amplicon data.
