# Supplementary material for: Tourmaline: A containerized workflow for rapid and iterable amplicon sequence analysis using QIIME 2 and Snakemake
Source: Gigascience. 2022 Jul 28;11:giac066. doi: 10.1093/gigascience/giac066 (PMC9334028; doi:10.1093/gigascience/giac066)
Supplement: giac066_Supplemental_Files [file giac066_supplemental_files.zip › figureS2.pdf]

A

Tourmaline Python Notebook

Package Imports

In [1]:
```
from qiime2 import Artifact
from qiime2 import Visualization
import pandas as pd
import matplotlib.pyplot as plt
import seaborn as sns
%matplotlib inline
```

Variables

In [2]:
```
method = 'dada2-pe'
filtering = 'unfiltered'
adiv_metric = 'observed_features'
bdiv_metric = 'unweighted_unifrac'
factor1='filter_size'
factor2='region'
```

Paths

In [3]:
```
inputs = {
 'metadata': '../00-data/metadata.tsv',
 'taxonomy': '../02-output-%s-%s/01-taxonomy/taxonomy.qza' % (method, filtering),
 'repseq_prop': '../02-output-%s-%s/02-alignment-tree/repseqs_properties.tsv' % (method, filtering),
 'adiv_vector': '../02-output-%s-%s/03-alpha-diversity/%s_vector.qza' % (method, filtering, adiv_metric),
 'repseqs_viz': '../02-output-%s-%s/00-table-repseqs/repseqs.qzv' % (method, filtering),
 'table_viz': '../02-output-%s-%s/00-table-repseqs/table.qzv' % (method, filtering),
 'taxonomy_viz': '../02-output-%s-%s/01-taxonomy/taxonomy.qzv' % (method, filtering),
 'taxa_bar': '../02-output-%s-%s/01-taxonomy/taxa_barplot.qzv' % (method, filtering),
 'rooted_tree': '../02-output-%s-%s/02-alignment-tree/rooted_tree.qzv' % (method, filtering),
 'adiv_grpsig': '../02-output-%s-%s/03-alpha-diversity/%s_group_significance.qzv' % (method, filtering, adiv_metric),
 'bdiv_emperor': '../02-output-%s-%s/04-beta-diversity/%s_emperor.qzv' % (method, filtering, bdiv_metric),
 'bdiv_grpsig': '../02-output-%s-%s/04-beta-diversity/%s_group_significance.qzv' % (method, filtering, bdiv_metric)
}
```

Static Plots

Alpha-diversity boxplots

In [9]:
```
adiv_vector = Artifact.load(inputs['adiv_vector'])
df_adiv = adiv_vector.view(pd.Series)
df_adiv_md = pd.merge(df_md, df_adiv, left_index=True, right_index=True)
sns.boxplot(data=df_adiv_md, y=adiv_metric, x=factor1, hue=factor2)
```

Out[9]: `<matplotlib.axes._subplots.AxesSubplot at 0x7fca71d9ff28>`

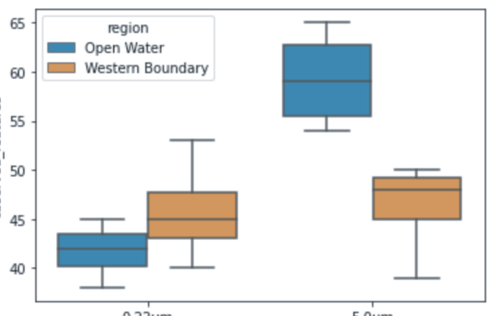

Interactive Visualizations

Rooted tree

In [12]: `Visualization.load(inputs['rooted_tree'])`

Out[12]:

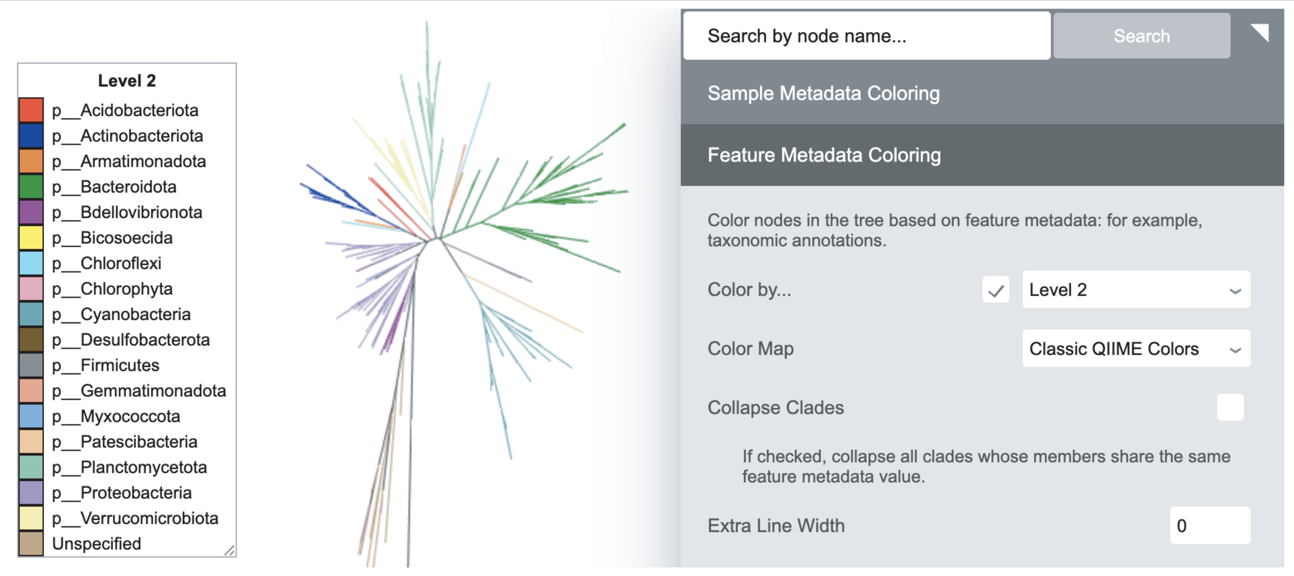

Taxonomy barplot

In [14]: `Visualization.load(inputs['taxa_bar'])`

Out[14]:

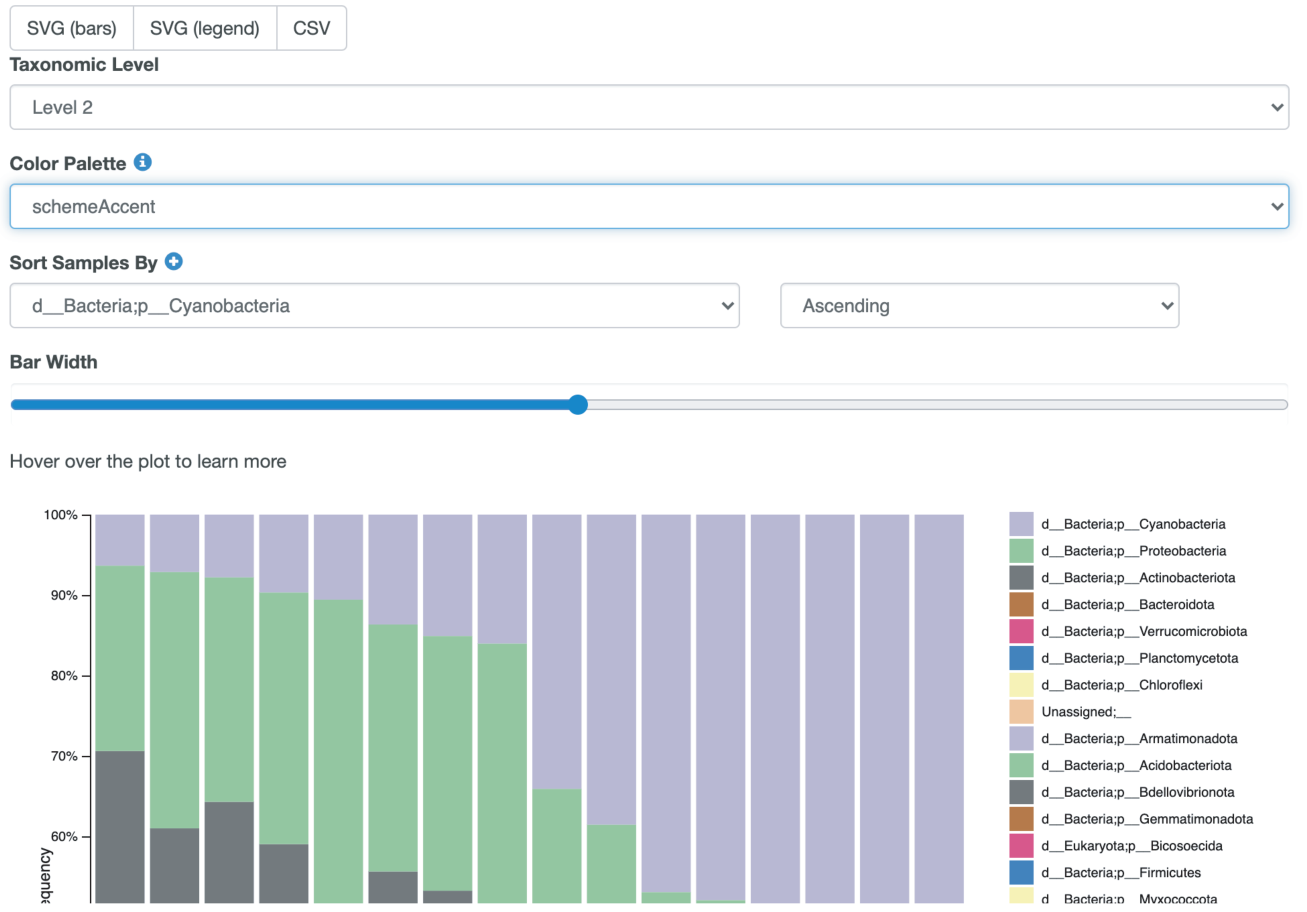

Beta-diversity PCoA Emperor plot

In [16]: `Visualization.load(inputs['bdiv_emperor'])`

Out[16]:

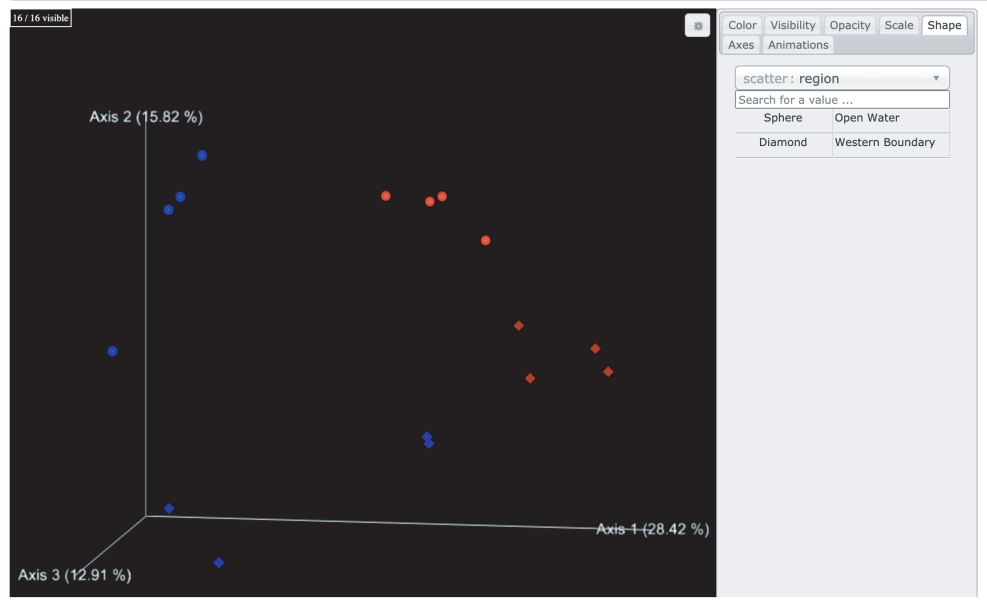

B

Tourmaline R Notebook

Load packages

In [1]:
```
library(qiime2R)
library(phyloseq)
library(tidyverse)
library(RColorBrewer)
library(vegan)
```

Define variables

In [2]:
```
method = "dada2-pe"
filtering = "unfiltered"
adiv_metric = "Shannon"
bdiv_metric = "bray"
factor1 = "region"
factor2 = "filter_size"
factor3 = "sample_name"
```

Read DataFrames

Representative sequences and observation table

In [3]:
```
count_table <- read_qza(file=sprintf("../02-output-%s-%s/00-table-repseqs/table.qza", method, filtering))
count_table <- count_table$data
tax_table <- read_qza(file=sprintf("../02-output-%s-%s/01-taxonomy/taxonomy.qza", method, filtering))
tax_table <- tax_table$data %>%
as_tibble() %>%
separate(Taxon, sep=";", c("Rank1", "Rank2", "Rank3", "Rank4", "Rank5", "Rank6", "Rank7")) # Taxa levels can be changed
```

Metadata

In [4]:
```
metadata_table <- read_tsv(file="../00-data/metadata.tsv")
metadata_table  <- sample_data(metadata_table)
rownames(metadata_table ) <- metadata_table$sample_name
```

Merge into phyloseq object

In [5]:
```
physeq <- phyloseq(otu_table(count_table, taxa_are_rows= T), tax_table(as.data.frame(tax_table) %>% column_to_rownames(

suppressMessages(physeq_rarefy <- rarefy_even_depth(physeq, sample.size = min(sample_sums(physeq)), rngseed = 714, repl
```

Set factors and plotting parameters

In [6]:
```
# Set factors
Factor1 = get(factor1, sample_data(physeq_rarefy))
Factor2 = get(factor2, sample_data(physeq_rarefy))
Factor3 = get(factor3, sample_data(physeq_rarefy))

# Set plotting parameters
theme = theme_bw()
point = geom_point(size = 5)
shape = scale_shape_manual(values = c(21, 24))
boxplot = geom_boxplot(lwd = 1)
color = scale_fill_brewer(palette="Dark2")
bar_color = scale_fill_brewer(palette="Paired")
stacked_bar = geom_bar(stat="identity", position = "fill")
bar_yaxis = scale_y_continuous(expand = c(0, 0), breaks=seq(0,1,0.2), limits=c(0, 1))
y_intercept = geom_hline(yintercept=0)
legend_pos = theme(legend.position="bottom")
legend_row = guides(fill=guide_legend(nrow=2))
plot_image = options(repr.plot.width=8, repr.plot.height=5)
```

Create plots

In [7]:
```
# Estimate richness
alpha <- estimate_richness(
  physeq = physeq_rarefy,
  measures=c(adiv_metric)) %>%
  cbind(Factor1, Factor2)

# Plot
ggplot(data=alpha, aes(x=Factor1, y=Shannon, fill=Factor1)) + theme +
boxplot + color + facet_grid(~ Factor2)
```

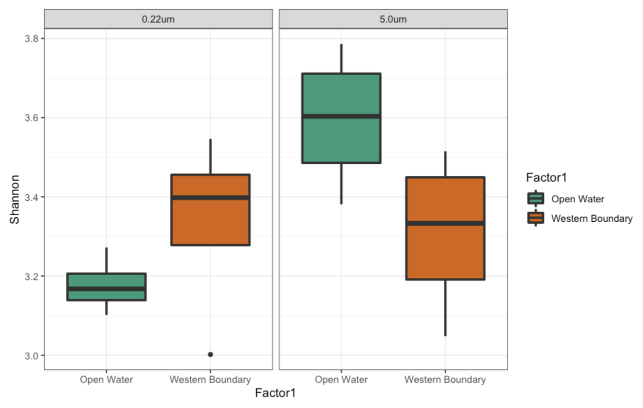

In [8]:
```
# Transform data
physeq_rel <- physeq_rarefy %>%
  transform_sample_counts(function(x) {x/sum(x)} )

# Ordinate
nmds <- ordinate(
  physeq = physeq_rel,
  method = "NMDS",
  distance = bdiv_metric) %>%
  scores() %>%
  as.data.frame() %>%
  cbind(Factor1, Factor2)

# Plot
ggplot(data=nmds, aes(x=NMDS1, y=NMDS2, fill=Factor1, shape=Factor2)) + theme +
color + point + shape + guides(fill=guide_legend(override.aes=list(shape=21)))
```

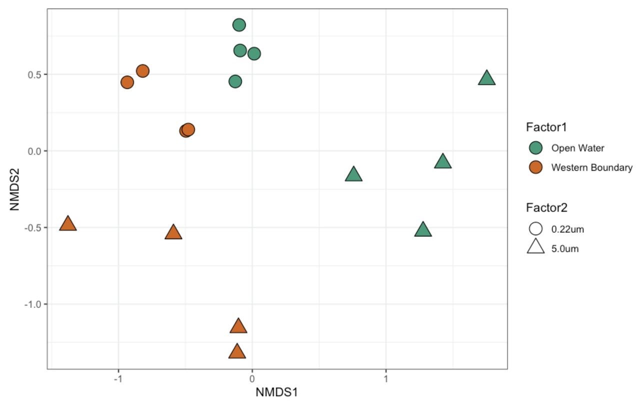

In [9]:
```
# Prepare phyloseq data for bar plots
barplot <- physeq_rarefy %>%
    tax_glom(taxrank = "Rank3") %>%                        # Agglomerate at class level, which is Rank3 in this case
    transform_sample_counts(function(x) {x/sum(x)} ) %>%  # Transform to rel. abundance
    psmelt() %>%                                           # Melt to long format
    cbind(Factor1, Factor2, Factor3) %>%
    filter(Abundance > 0.05) %>%                           # Filter out low abundance taxa
    arrange(Rank3)

# Plot
ggplot(barplot, aes(x=Factor3, y=Abundance, fill=Rank3)) +
stacked_bar + bar_yaxis + y_intercept + bar_color + legend_pos +
legend_row + labs(y = "Relative Abundance") + facet_grid(~ Factor1, scales = "free_x") +
theme(axis.text.x=element_text(angle=90,vjust=0.5))
```

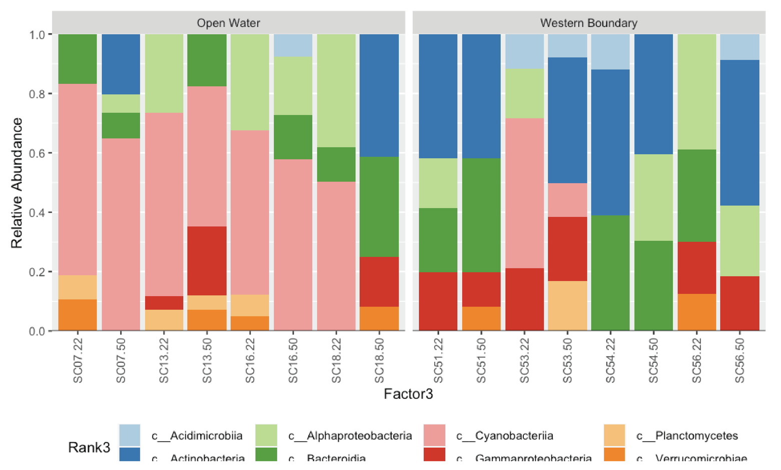

C

Tourmaline Meta-analysis Notebook

This notebook shows how to take Tourmaline outputs from two (or more) separate analyses and merge them for a meta-analysis. The two analyses are derived from the test data that comes with Tourmaline, and the output must first be generated (commands provided).

Import packages

In [ ]:
```
from qiime2 import Artifact
from qiime2 import Visualization
import pandas as pd
import matplotlib.pyplot as plt
import seaborn as sns
%matplotlib inline
```

Set file paths

In [ ]:
```
dir_analysis1 = '../meta-analysis/analysis1'
dir_analysis2 = '../meta-analysis/analysis2'
```

Set up Tourmaline analyses

The below commands will, for each analysis, clone the Tourmaline respository, initialize it using parameters and files from the top-level repository (already downloaded), and copy the provided metadata and manifest files to their proper location in `00-data`.

In [ ]:
```
%%bash
cd ../meta-analysis/analysis1
git clone https://github.com/aomlomics/tourmaline
cd tourmaline
./scripts/initialize_dir_from_existing_tourmaline_dir.sh ../../..
cp ../metadata.tsv 00-data
cp ../manifest_* 00-data
```

In [ ]:
```
%%bash
cd ../meta-analysis/analysis2
git clone https://github.com/aomlomics/tourmaline
cd tourmaline
./scripts/initialize_dir_from_existing_tourmaline_dir.sh ../../..
cp ../metadata.tsv 00-data
cp ../manifest_* 00-data
```

After running the above steps, check that the required input files are all present.

Run Tourmaline analyses

Each commands below will take ~5 minutes to complete. You will not see any output until the command is finished.

In [ ]:
```
%%bash
cd ../meta-analysis/analysis1/tourmaline
snakemake dada2_pe_report_unfiltered
```

In [ ]:
```
%%bash
cd ../meta-analysis/analysis2/tourmaline
snakemake dada2_pe_report_unfiltered
```

Merge outputs

Merge feature tables

Additional feature table files can be merged by adding more `--i-tables` inputs.

In [ ]:
```
%%bash
qiime feature-table merge \
--i-tables ../meta-analysis/analysis1/tourmaline/02-output-dada2-pe-unfiltered/00-table-repseqs/table.qza \
--i-tables ../meta-analysis/analysis2/tourmaline/02-output-dada2-pe-unfiltered/00-table-repseqs/table.qza \
--o-merged-table ../meta-analysis/merged_table.qza
```

Merge representative sequences

Additional representative sequences files can be merged by adding more `--i-data` inputs.

In [ ]:
```
%%bash
qiime feature-table merge-seqs \
--i-data ../meta-analysis/analysis1/tourmaline/02-output-dada2-pe-unfiltered/00-table-repseqs/repseqs.qza \
--i-data ../meta-analysis/analysis2/tourmaline/02-output-dada2-pe-unfiltered/00-table-repseqs/repseqs.qza \
--o-merged-data ../meta-analysis/merged_repseqs.qza
```

Merge taxonomies

Additional taxonomy files can be merged by adding more `--i-data` inputs.

In [ ]:
```
%%bash
qiime feature-table merge-taxa \
--i-data ../meta-analysis/analysis1/tourmaline/02-output-dada2-pe-unfiltered/01-taxonomy/taxonomy.qza \
--i-data ../meta-analysis/analysis2/tourmaline/02-output-dada2-pe-unfiltered/01-taxonomy/taxonomy.qza \
--o-merged-data ../meta-analysis/merged_taxonomy.qza
```

Merge metadata

Additional metadata files can be merged by importing more files and adding them to the `concat` command.

In [ ]:
```
metadata1 = pd.read_csv('%s/tourmaline/00-data/metadata.tsv' % dir_analysis1, sep='\t', index_col=0)
metadata2 = pd.read_csv('%s/tourmaline/00-data/metadata.tsv' % dir_analysis2, sep='\t', index_col=0)
metadata_merged = pd.concat([metadata1, metadata2])
metadata_merged.to_csv('../meta-analysis/merged_metadata.tsv', sep='\t')
```

Analyze merged output

The commands below are some initial examples of analyzing the merged output. A full meta-analysis will go much deeper than this.

Taxonomy barplot

Create a taxonomy barplot.

In [ ]:
```
%%bash
qiime taxa barplot \
--i-table ../meta-analysis/merged_table.qza \
--i-taxonomy ../meta-analysis/merged_taxonomy.qza \
--m-metadata-file ../meta-analysis/merged_metadata.tsv \
--o-visualization ../meta-analysis/merged_taxa_barplot.qzv
```

Alpha diversity

Create an alpha diversity vector using the 'shannon' metric.

In [ ]:
```
%%bash
qiime diversity alpha \
--i-table ../meta-analysis/merged_table.qza \
--p-metric shannon \
--o-alpha-diversity ../meta-analysis/merged_alpha_shannon.qza
```

Beta diversity

Create a beta diversity distance matrix using the 'braycurtis' metric.

In [ ]:
```
%%bash
qiime diversity beta \
--i-table ../meta-analysis/merged_table.qza \
--p-metric braycurtis \
--o-distance-matrix ../meta-analysis/merged_beta_braycurtis.qza
```
