# Supplementary material for: Tourmaline: A containerized workflow for rapid and iterable amplicon sequence analysis using QIIME 2 and Snakemake
Source: Gigascience. 2022 Jul 28;11:giac066. doi: 10.1093/gigascience/giac066 (PMC9334028; doi:10.1093/gigascience/giac066)
Supplement: giac066_Supplemental_Files [file giac066_supplemental_files.zip › figureS3.pdf]

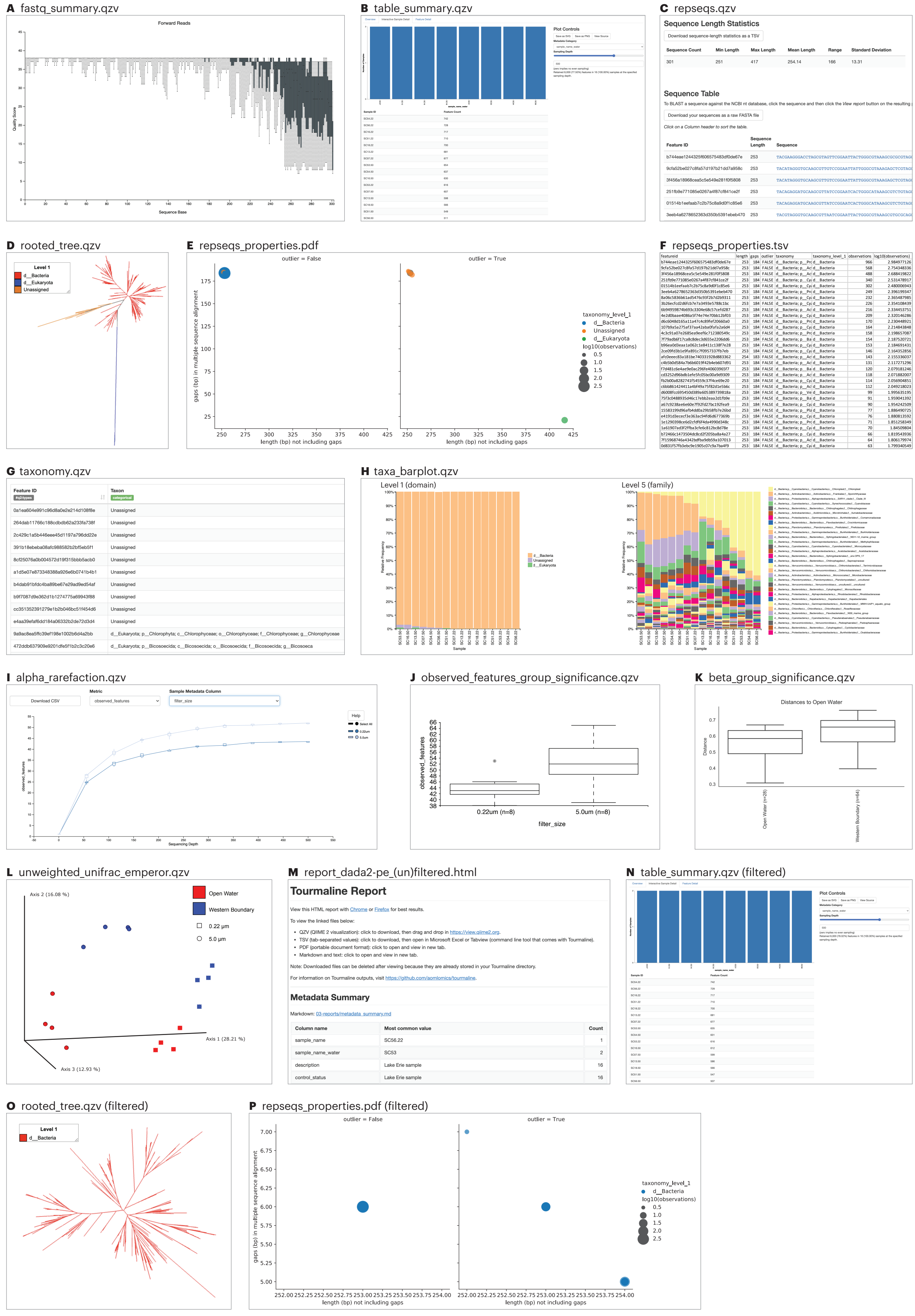

A fastq_summary.qzv
Forward Reads
Quality Score
Sequence Base
B table_summary.qzv
Plot Controls
Sample ID
Feature Count
C repseqs.qzv
Sequence Length Statistics
Download sequence-length statistics as a TSV
Sequence Count
Min Length
Max Length
Mean Length
Range
Standard Deviation
301
251
417
254.14
166
13.31
Sequence Table
Download your sequences as a raw FASTA file
Click on a Column header to sort the table.
Feature ID
Sequence Length
Sequence
b744eae1244325f606575483df0de67e
9cfa52be027c8fa57d197b21dd7a958c
3f456a18968cea5c5e549e281f0f5808
251fb9e771085e0267a4f87cf841ce2f
01514b1eefaab7c2b75c8a9d0f1c85e6
3eeb4a6278652363d350b5391ebeb470
D rooted_tree.qzv
Level 1
d__Bacteria
d__Eukaryota
Unassigned
E repseqs_properties.pdf
outlier = False
outlier = True
gaps (bp) in multiple sequence alignment
length (bp) not including gaps
taxonomy_level_1
d__Bacteria
Unassigned
d__Eukaryota
log10(observations)
F repseqs_properties.tsv
featureid
length
gaps
outlier
taxonomy
taxonomy_level_1
observations
log10(observations)
G taxonomy.qzv
Feature ID
Taxon
Unassigned
d__Eukaryota; p__Chlorophyta; c__Chlorophyceae; o__Chlorophyceae; f__Chlorophyceae; g__Chlorophyceae
d__Eukaryota; p__Bicosoecida; c__Bicosoecida; o__Bicosoecida; f__Bicosoecida; g__Bicosoecida
H taxa_barplot.qzv
Level 1 (domain)
Level 5 (family)
Relative Frequency
Sample
I alpha_rarefaction.qzv
Download CSV
Metric
observed_features
Sample Metadata Column
filter_size
Help
observed_features
Sequencing Depth
J observed_features_group_significance.qzv
observed_features
0.22um (n=8)
5.0um (n=8)
filter_size
K beta_group_significance.qzv
Distances to Open Water
Distance
Open Water (n=28)
Western Boundary (n=64)
L unweighted_unifrac_emperor.qzv
Axis 2 (16.08 %)
Axis 1 (28.21 %)
Axis 3 (12.93 %)
Open Water
Western Boundary
0.22 µm
5.0 µm
M report_dada2-pe_(un)filtered.html
Tourmaline Report
View this HTML report with Chrome or Firefox for best results.
To view the linked files below:
QZV (QIIME 2 visualization): click to download, then drag and drop in https://view.qiime2.org.
TSV (tab-separated values): click to download, then open in Microsoft Excel or Tabview (command line tool that comes with Tourmaline).
PDF (portable document format): click to open and view in new tab.
Markdown and text: click to open and view in new tab.
Note: Downloaded files can be deleted after viewing because they are already stored in your Tourmaline directory.
For information on Tourmaline outputs, visit https://github.com/aomlomics/tourmaline.
Metadata Summary
Markdown: 03-reports/metadata_summary.md
Column name
Most common value
Count
sample_name
SC56.22
1
sample_name_water
SC53
2
description
Lake Erie sample
16
control_status
Lake Erie sample
16
N table_summary.qzv (filtered)
Plot Controls
O rooted_tree.qzv (filtered)
Level 1
d__Bacteria
P repseqs_properties.pdf (filtered)
outlier = False
outlier = True
gaps (bp) in multiple sequence alignment
length (bp) not including gaps
taxonomy_level_1
d__Bacteria
log10(observations)
