# Supplementary material for: Tourmaline: A containerized workflow for rapid and iterable amplicon sequence analysis using QIIME 2 and Snakemake
Source: Gigascience. 2022 Jul 28;11:giac066. doi: 10.1093/gigascience/giac066 (PMC9334028; doi:10.1093/gigascience/giac066)
Supplement: giac066_Supplemental_Files [file giac066_supplemental_files.zip › figureS4.pdf]

A

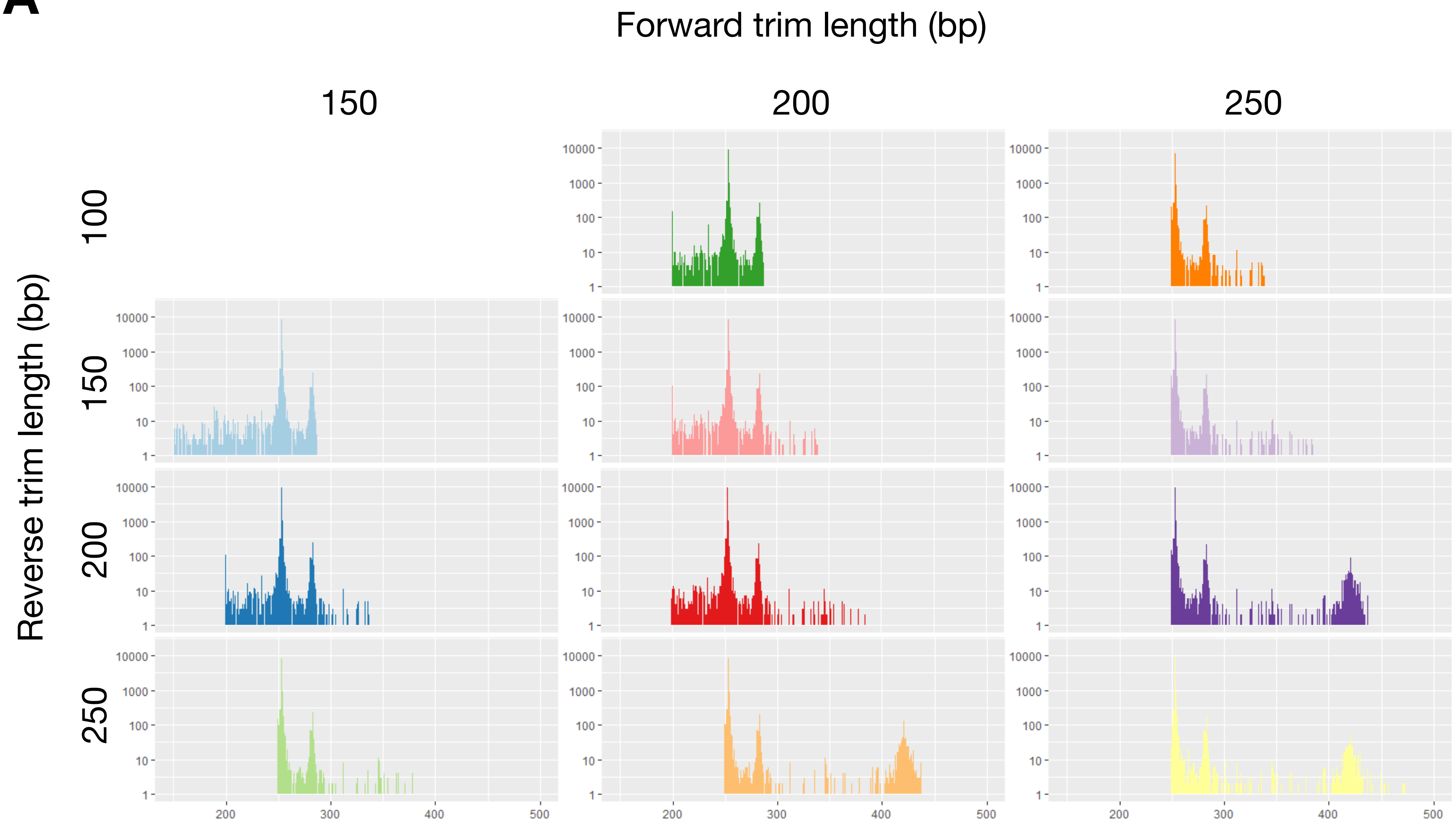
Forward trim length (bp)
150
200
250
Reverse trim length (bp)
100
150
200
250
10000
1000
100
10
1
200
300
400
500

B

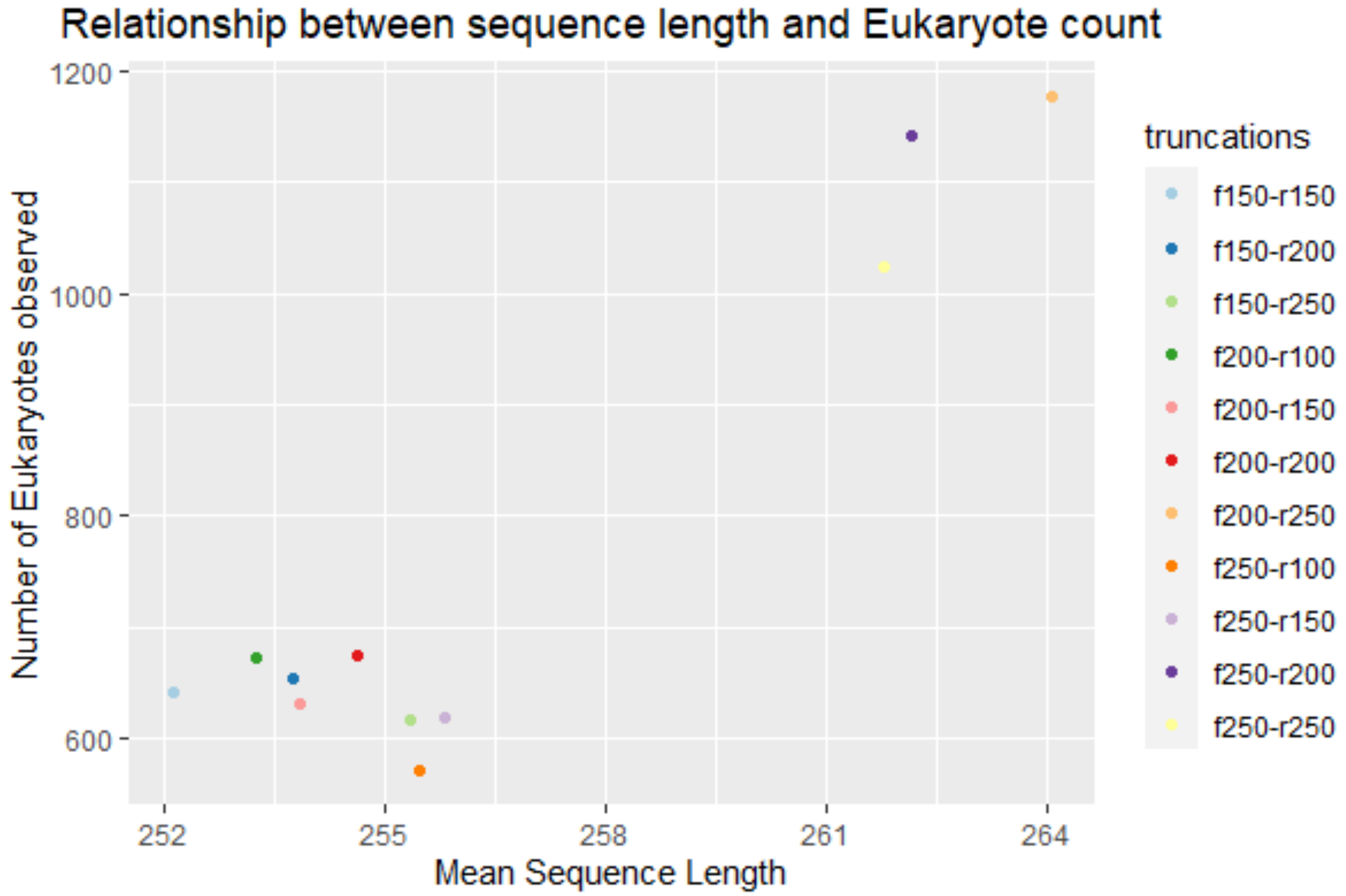
Relationship between sequence length and Eukaryote count
Number of Eukaryotes observed
1200
1000
800
600
252
255
258
261
264
Mean Sequence Length
truncations
f150-r150
f150-r200
f150-r250
f200-r100
f200-r150
f200-r200
f200-r250
f250-r100
f250-r150
f250-r200
f250-r250
