# Supplementary material for: Tourmaline: A containerized workflow for rapid and iterable amplicon sequence analysis using QIIME 2 and Snakemake
Source: Gigascience. 2022 Jul 28;11:giac066. doi: 10.1093/gigascience/giac066 (PMC9334028; doi:10.1093/gigascience/giac066)
Supplement: giac066_Supplemental_Files [file giac066_supplemental_files.zip › tableS1.pdf]

Parameter	Description	Recommendation	Help
dada2pe_trunc_len_f<br>dada2pe_trunc_len_r<br>dada2se_trunc_len	Truncate bases (integer) from the 3' (right) ends of reads in DADA2.	Choose values that maximize length but remove low-quality ends. Note that DADA2 paired-end mode requires a minimum overlap of 12 bp to merge Read 1 and Read 2. See the section below "Sequence quality control and choice of truncation length" for instructions on using the included script fastqc_per_base_sequence_quality_dropoff.py.	dada2 denoise-paired; dada2 denoise-single
dada2pe_trim_left_f<br>dada2pe_trim_left_f<br>dada2se_trim_left	Trim bases (integer) from the 5' (left) ends of reads in DADA2.	Depending on your amplicon sequencing method, and if trimming was not done prior to running Tourmaline, you may have primer sequences, indexes, and/or adapters on the 5' ends of your reads. If so, set this parameter to remove those bases. If not, set this parameter to zero. Note that 5' trimming (this parameter) is done after 3' truncation (above parameter).	dada2 denoise-paired; dada2 denoise-single
deblur_trim_length	Truncate bases (integer) from the 3' (right) ends of reads in Deblur.	Choose values that maximize length but remove low-quality ends. See the section below "Sequence quality control and choice of truncation length" for instructions on using the included script fastqc_per_base_sequence_quality_dropoff.py.	deblur denoise-other
dada2pe_pooling_method<br>dada2se_pooling_method	DADA2 pooling method.	Choose pseudo for pseudo-pooling or independent for no pooling.	dada2 denoise-paired; dada2 denoise-single
dada2pe_chimera_method<br>dada2se_chimera_method	DADA2 chimera method.	Choose pooled if pseudo-pooling otherwise consensus or none.	dada2 denoise-paired; dada2 denoise-single
alignment_method	Multiple sequence alignment method.	Choose muscle or clustalo for best accuracy or mafft for faster results.	muscle; clustalo; mafft
classify_method	Taxonomic classification method.	Choose naive-bayes for best accuracy or consensus-blast for faster results.	feature-classifier
exclude_terms	Filter terms (taxa) from taxonomy.	Specify terms (comma-separated, no spaces) to find in taxonomy and filter out (case-insensitive), or provide a nonsense term to skip this step when filtering.	taxa filter-seqs
repseq_min_length<br>repseq_max_length	Set minimum and maximum sequence lengths to filter representative sequences by.	Limits are inclusive, i.e., sequences will be retained if greater than or equal to minimum, less than or equal to maximum. Leave defaults (0, 10000) to do no filtering.	taxa filter-seqs
repseq_min_abundance<br>repseq_min_prevalence	set minimum abundance and prevalence limits to filter representative sequences by.	Limit is inclusive, i.e., sequences will be retained if greater than or equal to minimum. Leave default (0) to do no filtering.	taxa filter-seqs
odseq_distance_metric	Distance metric for odseq.	Choose metric from: linear, affine.	odseq
odseq_bootstrap_replicates	Number (integer) of bootstrap replicates for odseq.	Choose more replicates for more robust detection of outliers, fewer replicates for faster processing.	odseq
odseq_threshold	Threshold (float) for bootstrap probability distribution for odseq.	Probability to be at the right of the bootstrap scores distribution when computing outliers. Tune this parameter depending on the diversity and occurrence of outliers in the MSA.	odseq
core_sampling_depth	Rarefaction depth (integer) for core diversity metrics.	Choose a value that balances sequencing depth (more is better) with number of samples retained (more is better).	diversity core-metrics-phylogenetic
alpha_max_depth	Rarefaction depth (integer) for alpha rarefaction.	Choose a value that balances sequencing depth (more is better) with number of samples retained (more is better).	diversity alpha-rarefaction
beta_group_column	Column (text) in your metadata to test beta-diversity group significance.	Choose a category that may differentiate your samples. This analysis can be rerun with different columns by renaming the output file and changing the value in config.yaml before running again.	diversity beta-group-significance
report_theme	HTML report theme.	Choose from: github, gothic, newsprint, night, pixyll, whitey.	Typora theme gallery
