# Supplementary figures and images for: Tourmaline: A containerized workflow for rapid and iterable amplicon sequence analysis using QIIME 2 and Snakemake
Source: Gigascience. 2022 Jul 28;11:giac066. doi: 10.1093/gigascience/giac066 (PMC9334028; doi:10.1093/gigascience/giac066)

A

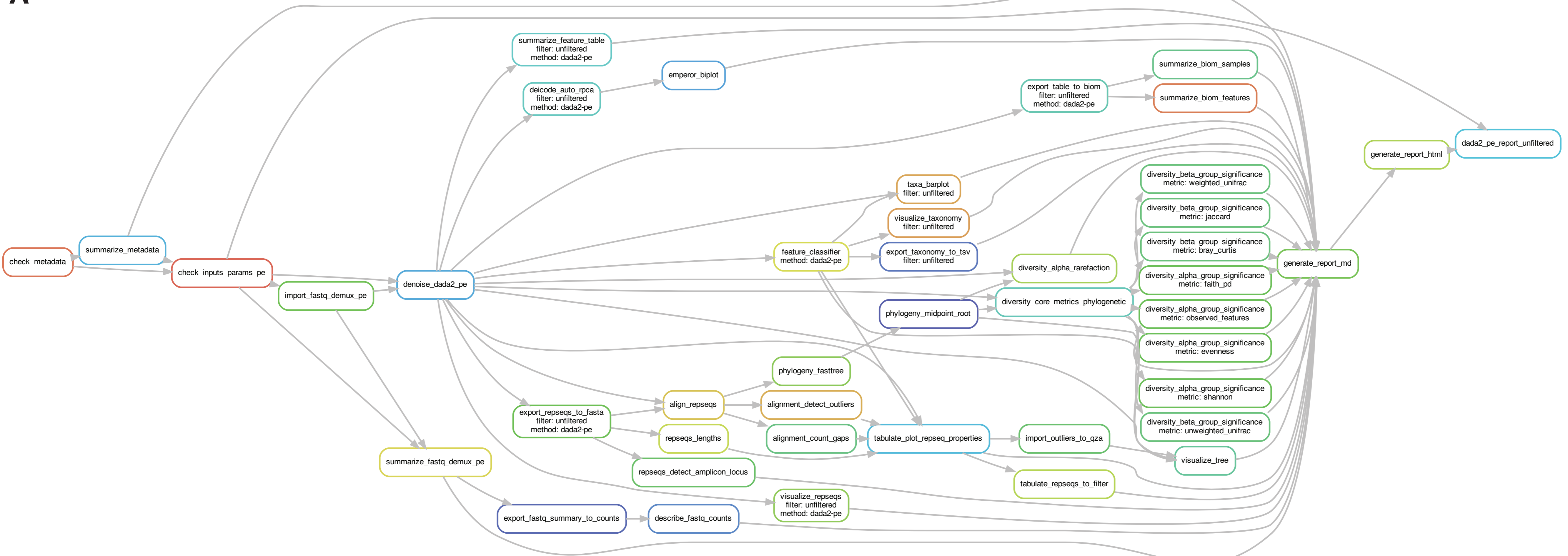

B

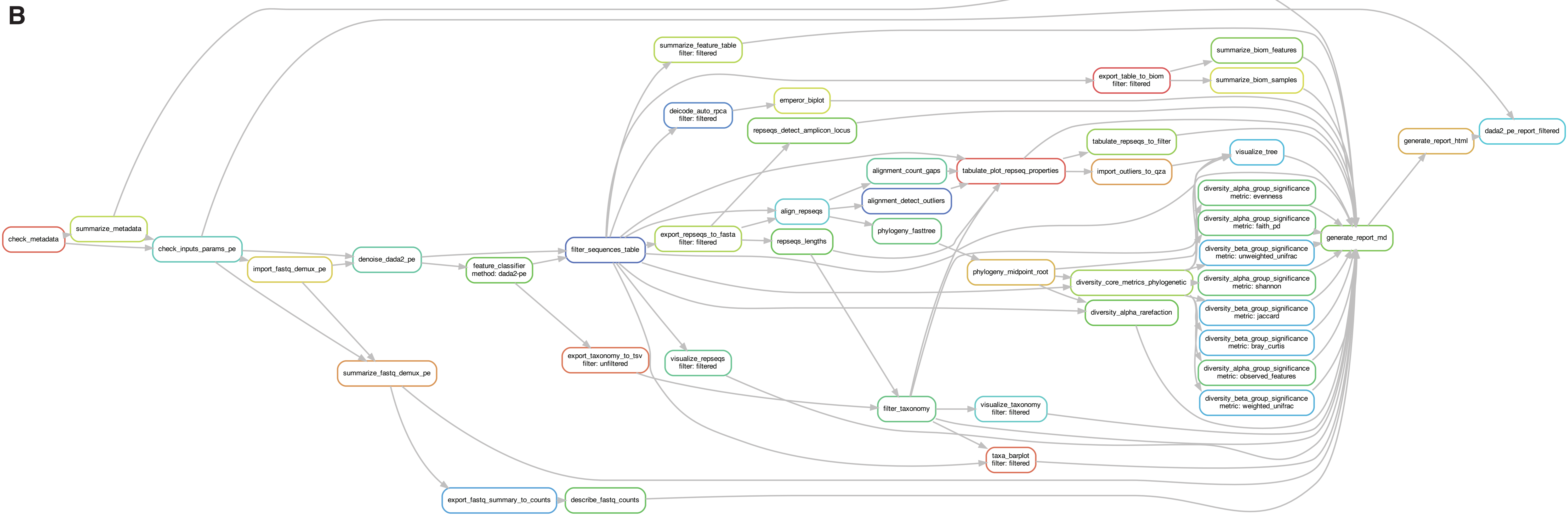

Supplement: giac066_Supplemental_Files [file giac066_supplemental_files.zip › figureS1.pdf]
