# Supplementary material for: Tourmaline: A containerized workflow for rapid and iterable amplicon sequence analysis using QIIME 2 and Snakemake
Source: Gigascience. 2022 Jul 28;11:giac066. doi: 10.1093/gigascience/giac066 (PMC9334028; doi:10.1093/gigascience/giac066)
Supplement: giac066_Response_to_Reviewer_Comments_Original_Submission [file giac066_response_to_reviewer_comments_original_submission.pdf]

Author's Response To Reviewer Comments

Close

Point-by-point Response

Reviewer #1

Thompson et al. present a workflow for amplicon sequencing analysis, wrapping commands from the commonly used QIIME2 package in the commonly used workflow manager Snakemake. The manuscript is clearly structured and contains figures of appropriate quality. Building and testing user-friendly workflows that facilitate the use of existing software is an important task for the research community. The chosen existing softwares (namely Snakemake as workflow manager and QIIME2's calls to DADA2 and deblur, as well as QIIME2's visualisations) are trusted and often used by the community. Next to manuscript, I have inspected the GitHub/wiki page of the proposed workflow and tested it on the provided test data as well as an independent data set. I have run into some issues, which I will put forward below, together with some comments on the content of and omissions from the manuscript.

We thank the reviewer for the comprehensive evaluation of our workflow and accompanying manuscript. Their comments have been very helpful in improving the tool and revising the manuscript, as we describe below in our response.

Manuscript:

1. Overall, the reads more like a manual or tutorial than a methods description. The point-by-point description of the outputs may be a bit lengthy.

> We thank the reviewer for this candid critique and have condensed the
> instructions and description of outputs and referred the reader to the
> GitHub Wiki for more detail.

2. The manuscript is missing information on runtimes and hardware requirements. This is in particular a pity because the workflow does not make use of parallelisation of the called tools. It might be pretty slow on large datasets?

> We did not highlight in the original manuscript that the workflow does
> indeed support parallelization of individual commands using the
> Snakefile. We have now added a section on benchmarking and
> parallelization, including runtimes for a medium-sized dataset with
> and without parallelization. The runtime for a typical dataset (96
> samples with ~120,000 reads per sample) was 5 hours with a single
> core but decreased to 2 hours with 8 cores.

3. There is also no justification for the choices of the analyses that are done and the defaults that were chosen.

> We thank the reviewer for pointing out the lack of justification for
> the choices made regarding commands and default parameters. We have
> added justification for these choices, noting that the commands chosen
> encompass analyses commonly used by microbiome and eDNA researchers,

> and that parameters were chosen to balance run performance and
> accuracy and to work with the test data.

4. In the introduction, other published amplicon sequencing workflows are cited and dismissed as not all well documented. Other than maybe not being so well documented, there are differences in scope between these workflows and the one described here. It would be very helpful for readers to be informed on how the described workflow is set apart from those workflows. And also from QIIME2 (all of the images in figure 3, for example, are QIIME2's visualisation work and not part of the workflow's report).
Finally, tagseq, which also wraps QIIME2 commands in snakemake, is not mentioned. From my point of view, the workflow still requires the user to be able to do quite a lot of data setup in the command line environment and requires knowledge of QIIME2, while resolving relatively little by wrapping the commands in Snakemake (also see my next point). Clearly, it would be helpful to discuss what existing problem the workflow overcomes that the others (and QIIME2) don't.

> We acknowledge that we were unfairly dismissive of other workflows in
> the original manuscript. We have now included a more comprehensive
> list of other workflows in the introduction that might be used instead
> of Tourmaline, including tagseq, CoMA, and ASAP 2. We have also
> highlighted throughout the manuscript what Tourmaline provides that
> makes it unique and potentially a good option for some users,
> specifically: 1- the additional summary plots and reports (which are
> now highlighted rather than the QIIME 2 visualizations), 2- the Docker
> container containing all the dependencies, 3- Snakemake features
> (single config file, reproducible directory structure, and on-demand
> commands), 4- Jupyter notebooks ready to load and analyze Tourmaline
> outputs, and 5- a well-documented wiki with step-by-step instructions.

5. In the same paragraph of the introduction, it is mentioned that the workflow might evolve with QIIME2. However, it makes use of only a small part of QIIME's options/commands - is there a plan to widen the scope? How will continuous support be done? Is there a plan to integrate the workflow into the QIIME software ecosystem?
Similarly, the workflow is not using Snakemake to its full potential, e.g. it does require several manual installation steps instead of making use of Snakemake's conda integration; it doesn't make use of Snakemake's reporting ability, which might be interesting together with QIIME2's data provenance. So, is there a plan to improve this? Also for developing it towards better usability on (cloud) cluster structures?

> In its current implementation, Tourmaline provides a core workflow
> with many of the most popular commands provided as rules (see answer
> above to your point #3). We continue to add more QIIME 2 commands
> since the original submission, such as DEICODE (Martino et al. 2019),
> which is a tool that performs Aitchison transformation of amplicon
> data and allows for users to visualize community composition of
> inherently sparse datasets, and is now part of the Tourmaline
> workflow. We expect to continue adding the most useful commands. We
> have shared Tourmaline in the QIIME 2 Forum and have received positive
> feedback from the QIIME 2 user community, and we hope that, with the
> publication of this manuscript, this community will contribute to the
> workflow, possibly with new forks that implement more commands.
> Notably, we have received funding to deploy Tourmaline on the Amazon
> cloud. To better explain our vision for Tourmaline, we have closed the
> conclusion thusly: "Future improvements to the workflow will include
> support for new QIIME 2 releases and plugins, better integration with

> Snakemake, possibly including Conda integration and connecting
> Snakemake's reporting ability with QIIME 2's provenance tracking, and
> enhanced support for cloud computing environments. With its existing
> features that balance usability, functionality, iterability, and
> scalability, and with continued development with support from the
> research community, Tourmaline will be a valuable and longstanding
> tool for amplicon sequence analysis."

Testruns:

What worked:

- Overall, I could install the software on a linux machine by following the description on the GitHub webpage. The test run worked as expected. The data was accessible and I could visualise it using the QIIME2 online viewer. I could run the workflow on an unrelated dataset.

> We are glad that you could successfully install the software, run the
> test data, and visualize the outputs. It is also good to hear you were
> successful applying Tourmaline to an unrelated dataset.

What could be improved:

- The setup of the input was a bit annoying, because the names and paths to the inputs and outputs need to be set at various places. The fact that existing and non-existing inputs have to be defined in the config confused me at first. The error messages that ensued from not doing this right were uninformative (these cases could be caught by the Snakefile with or without the help of a scheme).

> We have simplified the input files by requiring that they be named (or
> have symbolic links) to match the defaults. Therefore the user does
> not need to set these parameters, and the workflow will automatically
> generate the inputs/outputs as required. We have added a rule that
> checks, at the beginning of the workflow, if the required input files
> (and which version of them) are provided.

- While the workflow is very well documented, the settings for the individual denoising / taxonomy steps are not. The links to the QIIME2 documentation don't point to the current version.

> We have added additional guidance in the config file, including help
> commands and links that describe the underlying commands being run,
> especially for the denoising, taxonomy, alignment, and filtering
> steps. It is normal behavior for the links to the QIIME 2
> documentation to point to an older version of the documentation,
> because we are using an older version of QIIME 2 (due to dependencies
> that do not support the latest versions of QIIME 2 and its use of
> Python 3.8).

What didn't work:

- Running a small dataset - the workflow expects to be able to do statistical test with groups and replicates. However, only a late step checks if the data set is suitable, so there is a failure after considerable running time, which is annoying. While this kind of analysis may be the most common application, it's not the only one. It would be good if those parts of the workflow that require certain dataset structures could be switched off.

> We thank the reviewer for pointing out this snag in the workflow.

> There is a parameter in config.yaml called "beta_group_column" and by
> default it is set to "region". This should work with the test data but
> will fail with another dataset unless it happens to contain a metadata
> column called "region". This parameter is not required for initial
> steps, only when the diversity rule is run, and if it fails and the
> rule has to be re-run, only the commands that need to be rerun will be
> run. We have added a note to config.yaml to notify the user that "this
> column must appear in the metadata file (eg, 00-data/metadata.tsv)".
> We have also added a rule that checks, at the beginning of the
> workflow, if this parameter is in the metadata file and will throw an
> error if not.

minor:

- As a very irregular user of QIIME2, I find the QIIME2-jargon
difficult to understand (e.g. artefacts and artefact equivalents,
manifesto, and the QIIME2 names of the DADA2 and deblur steps,
emperor plot...). It would be better, if these were defined (and
maybe not all discussed in detail).

> We have avoided using jargon wherever possible while still using the
> appropriate terminology. We have described the steps in more detail in
> the wiki. As this is a QIIME 2-based workflow, some knowledge of QIIME
> 2 jargon and data structures is unavoidable.

- Personally, I would like to have a primer removal step in the
workflow. But that's a design decision that can be discussed.

> We agree that primer removal is important to consider for any amplicon
> workflow. We did not include primer removal or quality filtering
> because these can be an iterative process and will vary across
> projects and applications. It can be dangerous to have a single
> removal step, without user modification. We have included text in the wiki
> (https://github.com/aomlomics/tourmaline/wiki/3-Setup#remove-primers-if-necessary)
> with code and steps to remove primers in QIIME 2 using the cutadapt
> plugin. Users can control primer removal settings and cutadapt can be
> performed in the same QIIME 2 environment. Trimmed reads are then
> easily passed along to Tourmaline.

- "the fungal internal transcribed spacer (ITS) of the rRNA gene
(Abarenkov et al. 2010)" - the internal transcribed spacer is not
within an rRNA gene, but the different ITS regions are found
between rRNA genes.

> We have clarified this distinction in the introduction.

Reviewer #2

Dear authors,

First of all, my apologies for the delay in the submission of the
review.
I appreciated the opportunity to review your manuscript. Tourmaline aims
at facilitating an easy-to-follow architecture for tracking input and
output file names, parameters, and commands of QIIME2 runs to enhance
meta-analyses. If I am not mistaken, this is the corner-stone of this
study so my review is based on that.

Running Tourmaline is straightforward and its documentation is
exceptional. The video tutorial and the GitHub wiki
(https://github.com/aomlomics/tourmaline/wiki) allows non-experienced
users to start working their analysis and the containerized version of

the tool allows an easy-to-go installation in multiple operating systems without extra effort. The extra visual components provide insight in a nice way and the report returned can provide added value on the runs.

However, even if I do share the authors' interest on usability and interoperability and tools could have a great impact in the community indeed, Tourmaline currently lacks any substantial features to be considered as a stand-alone software tool. In addition, there are several issues that it is my belief that need to be addressed (see the following list).

> We thank the reviewer for the thorough review. In our revisions we have
> highlighted the features of Tourmaline that set it apart from other
> amplicon workflows. We believe that it will be a useful tool for a
> certain group of users. Features that make Tourmaline unique include: 1-
> the additional summary plots and reports (which are now highlighted
> rather than the QIIME 2 visualizations), 2- the Docker container
> containing all the dependencies, 3- Snakemake features (single config
> file, reproducible directory structure, and on-demand commands), 4-
> Jupyter notebooks ready to load and analyze Tourmaline outputs, and 5- a
> well-documented wiki with step-by-step instructions.

Major issues

Major Issue #1: The authors claim that "this lack of automation and standardization [in tracking input and output file names, parameters, and commands on QIIME2] is inefficient and creates barriers to meta-analysis and sharing of results. Therefore, what Tourmaline and thus, the manuscript needs to demonstrate, is that meta-analyses are now feasible to a greater extent, thanks to the Tourmaline wrapper.

> We thank the reviewer for providing the opportunity to better highlight
> the capacity of Tourmaline to facilitate meta-analysis. We have now
> provided a section on how this works in practice and have added a new
> Jupyter notebook that walks through merging two sets of Tourmaline
> outputs and performing some basic diversity analyses on the merged
> output.

Major Issue #2: Assuming that enhancing meta-analyses is the main contribution of Tourmaline, it is fundamental to consider the minimum information about a marker gene sequence (MIMARKS) standard of the Genomic Standards Consortium (GSC). Rather than just mentioning MIMARKS, Tourmaline needs to explore ways to exploit such standards, i.e. perhaps by adding MIMARKS columns in the config file.

> We agree that including support for MIxS/MIMARKS is important as we move
> as a community toward more standardization. As suggested, we have added
> guidance to the config file about using MIxS/MIMARKS metadata fields and
> have made the metadata that comes with Tourmaline fully
> MIMARKS-compliant.

Major Issue #3: As QIIME2 has been developed on the basis of a plugin architecture, it would be highly recommended that such an application could be provided as a plugin too, joining the corresponding QIIME2 library (https://library.qiime2.org/plugins/).

> We have reached out to the QIIME 2 community via the QIIME 2 Forum, and
> have received positive feedback. Our hope is that this community will
> consider contributing to the workflow codebase, for example by
> submitting pull requests, especially after the manuscript is published.
> A workflow such as this one would, however, not be appropriate to be a
> plugin, as plugins function as QIIME 2 commands, whereas this workflow

> wraps many commands. We certainly plan to continue integrating new
> plugins/commands into Tourmaline as they are added to QIIME 2.

Major Issue #4: With respect to the structure of the manuscript, it is my belief that there are sections that should be omitted. Tutorials and "how to" are of extremely valuable but it would be better to be provided either as supplementary material or through repositories, e.g. GitHub wiki, GitHub pages etc, rather than in the main manuscript. The wiki page on Tourmaline's GitHub repository is rather informative. An alternative might be merging the "Overview" along with the "Snakefile", "Config file", "Input files" and "Run the workflow" sections, to describe "The Tourmaline workflow" architecture in a less verbose way, highlighting the role of the "Snakefile" and the "config.yml" files and the architecture that binds them together. "Documentation", "Installation", "Cloning" subsections could/should be omitted too.

> We agree and thank the reviewer for this suggested restructuring. We
> have condensed the instructions and description of outputs and referred
> the reader to the GitHub Wiki for more detail.

Major Issue #5: The test dataset does not allow the validation of Tourmaline in meta-analyses. It is rather important to have a testbed dataset to demonstrate "how to run" but a use case of an actual meta-analysis is required to demonstrate how different analyses can be combined in the framework of Tourmaline and provide further insight than those of the initial ones.

> This is a great suggestion, and we have followed this advice. We have
> added a Jupyter notebook that performs merging of two (or more) sets of
> Tourmaline outputs and described how this works in the manuscript.

Major Issue #7: No license has been included in the "Availability of supporting source code" section. On Tourmaline GitHub repo a license (https://github.com/aomlomics/tourmaline#license) is mentioned, yet GigaScience asks for an appropriate Open Source Initiative compliant license (https://opensource.org/licenses/category). In addition, I tried to find if the QIIME2 license is mentioned in a Tourmaline Docker container and I could not; if I am not mistaken that is required based on the QIIME2 license (https://github.com/qiime2/qiime2/blob/master/LICENSE). My apologies again in case of any misapprehension.

> We have updated the license on GitHub to a 3-clause BSD license. As
> recommended by the QIIME 2 lead developer, we have added text to the
> DockerHub page for the Tourmaline Docker image stating, "This docker
> image is based on a QIIME 2 Docker image hosted at
> https://quay.io/repository/qiime2/core?tag=2021.2. The QIIME 2 license
> can be found at https://github.com/qiime2/qiime2/blob/master/LICENSE."
> We have added both of these pieces of information to the section
> "Availability of supporting source code".

Major Issue #8: Parameter optimization is indeed one of the greatest challenges in metabarcoding bioinformatics analyses. However, it is not clear to me how by keeping the exact same names in your output files, will you be able to compare the results of the different runs.

> This can be done by creating copies of the entire Tourmaline directory
> using the helper script. Then one or more parameters can be changed in
> the copies to see how these changes affect the results. We have made
> this clearer in the revised manuscript and added a section on parameter
> optimization.

Major Issue #9: I realise that the authors provide Figures 2 and 3 in a complementary way, presenting the visual component returned after each step. However, having a figure with 16 screenshots makes it hard for the reader to realize what is coming from QIIME2 and what from Tourmaline but most importantly does not highlight the added value that Tourmaline provides to such an analysis. It is my belief that Figure 2 could remain as it, while Figure 3 should focus on the output components that are not provided by QIIME2 routines, but from Tourmaline functionalities. In case of a meta-analysis, this figure should highlight all the added value that using QIIME2 through the Tourmaline wrapper would provide.

> We agree these figures were too busy and have simplified them. We thank
> the reviewer for the suggestion to focus on the parts of the workflow
> that are specific to Tourmaline and have revised the figure accordingly.

Minor issues

Minor Issue #1: Please rephrase the Findings section in the abstract, so that it is clear that Tourmaline invokes QIIME2 routines to implement taxonomy assignment, perform analysis etc. It is required to state clearly what QIIME2 does and what are the extra features of Tourmaline throughout the manuscript.

> We have rephrased the abstract to make clearer the relationship between
> Tourmaline and QIIME 2, and have striven to make this distinction clear
> throughout the manuscript. Notably, Fig. 3 is now focused on outputs
> unique to the Tourmaline workflow.

Minor Issue #2: The conclusion you mention in the abstract is not in line with the scope of Tourmaline that was described earlier. Tourmaline does not accelerate the performance of QIIME2 routines. Its aim, as mentioned earlier, is to enhance meta-analysis and sharing of results.

> We believe that this wording is appropriate for a couple of reasons.
> First, Tourmaline does provide speed improvements by supporting parallel
> processing. More importantly, Tourmaline speeds up the process of
> running an analysis by streamlining setting up commands, inputs, and
> output---which is where we spend most of our time in bioinformatics.
> Once the user has mastered the workflow, analysis of a dataset is rapid
> because so many of the tedious tasks of managing the commands, inputs,
> and outputs are taken care of.

Minor Issue #3: terms such as "meta-analysis", "reproducibility", "metadata" could be added.

> We have added some of these terms as keywords and made sure to reference
> these concepts throughout the manuscript.

Minor Issue #4: In line Information gained... resource management it would be nice to add references for the value of the method in each of the various fields mentioned.

> We have added references to Halfvarson et al. (2017) and Thomson and
> Willerslev (2015) to the background section that describe how
> metabarcoding information informs microbiome research and ecosystem
> conservation/management.

Minor Issue #5: Usually, (shotgun) metagenome analyses are used to measure diversity in microbiomes, meaning the functional, genomic diversity; the term microbiome has been widely used as the collection of genomes from all microbial taxa present in a sample. It would be better

to rephrase this like "popular method of measuring taxonomic/microbial diversity of host microbiome or in environmental samples"

> We have clarified this in the background section.

Minor Issue #6: As an overall comment, long sentences make the manuscript hard to read. In this case: "PCR primers have been used to generate amplicons of the bacterial 16S rRNA gene in studies of human and animal microbiota [..] among others." should be splitted.

> We have modified this sentence (and other long sentences) to improve
> readability throughout the manuscript.

Minor Issue #7: "other environmental surveys" please explain.

> We have removed the term "other environmental surveys".

Minor Issue #8: It is not clear to me how the study of Prodan et al. (2020) is related to the standardization of amplicon data analysis.

> We agree and have removed this reference.

Minor Issue #9: The authors highlight that the standard directory structure enhances data exploration and parameter optimization. A use case to demonstrate this main feature of Tourmaline would be of high value.

> We thank the reviewer for this suggestion and have added a example use
> case in the section "Helper scripts & parameter optimization". We have
> also noted how the Jupyter notebooks take advantage of Tourmaline's
> defined directory structure.

Minor Issue #10: The purpose of performing amplicon sequencing or metabarcoding is to reveal patterns of diversity in biological systems. That is not the only case, please rephrase.

> We have amended this sentence to include other outcomes of amplicon
> sequencing.

Close
