# Supplementary material for: Tourmaline: A containerized workflow for rapid and iterable amplicon sequence analysis using QIIME 2 and Snakemake
Source: Gigascience. 2022 Jul 28;11:giac066. doi: 10.1093/gigascience/giac066 (PMC9334028; doi:10.1093/gigascience/giac066)
Supplement: giac066_Response_to_Reviewer_Comments_Revision_1 [file giac066_response_to_reviewer_comments_revision_1.pdf]

Author's Response To Reviewer Comments

Close

Reviewer #2:

Dear authors,

I had the chance to review your revised manuscript on the Tourmaline wrapper.

Most of my initial comments have been addressed to some extent.

> We thank the reviewer for their prompt review of our revised manuscript and for their positive feedback on the revisions.

However, it is my belief that there is a major contradiction with this manuscript. As described in the introduction, Tourmaline is supposed to address challenges that make meta-analysis hard for metabarcoding studies: “this lack of automation and standardization is inefficient and creates barriers to meta-analysis and sharing of results.”

However, as the authors highlight in their response, there are 5 points that make Tourmaline to set apart from other amplicon workflows. However, only one of them ("3- Snakemake features") is related (to some extent) to the challenge described. The rest are exceptional ways to make things easier for the users to run an analysis but they do not have a direct link with how to enable/support meta-analysis.

Therefore, it is my belief that the Introduction section should be revised to better present the actual highlights of Tourmaline or further features (some of them described in my initial review) need to be added to support meta-analysis.

> We agree that, while Tourmaline can facilitate meta-analysis, this was not the primary motivation for developing Tourmaline. Therefore, as suggested by the reviewer, we have revised the Background (paragraphs 2–3) to focus on the full breadth of Tourmaline’s features and the motivation for including them.

Other Issues:

• Even though the authors recognize the impact of metadata standards, they do not mention anything on their manuscript about them and their potential I was not able to figure out how "have made the metadata that comes with Tourmaline fully MIMARKS-compliant." If this software is focused on meta-analysis, I would strongly suggest investing more effort on describing how these could benefit the community and the Tourmaline users.

> We regret this oversight in not mentioning our use and support of the MIMARKS metadata standard. We have added the following text to these sections:
> - Background: Added the following reference after the statement “standardizing analysis methods and metadata formats is much more feasible”: Vangay, P. et al. Microbiome Metadata Standards: Report of the National Microbiome Data Collaborative’s Workshop and Follow-On Activities. mSystems 6, (2021).
> - Background: “the [HTML] report facilitates evaluation of metadata (e.g., compliance with standards) and output (e.g., statistics about representative sequences and feature tables).”
> - Section on input files: “We recommend formatting metadata following the MIMARKS standard (Yilmaz et al., 2011), and we have done so in the metadata file included with the test dataset using the MIMARKS ‘water’ environmental package.”
> - Section on the output report: “Whether metadata are compliant with metadata standards such as MIMARKS can be easily detected by viewing the metadata summary in the report, which lists each metadata column and its most common value.”

• In the parallelization section that was added, it is fundamental to mention that this is possible thanks

to Qiime2 implementation. Snakemake is working as an interface allowing Tourmaline to support the options of Qiime2. If Qiime2 had no option for running on multiple threads, then Tourmaline would not inherit such a feature. The same applies for the merging step in the meta-analysis case. All Qiime2 commands used as such need to be clear that are Qiime2 commands that are performed in the Tourmaline workflow; otherwise it can be thought that the feature was developed from the Tourmaline team.

> We agree that it is important to recognize the QIIME 2 development team for adding support for parallelization and have added this sentence to the beginning of the section on parallelization: “Thanks to efforts of developers of QIIME 2 and other software, Tourmaline supports multiple cores in steps that support them, including denoising, feature classification, multiple sequence alignment, tree building, and core diversity calculations.”

Close
