# Supplementary material for: Tourmaline: A containerized workflow for rapid and iterable amplicon sequence analysis using QIIME 2 and Snakemake
Source: Gigascience. 2022 Jul 28;11:giac066. doi: 10.1093/gigascience/giac066 (PMC9334028; doi:10.1093/gigascience/giac066)
Supplement: giac066_Response_to_Reviewer_Comments_Revision_2 [file giac066_response_to_reviewer_comments_revision_2.pdf]

Author's Response To Reviewer Comments

Close

Point-by-point Response
Editor decision (April 13, 2022)

Dear Dr. Thompson,

Your manuscript "Tourmaline: a containerized workflow for rapid and iterable amplicon sequence analysis using QIIME 2 and Snakemake" (GIGA-D-21-00281R2) is acceptable for publication in GigaScience, in principle.

> We are pleased to learn that our manuscript would in principle be acceptable for publication in GigaScience. We have carefully addressed each of the remaining points, as described below.

However, before we proceed, please address the following points:

1) We have discussed the submission again with one of the reviewers and with the GigaScience editorial team, and in light of this, we feel the manuscript could be structured even more around the challenge described in the abstract: "lack of automation and standardization is inefficient and creates barriers to meta-analysis and sharing of results" - and emphasize in a bit more detail how the tool contributes to addressing this challenge in introduction and conclusion.

> We thank the reviewer and editorial team for helping us further improve the clarity and impact of this manuscript. As suggested, we have provided additional focus on how Tourmaline uses automation and standardization to support meta-analysis and sharing. Some examples of this increased emphasis include, in the abstract: "Outputs are stored in a defined directory structure that is the same for every Tourmaline run, facilitating data exploration, parameter optimization, downstream analysis, and meta-analysis across studies." And: "A zipped run directory can be shared with collaborators, and relative links in the report are preserved, facilitating data exploration by experts and non-experts alike." In the conclusions: "Visualizations and reports—ready to share. Every Tourmaline run produces an HTML report containing a summary of metadata and outputs, with links to web-viewable QIIME 2 visualization files. Zipped run directories can be shared with collaborators, with relative links in the report allowing easy access to the visualizations and other output files." And: "Meta-analysis. The standardized input and output file names and directory structure facilitate meta-analysis of multiple studies that have been analyzed through Tourmaline. The provided meta-analysis Jupyter notebook, written in Python, uses Pandas and the QIIME 2 Artifact API and provides a starting point for combining and co-analyzing the output of multiple Tourmaline runs."

2) Please move all URLs (e.g. github repositories, software sites, also youtube video URLS etc.) from the main text to the bibliography, and cite them by number from the text - we treat internet resources as citable items. please refer to our instructions for authors for formatting guidelines.

> All URLs except one in the abstract have been moved to the references section.

3) Please register any new software application in the bio.tools and SciCrunch.org databases to receive RRID (Research Resource Identification Initiative ID) and biotoolsID identifiers, and include these in your manuscript, in the "code availability" section. This will facilitate tracking, reproducibility and re-use of your tool.

> We have registered the software with bio.tools and SciCrunch.org. The IDs are contained in the code availability section.

4) Please fill out the list below and include it in your "code availability" section:

Availability of supporting source code and requirements

Project name: e.g. My bioinformatics project
Project home page: e.g. https://github.com/ISA-tools
Operating system(s): e.g. Platform independent
Programming language: e.g. Java
Other requirements: e.g. Java 1.3.1 or higher, Tomcat 4.0 or higher
License: e.g. GNU GPL, FreeBSD etc.
RRID: , e.g. RRID: SCR_014986
biotools ID: e.g. XYZTool

> All fields have been completed and added to the code availability section.

> Project name: Tourmaline
> Project home page: https://github.com/aomlomics/tourmaline
> Operating system(s): Linux, macOS
> Programming language: Python
> Other requirements: Conda or Docker
> License: 3-clause BSD license
> RRID: SCR_022465
> bio.tools ID: tourmaline

5) Please add ORCIDs to the title page (e.g. as a list under "affiliations" - the production team will do the formatting)

ORCID IDs: Luke R Thompson [0000-0002-3911-1280]; Sean R Anderson [0000-0003-3096-1120]; Paul A D Uyl [0000-0003-3328-3476]; Nastassia V Patin [0000-0001-8522-7682]; Grant Sanderson [0000-0003-3565-1949]; Kelly D. Goodwin [0000-0001-9583-8073];

> We have added the ORCIDs to the document.

Close
