# Supplementary material for: Tourmaline: A containerized workflow for rapid and iterable amplicon sequence analysis using QIIME 2 and Snakemake
Source: Gigascience. 2022 Jul 28;11:giac066. doi: 10.1093/gigascience/giac066 (PMC9334028; doi:10.1093/gigascience/giac066)
Supplement: giac066_Reviewer_1_Report_Original_Submission — Anna Heintz-Buschart -- 10/11/2021 Reviewed [file giac066_reviewer_1_report_original_submission.pdf]

Reviewer Report

Title: Tourmaline: a containerized workflow for rapid and iterable amplicon sequence analysis using QIIME 2 and Snakemake

Version: Original Submission **Date:** 10/11/2021

Reviewer name: Anna Heintz-Buschart

Reviewer Comments to Author:

Thompson et al. present a workflow for amplicon sequencing analysis, wrapping commands from the commonly used QIIME2 package in the commonly used workflow manager Snakemake. The manuscript is clearly structured and contains figures of appropriate quality. Building and testing user-friendly workflows that facilitate the use of existing software is an important task for the research community. The chosen existing softwares (namely Snakemake as workflow manager and QIIME2's calls to DADA2 and deblur, as well as QIIME2's visualisations) are trusted and often used by the community. Next to manuscript, I have inspected the GitHub/wiki page of the proposed workflow and tested it on the provided test data as well as an independent data set. I have run into some issues, which I will put forward below, together with some comments on the content of and omissions from the manuscript.
Manuscript:
1) Overall, the reads more like a manual or tutorial than a methods description. The point-by-point description of the outputs may be a bit lengthy.
2) The manuscript is missing information on runtimes and hardware requirements. This is in particular a pity because the workflow does not make use of parallelisation of the called tools. It might be pretty slow on large datasets?
3) There is also no justification for the choices of the analyses that are done and the defaults that were chosen.
4) In the introduction, other published amplicon sequencing workflows are cited and dismissed as not all well documented. Other than maybe not being so well documented, there are differences in scope between these workflows and the one described here. It would be very helpful for readers to be informed on how the described workflow is set apart from those workflows. And also from QIIME2 (all of the images in figure 3, for example, are QIIME2's visualisation work and not part of the workflow's report). Finally, tagseq, which also wraps QIIME2 commands in snakemake, is not mentioned. From my point of view, the workflow still requires the user to be able to do quite a lot of data setup in the command line environment and requires knowledge of QIIME2, while resolving relatively little by wrapping the commands in Snakemake (also see my next point). Clearly, it would be helpful to discuss what existing problem the workflow overcomes that the others (and QIIME2) don't.
5) In the same paragraph of the introduction, it is mentioned that the workflow might evolve with QIIME2. However, it makes use of only a small part of QIIME's options/commands - is there a plan to widen the scope? How will continuous support be done? Is there a plan to integrate the workflow into the QIIME software ecosystem? Similarly, the workflow is not using Snakemake to its full potential, e.g. it does require several manual installation steps instead of making use of Snakemake's conda

integration; it doesn't make use of Snakemake's reporting ability, which might be interesting together with QIIME2's data provenance. So, is there a plan to improve this? Also for developing it towards better usability on (cloud) cluster structures?
Testruns:
What worked: Overall, I could install the software on a linux machine by following the description on the GitHub webpage. The test run worked as expected. The data was accessible and I could visualise it using the QIIME2 online viewer. I could run the workflow on an unrelated dataset.
What could be improved:
a) The setup of the input was a bit annoying, because the names and paths to the inputs and outputs need to be set at various places. The fact that existing and non-existing inputs have to be defined in the config confused me at first. The error messages that ensued from not doing this right were uninformative (these cases could be caught by the Snakefile with or without the help of a scheme).
b) while the workflow is very well documented, the settings for the individual demonising / taxonomy steps are not. The links to the QIIME2 documentation don't point to the current version.
What didn't work: Running a small dataset - the workflow expects to be able to do statistical test with groups and replicates. However, only a late step checks if the data set is suitable, so there-s a failure after considerable running time, which is annoying. While this kind of analysis may be the most common application, it's not the only one. It would be good if those parts of the workflow that require certain dataset structures could be switched off.
minor:
i) As a very irregular user of QIIME2, I find the QIIME2-jargon difficult to understand (e.g. artefacts and artefact equivalents, manifesto, and the QIIME2 names of the DADA2 and deblur steps, emperor plot...). It would be better, if these were defined (and maybe not all discussed in detail).
ii) Personally, I would like to have a primer removal step in the workflow. But that's a design decision that can be discussed.
iii) "the fungal internal transcribed spacer (ITS) of the rRNA gene (Abarenkov et al. 2010)" - the internal transcribed spacer is not within an rRNA gene, but the different ITS regions are found between rRNA genes.

Level of Interest

Please indicate how interesting you found the manuscript: Choose an item.

Quality of Written English

Please indicate the quality of language in the manuscript: Choose an item.

Declaration of Competing Interests

Please complete a declaration of competing interests, considering the following questions:

- Have you in the past five years received reimbursements, fees, funding, or salary from an organisation that may in any way gain or lose financially from the publication of this manuscript, either now or in the future?
- Do you hold any stocks or shares in an organisation that may in any way gain or lose financially from the publication of this manuscript, either now or in the future?
- Do you hold or are you currently applying for any patents relating to the content of the manuscript?
- Have you received reimbursements, fees, funding, or salary from an organization that holds or has applied for patents relating to the content of the manuscript?
- Do you have any other financial competing interests?
- Do you have any non-financial competing interests in relation to this paper?

If you can answer no to all of the above, write 'I declare that I have no competing interests' below. If your reply is yes to any, please give details below.

I declare that I have no competing interests.

I agree to the open peer review policy of the journal. I understand that my name will be included on my report to the authors and, if the manuscript is accepted for publication, my named report including any attachments I upload will be posted on the website along with the authors' responses. I agree for my report to be made available under an Open Access Creative Commons CC-BY license (http://creativecommons.org/licenses/by/4.0/). I understand that any comments which I do not wish to be included in my named report can be included as confidential comments to the editors, which will not be published.

Choose an item.

To further support our reviewers, we have joined with Publons, where you can gain additional credit to further highlight your hard work (see: https://publons.com/journal/530/gigascience). On publication of this paper, your review will be automatically added to Publons, you can then choose whether or not to claim your Publons credit. I understand this statement.

Yes Choose an item.
