# Supplementary material for: Tourmaline: A containerized workflow for rapid and iterable amplicon sequence analysis using QIIME 2 and Snakemake
Source: Gigascience. 2022 Jul 28;11:giac066. doi: 10.1093/gigascience/giac066 (PMC9334028; doi:10.1093/gigascience/giac066)
Supplement: giac066_Reviewer_2_Report_Original_Submission — Haris Zafeiropoulos -- 10/13/2021 Reviewed [file giac066_reviewer_2_report_original_submission.pdf]

Reviewer Report

Title: Tourmaline: a containerized workflow for rapid and iterable amplicon sequence analysis using QIIME 2 and Snakemake

Version: Original Submission **Date:** 10/13/2021

Reviewer name: Haris Zafeiropoulos

Reviewer Comments to Author:

Dear authors,
First of all, my apologies for the delay in the submission of the review.
I appreciated the opportunity to review your manuscript. Tourmaline aims at facilitating an easy-to-follow architecture for tracking input and output file names, parameters, and commands of QIIME2 runs to enhance meta-analyses. If I am not mistaken, this is the corner-stone of this study so my review is based on that.
Running Tourmaline is straightforward and its documentation is exceptional. The video tutorial and the GitHub wiki (https://github.com/aomlomics/tourmaline/wiki) allows non-experienced users to start working their analysis and the containerized version of the tool allows an easy-to-go installation in multiple operating systems without extra effort. The extra visual components provide insight in a nice way and the report returned can provide added value on the runs.
However, even if I do share the authors' interest on usability and interoperability and tools could have a great impact in the community indeed, Tourmaline currently lacks any substantial features to be considered as a stand-alone software tool. In addition, there are several issues that it is my belief that need to be addressed (see the following list).
Major issues
Major Issue #1: The authors claim that "this lack of automation and standardization [in tracking input and output file names, parameters, and commands on QIIME2] is inefficient and creates barriers to meta-analysis and sharing of results. Therefore, what Tourmaline and thus, the manuscript needs to demonstrate, is that meta-analyses are now feasible to a greater extent, thanks to the Tourmaline wrapper.
Major Issue #2: Assuming that enhancing meta-analyses is the main contribution of Tourmaline, it is fundamental to consider the minimum information about a marker gene sequence (MIMARKS) standard of the Genomic Standards Consortium (GSC). Rather than just mentioning MIMARKS, Tourmaline needs to explore ways to exploit such standards, i.e. perhaps by adding MIMARKS columns in the config file.
Major Issue #3: As QIIME2 has been developed on the basis of a plugin archiiated the opportunity to review your manuscript. Tourmaline aims at facilitating an easy-to-follow architecture for tracking input and output file names, parameters, and commands of QIIME2 runs to enhance meta-analyses. If I am not mistaken, this is the corner-stone of this study so my review is based on that.
tecture, it would be highly recommended that such an application could be provided as a plugin too, joining the corresponding QIIME2 library (https://library.qiime2.org/plugins/).
Major Issue #4: With respect to the structure of the manuscript, it is my belief that there are sections

that should be omitted. Tutorials and "how to" are of extremely valuable but it would be better to be provided either as supplementary material or through repositories, e.g. GitHub wiki, GitHub pages etc, rather than in the main manuscript. The wiki page on Tourmaline's GitHub repository is rather informative. An alternative might be merging the "Overview" along with the "Snakefile", "Config file", "Input files" and "Run the workflow" sections, to describe "The Tourmaline workflow" architecture in a less verbose way, highlighting the role of the "Snakefile" and the "config.yml" files and the architecture that binds them together. "Documentation", "Installation", "Cloning" subsections could/should be omitted too.
Major Issue #5: The test dataset does not allow the validation of Tourmaline in meta-analyses. It is rather important to have a testbed dataset to demonstrate "how to run" but a use case of an actual meta-analysis is required to demonstrate how different analyses can be combined in the framework of Tourmaline and provide further insight than those of the initial ones.
Major Issue #7: No license has been included in the "Availability of supporting source code" section. On Tourmaline GitHub repo a license (https://github.com/aomlomics/tourmaline#license) is mentioned, yet GigaScience asks for an appropriate Open Source Initiative compliant license (https://opensource.org/licenses/category). In addition, I tried to find if the QIIME2 license is mentioned in a Tourmaline Docker container and I could not; if I am not mistaken that is required based on the QIIME2 license (https://github.com/qiime2/qiime2/blob/master/LICENSE). My apologies again in case of any misapprehension.
Major Issue #8: Parameter optimization is indeed one of the greatest challenges in metabarcoding bioinformatics analyses. However, it is not clear to me how by keeping the exact same names in your output files, will you be able to compare the results of the different runs.
Major Issue #9: I realise that the authors provide Figures 2 and 3 in a complementary way, presenting the visual component returned after each step. However, having a figure with 16 screenshots makes it hard for the reader to realize what is coming from QIIME2 and what from Tourmaline but most importantly does not highlight the added value that Tourmaline provides to such an analysis. It is my belief that Figure 2 could remain as it, while FIgure 3 should focus on the output components that are not provided by QIIME2 routines, but from Tourmaline functionalities. In case of a meta-analysis, this figure should highlight all the added value that using QIIME2 through the Tourmaline wrapper would provide.
Minor issues
Minor Issue #1: Please rephrase the Findings section in the abstract, so that it is clear that Tourmaline invokes QIIME2 routines to implement taxonomy assignment, perform analysis etc. It is required to state clearly what QIIME2 does and what are the extra features of Tourmaline throughout the manuscript.
Minor Issue #2: The conclusion you mention in the abstract is not in line with the scope of Tourmaline that was described earlier. Tourmaline does not accelerate the performance of QIIME2 routines. Its aim, as mentioned earlier, is to enhance meta-analysis and sharing of results.
Minor Issue #3: terms such as "meta-analysis", "reproducibility", "metadata" could be added
Minor Issue #4: In line Information gained… resource management it would be nice to add references for the value of the method in each of the various fields mentioned.
Minor Issue #5: Usually, (shotgun) metagenome analyses are used to measure diversity in microbiomes,

meaning the functional, genomic diversity; the term microbiome has been widely used as the collection of genomes from all microbial taxa present in a sample. It would be better to rephrase this like "popular method of measuring taxonomic/microbial diversity of host microbiome or in environmental samples"
Minor Issue #6: As an overall comment, long sentences make the manuscript hard to read. In this case: "PCR primers have been used to generate amplicons of the bacterial 16S rRNA gene in studies of human and animal microbiota [..] among others." should be splitted.
Minor Issue #7: "other environmental surveys" please explain.
Minor Issue #8: It is not clear to me how the study of Prodan et al. (2020) is related to the standardization of amplicon data analysis.
Minor Issue #9: The authors highlight that the standard directory structure enhances data exploration and parameter optimization. A use case to demonstrate this main feature of Tourmaline would be of high value.
Minor Issue #10: The purpose of performing amplicon sequencing or metabarcoding is to reveal patterns of diversity in biological systems. That is not the only case, please rephrase.
Sincerely,
Haris Zafeiropoulos

Level of Interest

Please indicate how interesting you found the manuscript: Choose an item.

Quality of Written English

Please indicate the quality of language in the manuscript: Choose an item.

Declaration of Competing Interests

Please complete a declaration of competing interests, considering the following questions:

- Have you in the past five years received reimbursements, fees, funding, or salary from an organisation that may in any way gain or lose financially from the publication of this manuscript, either now or in the future?
- Do you hold any stocks or shares in an organisation that may in any way gain or lose financially from the publication of this manuscript, either now or in the future?
- Do you hold or are you currently applying for any patents relating to the content of the manuscript?
- Have you received reimbursements, fees, funding, or salary from an organization that holds or has applied for patents relating to the content of the manuscript?
- Do you have any other financial competing interests?
- Do you have any non-financial competing interests in relation to this paper?

If you can answer no to all of the above, write 'I declare that I have no competing interests' below. If your reply is yes to any, please give details below.

I declare that I have no competing interests

I agree to the open peer review policy of the journal. I understand that my name will be included on my report to the authors and, if the manuscript is accepted for publication, my named report including any attachments I upload will be posted on the website along with the authors' responses. I agree for my report to be made available under an Open Access Creative Commons CC-BY license (http://creativecommons.org/licenses/by/4.0/). I understand that any comments which I do not wish to be included in my named report can be included as confidential comments to the editors, which will not be published.

Choose an item.

To further support our reviewers, we have joined with Publons, where you can gain additional credit to further highlight your hard work (see: https://publons.com/journal/530/gigascience). On publication of this paper, your review will be automatically added to Publons, you can then choose whether or not to claim your Publons credit. I understand this statement.

Yes Choose an item.
