# Supplementary material for: Tourmaline: A containerized workflow for rapid and iterable amplicon sequence analysis using QIIME 2 and Snakemake
Source: Gigascience. 2022 Jul 28;11:giac066. doi: 10.1093/gigascience/giac066 (PMC9334028; doi:10.1093/gigascience/giac066)
Supplement: giac066_Reviewer_2_Report_Revision_1 — Haris Zafeiropoulos -- 3/7/2022 Reviewed [file giac066_reviewer_2_report_revision_1.pdf]

Reviewer Report

Title: Tourmaline: a containerized workflow for rapid and iterable amplicon sequence analysis using QIIME 2 and Snakemake

Version: Revision 1 **Date:** 3/7/2022

Reviewer name: Haris Zafeiropoulos

Reviewer Comments to Author:

Dear authors,
I had the chance to review your revised manuscript on the Tourmaline wrapper.
Most of my initial comments have been addressed to some extent.
However, it is my belief that there is a major contradiction with this manuscript. As described in the introduction, Tourmaline is supposed to address challenges that make meta-analysis hard for metabarcoding studies. "; this lack of automation and standardization is inefficient and creates barriers to meta-analysis and sharing of results".
However, as the authors highlight in their response, there are 5 points that make Tourmaline to set apart from other amplicon workflows. However, only one of them ("3- Snakemake features") is related (to some extent) to the challenge described. The rest are exceptional ways to make things easier for the users to run an analysis but they do not have a direct link with how to enable/support meta-analysis.
Therefore, it is my belief that the Introduction section should be revised to better present the actual highlights of Tourmaline or further features (some of them described in my initial review) need to be added to support meta-analysis.
Other Issues
Even though the authors recognize the impact of metadata standards:
they do not mention anything on their manuscript about them and their potential
I was not able to figure out how "have made the metadata that comes with Tourmaline fully MIMARKS-compliant."
If this software is focused on meta-analysis, I would strongly suggest investing more effort on describing how these could benefit the community and the Tourmaline users.
In the parallelization section that was added, it is fundamental to mention that this is possible thanks to Qiime2 implementation. Snakemake is working as an interface allowing Tourmaline to support the options of Qiime2. If Qiime2 had no option for running on multiple threads, then Tourmaline would not inherit such a feature. The same applies for the merging step in the meta-analysis case. All Qiime2 commands used as such need to be clear that are Qiime2 commands that are performed in the Troumaline workflow; otherwise it can be thought that the feature was developed from the Tourmaline team

Level of Interest

Please indicate how interesting you found the manuscript: Choose an item.

Quality of Written English

Please indicate the quality of language in the manuscript: Choose an item.

Declaration of Competing Interests

Please complete a declaration of competing interests, considering the following questions:

- Have you in the past five years received reimbursements, fees, funding, or salary from an organisation that may in any way gain or lose financially from the publication of this manuscript, either now or in the future?
- Do you hold any stocks or shares in an organisation that may in any way gain or lose financially from the publication of this manuscript, either now or in the future?
- Do you hold or are you currently applying for any patents relating to the content of the manuscript?
- Have you received reimbursements, fees, funding, or salary from an organization that holds or has applied for patents relating to the content of the manuscript?
- Do you have any other financial competing interests?
- Do you have any non-financial competing interests in relation to this paper?

If you can answer no to all of the above, write 'I declare that I have no competing interests' below. If your reply is yes to any, please give details below.

I declare that I have no competing interests

I agree to the open peer review policy of the journal. I understand that my name will be included on my report to the authors and, if the manuscript is accepted for publication, my named report including any attachments I upload will be posted on the website along with the authors' responses. I agree for my report to be made available under an Open Access Creative Commons CC-BY license (http://creativecommons.org/licenses/by/4.0/). I understand that any comments which I do not wish to be included in my named report can be included as confidential comments to the editors, which will not be published.

Choose an item.

To further support our reviewers, we have joined with Publons, where you can gain additional credit to further highlight your hard work (see: https://publons.com/journal/530/gigascience). On publication of this paper, your review will be automatically added to Publons, you can then choose whether or not to claim your Publons credit. I understand this statement.

Yes Choose an item.
